# Broad-Spectrum Legionaminic Acid-Specific Antibodies in Pooled Human IgGs Revealed by Glycan Microarrays with Chemoenzymatically Synthesized Nonulosonosides

**DOI:** 10.3390/molecules29163980

**Published:** 2024-08-22

**Authors:** Anoopjit Singh Kooner, Hai Yu, Shani Leviatan Ben-Arye, Vered Padler-Karavani, Xi Chen

**Affiliations:** 1 Department of Chemistry, University of California, Davis, CA 95616, USA; akooner@ucdavis.edu (A.S.K.); hyu@ucdavis.edu (H.Y.); 2 Department of Cell Research and Immunology, The Shmunis School of Biomedicine and Cancer Research, The George S. Wise Faculty of Life Sciences, Tel Aviv University, Tel Aviv 69978, Israel; leviata@tauex.tau.ac.il

**Keywords:** bacterial nonulosonic acid, carbohydrate, chemoenzymatic synthesis, legionaminic acid, human antibodies

## Abstract

The presence and the level of antibodies in human sera against bacterial glycans are indications of prior encounters with similar antigens and/or the bacteria that express them by the immune system. An increasing number of pathogenic bacteria that cause human diseases have been shown to express polysaccharides containing a bacterial nonulosonic acid called 5,7-di-*N*-acetyllegionaminic acid (Leg5,7Ac_2_). To investigate the immune recognition of Leg5,7Ac_2_, which is critical for the fight against bacterial infections, a highly effective chemoenzymatic synthon strategy was applied to construct a library of α2–3/6-linked Leg5,7Ac_2_-glycans via their diazido-derivatives (Leg5,7diN_3_-glycans) formed by efficient one-pot three-enzyme (OP3E) synthetic systems from a diazido-derivative of a six-carbon monosaccharide precursor. Glycan microarray studies using this synthetic library of a Leg5,7Ac_2_-capped collection of diverse underlying glycan carriers and their matched sialoside counterparts revealed specific recognition of Leg5,7Ac_2_ by human IgG antibodies pooled from thousands of healthy donors (IVIG), suggesting prior human encounters with Leg5,7Ac_2_-expressing pathogenic bacteria at the population level. These biologically relevant Leg5,7Ac_2_-glycans and their immune recognition assays are important tools to begin elucidating their biological roles, particularly in the context of infection and host–pathogen interactions.

## 1. Introduction

**Figure 1:**
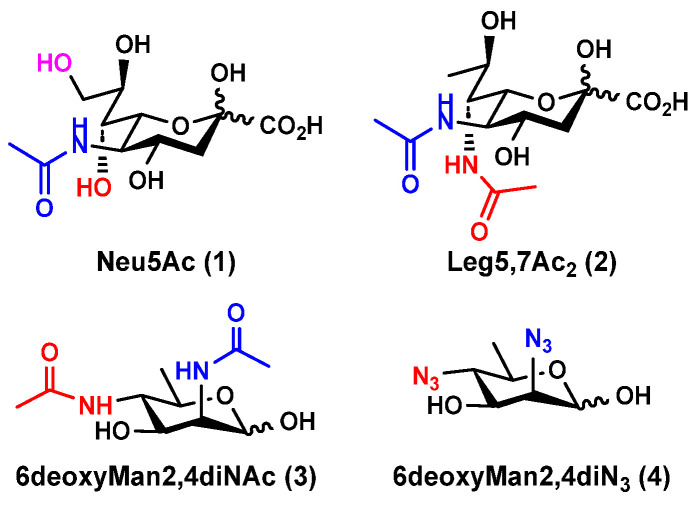
Structures of *N*-acetylneuraminic acid (Neu5Ac, **1**); 5,7-di-*N*-acetyllegionaminic acid (Leg5,7Ac_2_, **2**); Leg5,7Ac_2_ precursor 6deoxyMan2,4diNAc (**3**); and Leg5,7Ac_2_ chemoenzymatic synthon 6deoxyMan2,4diN_3_ (**4**).

Human sera contain protective antibodies against a large range of carbohydrate epitopes expressed by different species [1,2,3,4]. These antibodies are developed either naturally or in response to infections, vaccinations, or other exposures to antigens. Human antibodies recognizing bacterial glycans have been revealed by profiling human sera and pooled human immunoglobulins against bacterial surface carbohydrate antigens immobilized on glycan microarrays [5]. Synthetic glycans with defined structures are important probes for these studies. Access to such synthetic glycans, especially those with complex structures that are challenging to obtain either by purification from natural sources or by chemical synthesis, is greatly facilitated by the development of efficient chemoenzymatic methods. Glycans and glycoconjugates containing nine-carbon α-keto acids called nonulosonic acids (NulOs) [6] are among the challenging synthetic targets [7]. The most well-known NulOs are sialic acids (Sias) based on a neuraminic acid (Neu) or a 2-keto-3-deoxy-nononic acid (Kdn) backbone in the deuterostome lineage of animals and in certain bacteria. In vertebrates, Sias are often presented on the termini of glycans on cell surface glycoconjugates and *N*-acetylneuraminic acid (Neu5Ac, **1**, Figure 1) is the most common and well-studied sialic acid form.

Bacterial nonulosonic acid (NulO) 5,7-di-*N*-acetyllegionaminic acid (Leg5,7Ac_2_, **2**) (Figure 1), or 5,7-diacetamido-3,5,7,9-tetradeoxy-D-*glycero*-D-*galacto*-non-2-ulosonic acid [6,8], is an analog of Neu5Ac (**1**). Leg5,7Ac_2_ and Neu5Ac share an identical D-*glycero*-D-*galacto*-backbone configuration but differ for the functional groups at C7 and C9 positions. Leg5,7Ac_2_ is the 7-acetamido-7,9-dideoxy derivative of Neu5Ac by replacing its C7-OH with an acetamido group and its C9-OH with a hydrogen atom (Figure 1) [9,10].

Chemical synthesis of Leg5,7Ac_2_ from 2,4-diacetamido-2,4,6-trideoxy-D-mannose (6deoxyMan2,4diNAc, **3**) (Figure 1) and oxalacetic acid under basic conditions was initially reported in 2001 [11,12]. The synthetic Leg5,7Ac_2_ was used to facilitate the identification and the correction of Leg5,7Ac_2_-containing bacterial lipopolysaccharide (LPS) structures including those from *Legionella pneumophila* serogroup 1, a pathogen known to cause Legionnaire’s disease; *Acinetobacter baumannii*, a causative agent of fatal bacteremia; and *Pseudomonas fluorescens*, *Vibrio salmonicida*, and *Vibrio alginolyticus* [11,12]. Leg5,7Ac_2_ derivatives with variations on the stereochemistry of C4 and/or C8 and substitutions at C5 and/or C7 were also identified from bacterial sources [6,8,11,13].

The synthesis of glycans containing Leg5,7Ac_2_ and derivatives [14] by chemical methods has been challenging [15]. The chemical synthetic strategies reported involved long routes, low overall yields, and a lack of stereochemical control for the formation of the biologically relevant α-legionaminic acid glycosyl linkage found in several bacterial polysaccharides [15]. In addition, the obtained products were limited to monosaccharides [11,12,16] or glycosides of either monosaccharides [15,17] or disaccharides [18,19].

The biosynthetic processes for Leg5,7Ac_2_ and its cytidine 5′-monophosphate (CMP)-activated donor CMP-Leg5,7Ac_2_ for the presumed legionaminyltransferases were reported from *Legionella pneumophila* [20] and *Campylobacter jejuni* [21] in 2008 and 2009, respectively. Both processes formed 6deoxyMan2,4diNAc (**3**, Figure 1) as a key intermediate despite the differences in using its uridine 5′-diphosphate (UDP) [20] or guanosine 5′-diphosphate (GDP) [21]-activated precursor in these two bacteria. The related enzymes had been expressed in *E. coli* for the production of Leg5,7Ac_2_ and CMP-Leg5,7Ac_2_ [22,23]. Recently, a metabolic labeling method using azide-modified Leg precursors led to the identification of Maf4 as a putative flagellin legionaminyltransferase from *Campylobacter jejuni* [24]. Nevertheless, information regarding legionaminyltransferases is very limited in general.

Recombinant sialyltransferases identified from bacterial and mammalian sources have been used for enzymatic and/or chemoenzymatic synthesis of Leg5,7Ac_2_-glycans and derivatives. For example, bacterial *Pasteurella multocida* multifunctional α2–3-sialyltransferase PmST1 (previously named as tPm0188Ph [25]) [26] and *Neisseria meningitidis* MC58 α2–3-sialyltransferase [27], as well as porcine ST3Gal-I [26], were found to be efficient in forming α2–3-linked glycosides terminated with Leg5,7Ac_2_. In comparison, bacterial *Photobacterium damselae* α2–6-sialyltransferase was less efficient in the formation of α2–6-linked glycosides terminated with Leg5,7Ac_2_ [26] but its A235M mutant [27] had improved activity. Porcine ST3Gal-I was used to catalyze the transfer of Leg5,7Ac_2_ from CMP-Leg5,7Ac_2_ to a glycolipid GM1a to form a GD1a analog containing a terminal Leg5,7Ac_2_, which was not recognized by an Neu5Ac-terminated GD1a-binding protein, myelin-associated glycoprotein (MAG or Siglec-4) [28]. Porcine ST3Gal-I and human ST6Gal-I were suitable for catalyzing the transfer of Leg5,7Ac_2_ from CMP-Leg5,7Ac_2_ to the O-glycans and N-glycans, respectively, on the therapeutic glycoproteins interferon-α2b and α1-antitrypsin [28]. We previously reported the chemical synthesis of the important biosynthetic intermediate 6deoxyMan2,4diNAc (**3**) and its application as an enzymatic precursor for the direct formation of Leg5,7Ac_2_-glycosides [9] using a one-pot three-enzyme (OP3E) system containing *Pasteurella multocida* (PmAldolase), *Legionella pneumophila* CMP-Leg5,7Ac_2_ synthetase (LpCLS), and an α2–3-sialyltransferase such as *Pasteurella multocida* multifunctional α2–3-sialyltransferase 1 (PmST1) or its M144D mutant (PmST1_M144D) with decreased sialidase and donor hydrolysis activities [10]. Nevertheless, the production of Leg5,7Ac_2_-glycosidases by directly transferring Leg5,7Ac_2_ from CMP-Leg5,7Ac_2_ to appropriate acceptors using known sialyltransferases can be restricted by the types of acceptors that can be tolerated by a given sialyltransferase and the decreased efficiency in using CMP-Leg5,7Ac_2_ as the donor substrate.

To overcome the challenges, we developed a chemoenzymatic synthon strategy for the highly efficient synthesis of Leg5,7Ac_2_-glycosides [9]. In this strategy, a chemoenzymatic synthon 2,4-diazido-2,4,6-trideoxy-D-mannose (6deoxyMan2,4diN_3_, **4**) (Figure 1) was chemically synthesized from commercially available D-fucose in eight steps with an overall 59.5% yield. It was a well-suited precursor for the synthesis of the corresponding 5,7-diazido-legionaminic acid (Leg5,7diN_3_)-glycosides in 71–98% yields using a one-pot three-enzyme (OP3E) system containing PmAldolase, *Neisseria meningitidis* CMP-sialic acid synthetase (NmCSS), and an α2–3-sialyltransferase such as PmST1_M144D or an α2–6-sialyltransferase such as *Photobacterium* species α2–6-sialyltransferase (Psp2,6ST) [9]. The resulting Leg5,7diN_3_-glycosides were conveniently converted to the corresponding target Leg5,7Ac_2_-glycosides in 69–88% yields by treating with thioacetic acid in the presence of saturated sodium bicarbonate in aqueous solution [9]. Herein, we explore the application of the chemoenzymatic synthon 6deoxyMan2,4diN_3_ (**4**) for the synthesis of a comprehensive library of α2–3- and α2–6-linked Leg5,7Ac_2_-glycosides containing different underlying glycans. Glycan microarrays printed with this comprehensive collection of synthetic α2–3- and α2–6-linked Leg5,7Ac_2_-glycosides and their related sialoside pairs demonstrate that pooled human IgGs are rich with antibodies that selectively recognize this broad spectrum of Leg5,7Ac_2_-glycosides.

## 2. Results and Discussion

### 2.1. Chemoenzymatic Synthesis of Leg5,7Ac_2_-Glycosides

Our synthetic targets were α2–3- and α2–6-linked Leg5,7Ac_2_-glycosides containing different underlying glycans with an aglycon linker that can be used for immobilization on slides for glycan microarray assays. A propylamine aglycon was chosen as a well-suited design for the purpose. To produce the 5,7-diazido-legionaminic acid (Leg5,7diN_3_)-glycosides efficiently from the chemoenzymatic synthon 6-deoxyMan2,4diN_3_ (**4**) using OP3E sialyation systems [9] and allow efficient product purification, glycosides containing a hydrophobic carboxybenzyl (Cbz)-protected propylamine aglycon (ProNHCbz) [29] were chosen as sialyltransferase acceptors. Seven Cbz-tagged glycosides [30] including GalNAcαProNHCbz (Tn antigen, **5**), LacβProNHCbz (**6**), LacNAcβProNHCbz (**7**), Galβ1–3GalNAcβProNHCbz (**8**), Galβ1–3GalNAcαProNHCbz (Core 1, **9**), Galβ1–3GlcNAcβProNHCbz (Type I glycan, **10**), and Galβ1–3GlcNAcαProNHCbz (**11**) were used as sialyltransferase acceptors for OP3E sialylation reactions. The UV-detectability and the hydrophobicity of the ProNHCbz [29] in the glycoside acceptors and the resulting Leg5,7diN_3_-glycosylated products facilitate the reaction monitoring and product purification processes.

**Scheme 1:**
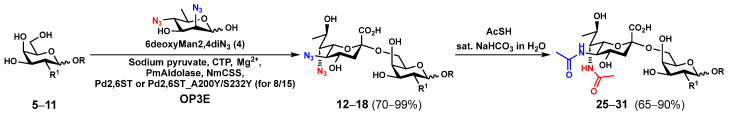
Chemoenzymatic synthesis of α2–6-linked Leg5,7Ac_2_-glycosides (**25**–**31**) via OP3E produced α2–6-linked Leg5,7diN_3_-glycosides (**12**–**18**).

As shown in Scheme 1, the OP3E system containing PmAldolase, NmCSS, and *Photobacterium damselae* α2–6-sialyltransferase (Pd2,6ST) or its A200Y/S232Y double mutant (Pd2,6ST_A200Y/S232Y) [31] was used to synthesize α2–6-linked Leg5,7diN_3_-glycosides (**12**–**18**) from 6deoxyMan2,4diN_3_ (**4**) and glycoside acceptors **5**–**11**. In this system, 6deoxyMan2,4diN_3_ (**4**) reacted with pyruvate by a PmAldolase-catalyzed reaction to form Leg5,7diN_3_, which was activated by NmCSS in the presence of cytidine 5′-triphosphate (CTP) and magnesium (Mg^2+^) cation to form the corresponding CMP-activated sugar nucleotide CMP-Leg5,7diN_3_ to allow the transfer of Leg5,7diN_3_ to the sialyltransferase acceptor used in the OP3E reaction to form the desired α2–6-linked Leg5,7diN_3_-glycoside. The target glycosides **12**–**18** (Table 1) were obtained with yields ranging from 70 to 99%, each was purified from the OP3E reaction with a single C18-cartridge purification process. When Galβ1–3GalNAcβProNHCbz (**8**) was used as the acceptor for Pd2,6ST in the OP3E, an undesired additional Leg5,7diN_3_ could be added to the penultimate GalNAc moiety in the acceptor substrate, which caused complications in purifying the desired product Leg5,7diN_3_α2–6Galβ1–3GalNAcβProNHCbz (**15**) and led to low yields. To our delight, the Pd2,6ST_A200Y/S232Y double mutant [31] was capable of adding Leg5,7diN_3_ regio-selectively to the terminal galactose (Gal) of **8** to produce **15** in an excellent 96% yield. On the other hand, Pd2,6ST in the OP3E was efficient in synthesizing other α2–6-linked Leg5,7diN_3_-glycoside targets (**12**–**14**, **16**–**18**). Treatment of the azido-containing glycosides (**12**–**18**) with thioacetic acid and saturated sodium bicarbonate aqueous solution [9,30] produced the target α2–6 linked Leg5,7Ac_2_-glycosides (**25**–**31**) in 65–90% yields.

**Scheme 2:**
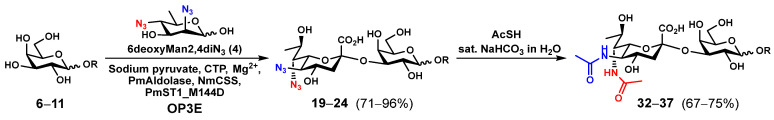
Chemoenzymatic synthesis of α2–3-linked Leg5,7Ac_2_-glycosides (**32**–**37**) via OP3E produced α2–3-linked Leg5,7diN_3_-glycosides (**19**–**24**).

Similarly, the OP3E system containing PmAldolase, NmCSS, and PmST1_M144D [32] was used for the efficient synthesis of α2–3-linked Leg5,7diN_3_-glycosides (**19**–**24**) from galactosides **6**–**11**, respectively, in 71–96% yields (Scheme 2). They were readily converted to the desired α2–3-linked Leg5,7Ac_2_-glycosides (**32**–**37**) in 67–75% yields using thioacetic acid in saturated sodium bicarbonate aqueous solution.

The Cbz group in Leg5,7Ac_2_-glycosides (**25**–**37**) was conveniently and completely removed by catalytic hydrogenation [33] to produce Leg5,7Ac_2_-glycosides containing a primary amino group on the propylamine aglycon, which was ready for immobilization on *N*-hydroxysuccinimide (NHS)-ester-coated [34,35] or epoxy-coated surface [36,37,38,39] for glycan microarray studies. It could also be coupled to other molecules such as proteins [40] and/or biotin [41] for various functional studies.

### 2.2. Glycan Microarray Binding Studies Reveal a Broad Spectrum of Human IgG Antibodies against Leg5,7Ac_2_-Glycosides

**Figure 2:**
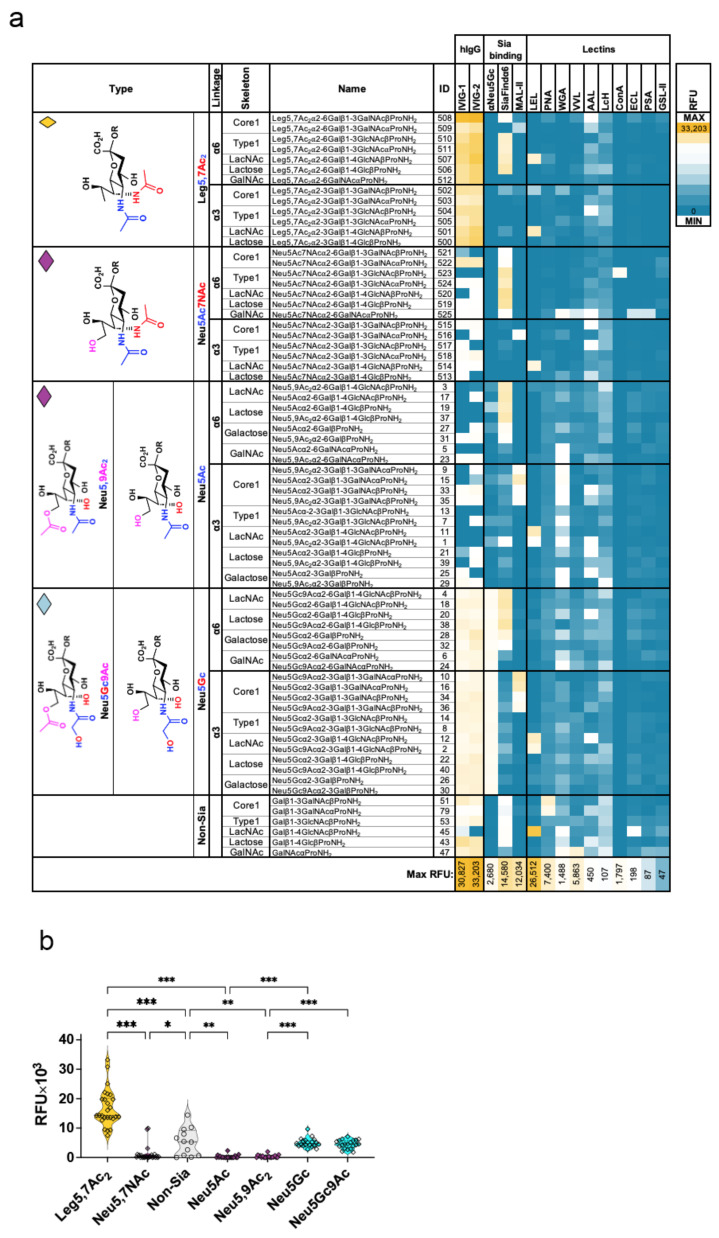
Glycan microarray binding studies against Leg5,7Ac_2_-glycans and sialoglycan counterparts. (**a**) Glycan microarrays fabricated with Leg5,7Ac_2_-glycans and sialoglycans (Neu5Gc/Neu5Ac/Neu5Ac7NAc-glycans) were examined with IVIG (IVIG-1 Privigen or IVIG-2 OMRIgG-AR; 50 μg/sub-array), anti-Neu5Gc IgY (αNeu5Gc; 1:7000), SiaFindα2-6 (0.25 μg), and 11 biotinylated plant lectins (2 μg each: MAL-II, LEL, PNA, WGA, VVL, AAL, LcH, ConA, ECL, PSA, and GSL-II) at 100 μL/subarray in blocking buffer (PBS, 1% ovalbumin; the assay buffers for plant lectins also contained divalent cations, see ESI), then detected with Cy3-anti-human IgG (0.4 ng/μL) for IVIG, Cy3-anti-chicken IgY (0.375 ng/μL) for IgY, and Cy3-sterptavidin (1.2 ng/μL) for Sia-Find and Lectins, at each 200 μL/sub-array. Slides were scanned and analyzed for Relative Fluorescence Units (RFU), which were plotted as a heatmap. (**b**) Binding of IVIG (average IVIG-1 and IVIG-2) to Leg5,7Ac_2_-glycans compared to Neu5Ac7NAc-glycans, non-Sia glycans, and glycans terminated with Neu5Ac, Neu5,9Ac2, Neu5Gc, and Neu5Gc9Ac (mixed effects analysis, with the Geisser-Greenhouse correction and individual variances computed for each comparison using uncorrected Fisher’s LSD; * *p* = 0.04, ** *p* = 0.001, *** *p* < 0.001).

**Figure 3:**
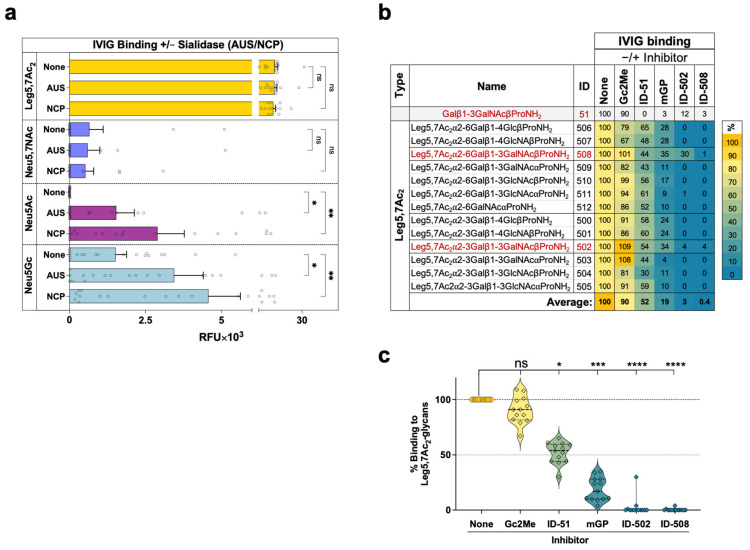
Characterization of IVIG binding to Leg5,7Ac_2_-glycans. (**a**) Binding of IVIG-2 (OMRIgG-AR) to glycan microarray at 50 μg/sub-array, after pre-treatment of the arrays with/without 10 mU sialidase (*Arthrobacter ureafaciens* sialidase, AUS; *Clostridium perfringens* neuraminidase; NCP) for 2 h at 37 °C, then detected with 0.4 ng/μL Cy3-anti-human IgG. Slides were scanned, analyzed, and RFU-plotted (mixed-effects analysis, with the Geisser-Greenhouse correction, and individual variances computed for each comparison using uncorrected Fisher’s LSD; ns = non-significant *p* > 0.05 (*p* = 0.6132, 0.3334, 0.7287, 0.7454 None vs AUS/NCP in Leg/Neu, respectively), * *p* = 0.02, ** *p* < 0.003). (**b**) Averaged binding of IVIG-1 and IVIG-2 to Leg5,7Ac_2_-glycans on glycan microarrays in the presence of selected competing glycans. IVIG (IVIG-1 Privigen or IVIG-2 OMRIgG-AR, 50 μg/sub-array) were pre-incubated for 2 h on ice with various inhibitors on each sub-array, including 2-*O*-methyl-αNeu5Gc (Gc2Me; 4 mM), glycan ID-51 (Core1β, 2 mM), mouse serum glycopeptides (mGP, containing *N*/*O*-glycans [46], 0.45 mM), glycan ID-502 (Leg5,7Ac_2_α3Core1β, 1 mM), and glycan ID-508 (Leg5,7Ac_2_α6Core1β; 1 mM). Then, binding of the pre-complexed IVIG was examined and detected with 0.4 ng/μL Cy3-anti-human IgG. Slides were scanned, analyzed, and RFU results were plotted. Percent binding to each glycan was calculated as a ratio to binding of IVIG without inhibitor (none, 100% binding). (**c**) Statistical analysis of inhibition of IVIG binding to Leg5,7Ac_2_-glycans of data presented in (**b**) (Friedman test with uncorrected Dunn’s test; ns *p* = 0.4631, * *p* = 0.0159, *** *p* = 0.0002, and **** *p* < 0.0001).

A previously reported glycan microarray study using β-glycosides of a Leg5,7Ac_2_ monosaccharide and its derivative that was chemically synthesized from D-threonine showed that human IgGs in three different samples of pooled human sera recognize the β-glycoside of Leg5,7Ac_2_ but not its 7-amino-derivative [15]. We used glycan microarrays to characterize the immune recognition of the biologically relevant propylamine aglycon-containing Leg5,7Ac_2_-α-glycosides obtained from **25**–**37** by catalytic hydrogenation to remove the Cbz group. These α-glycosides were fabricated onto epoxide-activated glass slides together with their matched pairs of sialoglycans synthesized previously, including those containing Neu5Ac [25,42], 9-*O*-acetyl-Neu5Ac (Neu5,9Ac_2_) [40,42], *N*-glycolylneuraminic acid (Neu5Gc) [40,42,43], 9-*O*-acetyl-Neu5Gc (Neu5Gc9Ac) [40,42,43], or 7-acetamido-7-deoxy-*N*-acetylneuraminic acid (Neu5Ac7NAc) [30]. Non-sialylated glycosides were also printed as control glycan probes. The resulting glycan microarrays were used to investigate the binding profiles of several antibodies and lectins (Figure 2 and Figure 3). Binding profiles of anti-Neu5Gc chicken immunoglobulin Y (IgY) [44], SiaFindα2–6, and *Maackia amurensis* lectin II (MAL-II) confirmed recognition of their known targets Neu5Gc-glycans, Siaα2–6-glycans, and Siaα2–3-glycans, respectively (Figure 2a). Interestingly, SiaFindα2–6 recognized all α2–6-linked glycans, including those with a terminal Leg5,7Ac_2_ or Neu5Ac7NAc, except those linked to GalNAc that were less preferred. In contrast, MAL-II preferred sialyl T-antigen (Siaα2–3Core1). Its binding was much reduced against Neu5Ac7NAc and completely lost against terminal Leg5,7Ac_2_ (Figure 2a). Similarly, most of the other lectins did not bind to Leg5,7Ac_2_-glycans, except for *Lycopersicon esculentum* (tomato) lectin (LEL, TL) that recognized α2–3LacNAc glycans with terminal Sia/Leg5,7Ac_2_ (Figure 2a). Importantly, human intravenous immunoglobulin G (IVIG; human IgG) pooled from thousands of individual donors, from two different sources (Privigen, OMRIgG-AR), showed strong binding to all Leg5,7Ac_2_-glycans, Neu5Gc-glycans, and non-sialylated glycans, but very low binding to Neu5Ac-glycans or Neu5Ac7NAc-glycans (Figure 2a). IVIG bound most non-sialylated glycans, but the least to LacNAc (ID-45). Among sialylated glycans, IVIG showed a strong preference for Neu5Gc over Neu5Ac, which was clear by comparing binding to the matched pairs of glycans that differ only by the single additional oxygen in C5 *N*-glycolyl versus *N*-acetyl (Figure 2a). While Neu5Gc is foreign to humans, Neu5Ac is a protective self-glycan and is not normally recognized by human antibodies, as previously described [45,46]. IVIG showed stronger binding to all Leg5,7Ac_2_-glycans compared to uncapped non-Sia underlying glycan counterparts. In contrast, there was no binding to their matched pairs of Neu5Ac7NAc-glycans, irrespective of their underlying glycan structures (Figure 2a,b). Thus, like human IgG preference toward Neu5Gc > Neu5Ac, binding of IVIG to Leg5,7Ac_2_ > Neu5Ac7NAc occurs despite their difference in only a single oxygen atom at the C9 position in Neu5Ac7NAc that is absent in Leg5,7Ac_2_ (Figure 2a,b). Together, these findings suggest that while Neu5Ac7NAc is less immunogenic, Leg5,7Ac_2_ is perceived as foreign, as indicated by human IgG binding, thus likely representing secondary immune responses after previous encounters with Leg5,7Ac_2_-expressing pathogens.

Sialic acids are known to belong to ‘self-associated molecular patterns’ (SAMPs) that provide a protective shield for glycoconjugates and cells [47], including from circulating antibodies [46]. To investigate human IgG immune recognition of Leg5,7Ac_2_ within the context of ‘pathogen-associated molecular patterns’ (PAMPs), enzymatic cleavage of these sialic acids analogs on the arrays was evaluated by two sialidases, *Arthrobacter ureafaciens* sialidase (AUS) and *Clostridium perfringens* neuraminidase (NCP), that peel-off terminal sialic acid moieties from glycans printed on the array. Both sialidases effectively removed Neu5Ac and Neu5Gc, exposing the underlying de-capped glycans to a robust binding of IVIG anti-glycan antibodies. However, all Leg5,7Ac_2_-glycans and Neu5Ac7NAc-glycans were found to be resistant to such cleavage by these two bacterial sialidases (Figure 3a), which correlated well with our previous results [9,48]. Despite the close similarity between Neu5Ac and Leg5,7Ac_2_, the glycans capped with the latter become resistant to sialidase cleavage, potentially providing an immunological advantage to a human invading pathogen that would be coated with such PAMP glycans. This is further supported by recent studies of *Campylobacter jejuni*, the leading cause of foodborne bacterial gastroenteritis, revealing that expression of legionaminic acid on its bacterial flagellin serves as a virulence factor supporting enhanced biofilm formation, auto-agglutination, and host colonization [24,49]. On the other hand, pathogens also commonly use SAMPs as a molecular mimicry mechanism to evade host immunity, particularly by sialic acid sugar coating (e.g., on lipooligosaccharide; LOS) using sialyltransferases that they express. LOS sialylation shields bacteria from complement and cationic peptides and also engages inhibitory siglec receptors on host immune cells to dampen inflammatory responses [50,51]. Unlike the molecular mimicry properties of Neu5Ac used by pathogenic bacteria such as *Neisseria gonorrhoeae,* which causes sexually transmitted gonorrhea infection, incorporating Leg5,7Ac_2_ instead of Neu5Ac to the LOS by *Neisseria gonorrhoeae* had been found to make the bacterium sensitive to complement and human serum [51,52]. Therefore, applying CMP-Leg5,7Ac_2_, which can be used by some human pathogens to decorate their LOS with Leg5,7Ac_2_, has been proposed as a novel therapeutic approach to address the global threat of multidrug-resistant bacteria [51,52,53]. Nevertheless, the host–pathogen interplay involving sialic acids and other nonulosonic acids with sialidases, siglecs, complement, cationic antimicrobial peptides, serum antibodies, and the complement system is complex and warrants further investigation.

To gain further insight into human IgG immune recognition of Leg5,7Ac_2_, binding of IVIG to Leg5,7Ac_2_-glycans on the glycan microarray was examined in the presence of selected competing glycans, including methyl α-*N*-glycolylneuraminoside (2-*O*-methyl-αNeu5Gc, or Gc2Me), glycopeptides of diverse *N*/*O*-glycans produced from mice sera digestion (mGP) [46], and the glycans that showed top preference of binding on the arrays including glycans ID-51, ID-502, ID-508 (Core1β; Leg5,7Ac_2_α3Core1β; and Leg5,7Ac_2_α6Core1β, respectively; Figure 3a). This analysis revealed that only αNeu5Gc (Gc2Me) did not affect IVIG binding to Leg5,7Ac_2_-glycans, while all other glycans inhibited its binding (Figure 3b). Particularly, while IVIG binding to all Leg5,7Ac_2_-glycans was inhibited by 50% with Core1β (ID-51) and by 80% with mGP, the competing Leg5,7Ac_2_α3/6Core1β-glycans (ID-502/508) completely abolished it (Figure 3b,c). This suggests that human IgG is enriched with antibodies that recognize diverse Leg5,7Ac_2_-glycans, in which the Leg5,7Ac_2_ moiety is critical for their recognition, although with some promiscuity with the variation in the underlying glycans including Core1/Lactose/LacNAc. Based on these inhibition studies together with previous structural studies of antibodies against sialyl-Tn [54] and sialyl Lewis a (sLe^a^) [55], it is plausible that the Leg5,7Ac_2_ moiety is buried within the binding pocket of these anti-Leg5,7Ac_2_ IgG antibodies.

## 3. Materials and Methods

### 3.1. Materials and Instruments

Chemicals were purchased and used without further purification. Nuclear magnetic resonance (NMR) spectra were recorded in the NMR facility of the University of California, Davis on 600 MHz and 800 MHz Bruker Avance III-NMR spectrometers and a 400 MHz Bruker Avance III HD Nanobay spectrometer (Bruker, Billerica, MA, USA). Chemical shifts are reported in parts per million (ppm) on the δ scale. High-resolution electrospray ionization (ESI) mass spectra were obtained using a Thermo Electron LTQ-Orbitrap Hybrid mass spectrometer (Thermo Fisher Scientific, Waltham, MA, USA) at the mass spectrometry facility at the University of California, Davis. Column chromatography was performed using a CombiFlash^®^ Rf 200i system (Teledyne ISCO, Lincoln, NE, USA) with either Redi*Sep* Rf silica columns or an ODS-SM (C18) column (51 g, 50 μm, 120 Å, Yamazen, Osaka, Japan) or manually using columns packed with silica gel 60 Å (230–400 mesh, Sorbent Technologies, Norcross, GA, USA). Thin layer chromatography (TLC) was performed on silica gel plates (Sorbent Technologies) using anisaldehyde sugar stain or 5% sulfuric acid in ethanol stain for detection. Gel filtration chromatography was performed with a column (100 cm × 2.5 cm) packed with Bio-Gel P-2 Fine resins (Bio-Rad, Hercules, CA, USA). Neu5GcaOMe (Gc2Me) was synthesized as reported previously [46]. *Pasteurella multocida* sialic acid aldolase (PmAldolase) [56], *Neisseria meningitidis* CMP-sialic acid synthetase (NmCSS) [57], *Pasteurella multocida* α2–3-sialyltransferase 1 (PmST1) [25], *Photobacterium damselae* α2–6-sialyltransferase (Pd2,6ST) [42], and Pd2,6ST_A200Y/S232Y were expressed and purified as described previously. PBSx10 was purchased from Hy-labs (Rehovot, Israel), ethanolamine from Fisher Scientific (Waltham, MA, USA), ovalbumin grade V from Sigma-Aldrich (St. Louis, MO, USA), SiaFindα2-6 from LectenzBio (Athens, GA, USA), anti-Neu5Gc IgY from BioLegend (San Diego, CA, USA), the lectins MAL-II, AAL, ConA, ECL, GSL-II, LcH, LEL, PNA, PSA, VVL, and WGA were purchased from Vector Laboratories (Newark, CA, USA), and ChromPure Human IgG whole molecule, Cy3-AffiniPure goat-anti-human IgG H+L, Cy3-sreptavidin, and Cy3-anti-chicken IgY from Jackson ImmunoResearch (West Grove, PA, USA). AlexaFlour-555-Hydraside from Invitrogen (Waltham, MA, USA). Pooled human IgG were IVIG-1 (Privigen from CSL Behring USA, King of Prussia, PA, USA) and IVIG-2 (OMRIgG-AM clinical remnants).

### 3.2. General Procedures for One-Pot Three-Enzyme (OP3E) Preparative-Scale Synthesis of Leg5,7diN_3_α2–6-Linked Nonulosonosides (***12***–***18***)

Acceptors [30] (GalNAcαProNHCbz **5**, LacβProNHCbz **6**, LacNAcβProNHCbz **7**, Galβ1–3GalNAcβProNHCbz **8**, Galβ1–3GalNAcαProNHCbz **9**, Galβ1–3GlcNAcβProNHCbz **10**, or Galβ1–3GlcNAcαProNHCbz **11**) (0.10–0.20 mmol), 6deoxyMan2,4diN_3_ [9] (**4**, 0.15–0.30 mmol, 1.5 eq.), sodium pyruvate (7.5 eq), and CTP (1.5 eq) were dissolved in water in a 50 mL centrifuge tube containing Tris-HCl buffer (100 mM, pH 8.5) and MgCl_2_ (20 mM). After adding sialic acid aldolase (1–3 mg), NmCSS (0.5–1.0 mg), and Pd2,6ST (1–4 mg, for acceptors **5**–**7**, **9**–**11**) or Pd2,6ST_A200Y/S232Y28 (2 mg, for acceptor **8**), water was added to bring the final concentration of acceptor to 10 mM. The reaction mixture was incubated at 30 °C for 24–36 h. The reaction progress was monitored using TLC (ethyl acetate:methanol:water = 6:1:1, by volume, was used as the developing solvent) and mass spectrometry. The reaction mixture was diluted with the same volume of ethanol and incubated at 4 °C for 30 min. The mixture was then centrifuged and concentrated, which was purified by an automated flash chromatograph using a C18 column (acetonitrile in water gradient was used as running solvents) to obtain the desired nonulosonosides.

*Leg5,7diN_3_α2–6GalNAcαProNHCbz* (**12**). A total of 110 mg, 99% yield, white amorphous solid. ^1^H NMR (800 MHz, D_2_O) δ 7.46–7.36 (m, 5H), 5.15–5.07 (m, 2H), 4.12 (dd, *J* = 11.0, 3.8 Hz, 1H), 4.02–3.81 (m, 7H), 3.78–3.62 (m, 3H), 3.58–3.52 (m, 2H), 3.46 (dt, *J* = 10.8, 5.9 Hz, 1H), 3.31–3.16 (m, 2H), 2.75 (dd, *J* = 12.5, 4.5 Hz, 1H), 2.01 (s, 3H), 1.97 (s, 3H), 1.94 (s, 3H), 1.80 (p, *J* = 6.4 Hz, 2H), 1.65 (t, *J* = 12.1 Hz, 1H), 1.15 (d, *J* = 6.3 Hz, 3H). ^13^C{^1^H} NMR (200 MHz, D_2_O) δ 174.4, 174.1, 157.8, 136.5, 128.6, 128.1, 127.6, 101.4, 97.1, 72.1, 69.4, 69.0, 67.8, 67.6, 66.6, 66.1, 65.7, 65.3, 63.8, 61.4, 59.3, 49.9, 40.2, 37.5, 28.9, 22.0, 19.2. HRMS (ESI) *m*/*z* calculated for C_28_H_39_N_8_O_13_^−^ [M − H]^−^ 695.2642, found 695.2611.

*Leg5,7diN_3_α2–6LacβProNHCbz* (**13**). A total of 57 mg, 75% yield, white amorphous solid. ^1^H NMR (800 MHz, D_2_O) δ 7.50 7.32 (m, 5H), 5.11 (s, 2H), 4.44 (d, *J* = 8.1 Hz, 1H), 4.40 (d, *J* = 7.9 Hz, 1H), 4.20–4.11 (m, 1H), 3.98–3.89 (m, 4H), 3.81–3.66 (m, 8H), 3.64 (dd, *J* = 10.0, 3.4 Hz, 1H), 3.61–3.50 (m, 6H), 3.46 (dd, *J* = 8.8, 2.4 Hz, 1H), 3.31 (t, *J* = 8.7 Hz, 1H), 3.29–3.19 (m, 2H), 2.71 (dd, *J* = 12.6, 4.8 Hz, 1H), 1.85–1.74 (m, 3H), 1.38 (d, *J* = 6.3 Hz, 3H). ^13^C{^1^H} NMR (200 MHz, D_2_O) δ 173.0, 158.4, 136.6, 128.8, 128.3, 127.7, 103.3, 102.0, 100.8, 79.9, 74.6, 74.6, 73.6, 72.6, 72.4, 72.0, 70.8, 69.4, 68.3, 67.7, 66.8, 66.5, 65.8, 63.5, 63.3, 60.3, 59.3, 39.9, 37.3, 28.8, 18.9. HRMS (ESI) *m*/*z* calculated for C_32_H_46_N_7_O_18_^−^ [M − H]^−^ 816.1905, found 816.1880.

*Leg5,7diN_3_α2–6LacNAcβProNHCbz* (**14**). A total of 26 mg, 70% yield, white amorphous solid. ^1^H NMR (600 MHz, D_2_O) δ 7.50–7.39 (m, 5H), 5.17–5.11 (m, 2H), 4.53 (d, *J* = 8.1 Hz, 1H), 4.42 (d, *J* = 7.9 Hz, 1H), 4.25–4.15 (m, 1H), 4.00–3.91 (m, 3H), 3.85–3.76 (m, 3H), 3.75 (s, 1H), 3.73–3.52 (m, 9H), 3.46–3.35 (m, 2H), 3.25–3.12 (m, 2H), 2.68 (dd, *J* = 12.6, 4.7 Hz, 1H), 2.06 (s, 3H), 1.83–1.71 (m, 3H), 1.40 (d, *J* = 6.3 Hz, 3H). ^13^C{^1^H} NMR (150 MHz, D_2_O) δ 174.2, 173.0, 158.3, 136.6, 128.8, 128.4, 127.6, 103.5, 100.8, 100.6, 80.8, 74.5, 73.6, 72.5, 72.5, 72.0, 70.8, 69.2, 68.3, 67.6, 66.8, 66.3, 65.5, 63.8, 63.4, 60.4, 59.3, 55.0, 39.9, 37.3, 28.7, 22.2, 18.9. HRMS (ESI) *m*/*z* calculated for C_34_H_49_N_8_O_18_^−^ [M − H]^−^ 857.3170, found 857.3141.

*Leg5,7diN_3_α2–6Galβ1–3GalNAcβProNHCbz* (**15**). A total of 73 mg, 93% yield, white amorphous solid. ^1^H NMR (600 MHz, D_2_O) δ 7.47–7.40 (m, 5H), 5.14–5.08 (m, 2H), 4.41 (d, *J* = 7.8 Hz, 1H), 4.39 (d, *J* = 7.8 Hz, 1H), 4.15–4.10 (m, 2H), 4.04–3.67 (m, 10H), 3.64–3.59 (m, 4H), 3.52–3.45 (m, 3H), 3.24–3.12 (m, 2H), 2.72 (dd, *J* = 12.6, 4.8 Hz, 1H), 2.00 (s, 3H), 1.85–1.64 (m, 3H), 1.38 (d, *J* = 6.0 Hz, 3H). ^13^C{^1^H} NMR (150 MHz, D_2_O) δ 174.71, 173.00, 158.31, 136.67, 128.78, 128.33, 127.66, 104.59, 101.43, 100.72, 79.74, 74.85, 73.19, 72.32, 72.01, 70.47, 69.33, 68.43, 68.06, 67.46, 66.76, 66.59, 65.73, 63.59, 63.50, 61.14, 59.47, 51.20, 39.97, 37.14, 28.73, 22.20, 18.88. HRMS (ESI) *m*/*z* calculated for C_34_H_49_N_8_O_18_^−^ [M − H]^−^ 857.3170, found 857.3174.

*Leg5,7diN_3_α2–6Galβ1–3GalNAcαProNHCbz* (**16**). A total of 38 mg, 77% yield, white amorphous solid. ^1^H NMR (400 MHz, D_2_O) δ 7.59–7.31 (m, 5H), 5.25–5.01 (m, 2H), 4.85 (d, *J* = 3.6 Hz, 1H), 4.45 (dd, *J* = 15.5, 7.7 Hz, 1H), 4.36–4.26 (m, 1H), 4.24–4.09 (m, 2H), 4.06–3.87 (m, 4H), 3.81–3.64 (m, 7H), 3.62–3.38 (m, 5H), 3.36–3.17 (m, 2H), 2.85–2.60 (m, 1H), 2.01 (s, 3H), 1.94–1.69 (m, 3H), 1.38 (d, *J* = 6.3 Hz, 3H). ^13^C{^1^H} NMR (100 MHz, D_2_O) δ 174.5, 158.4, 136.7, 128.8, 128.3, 127.5, 104.7, 104.5, 97.1, 77.8, 77.4, 75.9, 74.9, 74.7, 72.5, 72.1, 70.6, 69.4, 69.0, 68.5, 67.6, 66.7, 66.4, 65.4, 63.4, 61.2, 60.9, 59.3, 48.6, 39.2, 37.6, 28.5, 22.0, 18.9. HRMS (ESI) *m*/*z* calculated for C_34_H_49_N_8_O_18_^−^ [M − H]^−^ 857.3170, found 857.3134.

*Leg5,7diN_3_α2–6Galβ1–3GlcNAcβProNHCbz* (**17**). A total of 62 mg, 81% yield, white amorphous solid. ^1^H NMR (800 MHz, D_2_O) δ 7.51–7.37 (m, 5H), 5.10 (d, *J* = 5.0 Hz, 2H), 4.48 (d, *J* = 8.5 Hz, 1H), 4.35 (d, *J* = 7.8 Hz, 1H), 4.15 (dq, *J* = 8.8, 6.3 Hz, 1H), 3.97 (dd, *J* = 12.3, 2.3 Hz, 1H), 3.94–3.88 (m, 3H), 3.81–3.73 (m, 4H), 3.73–3.65 (m, 2H), 3.64–3.58 (m, 2H), 3.56 (dd, *J* = 10.4, 4.0 Hz, 1H), 3.54–3.42 (m, 5H), 3.29–3.08 (m, 2H), 2.69 (dd, *J* = 12.6, 4.8 Hz, 1H), 1.99 (s, 3H), 1.82–1.68 (m, 3H), 1.37 (d, *J* = 6.3 Hz, 3H). ^13^C{^1^H} NMR (200 MHz, D_2_O) δ 174.6, 173.0, 158.3, 136.6, 128.8, 128.3, 127.6, 103.9, 100.9, 100.5, 84.3, 75.3, 73.5, 72.5, 72.0, 70.5, 69.3, 69.1, 68.4, 67.6, 66.8, 66.5, 65.7, 63.7, 63.5, 61.0, 59.3, 54.2, 39.9, 37.2, 28.7, 22.1, 18.9. HRMS (ESI) *m*/*z* calculated for C_34_H_49_N_8_O_18_^−^ [M − H]^−^ 857.3170, found 857.3139.

*Leg5,7diN_3_α2–6Galβ1–3GlcNAcαProNHCbz* (**18**). A total of 49 mg, 99% yield, white amorphous solid. ^1^H NMR (400 MHz, D_2_O) δ 7.54–7.26 (m, 5H), 5.12 (s, 2H), 4.34 (d, *J* = 7.8 Hz, 1H), 4.22–4.10 (m, 1H), 4.06 (dd, *J* = 10.5, 3.6 Hz, 1H), 3.99–3.65 (m, 11H), 3.63–3.36 (m, 7H), 3.34–3.14 (m, 2H), 2.71 (dd, *J* = 12.7, 4.6 Hz, 1H), 2.01 (s, 3H), 1.90–1.64 (m, 3H), 1.37 (d, *J* = 6.2 Hz, 3H). ^13^C{^1^H} NMR (100 MHz, D_2_O) δ 174.4, 173.1, 158.3, 136.6, 128.8, 128.3, 127.6, 103.8, 100.9, 96.8, 82.1, 73.5, 72.5, 71.9, 71.5, 70.6, 69.3, 68.9, 68.3, 66.7, 66.4, 65.8, 65.0, 63.7, 63.2, 60.7, 59.3, 52.2, 40.0, 37.5, 28.6, 22.0, 18.9. HRMS (ESI) *m*/*z* calculated for C_34_H_49_N_8_O_18_^−^ [M − H]^−^ 857.3170, found 857.3138.

### 3.3. General Procedures for One-Pot Three-Enzyme (OP3E) Preparative-Scale Synthesis of Leg5,7diN_3_α2–3-Linked Nonulosonosides (***19***–***24***)

Acceptors [30] (LacβProNHCbz **6**, LacNAcβProNHCbz **7**, Galβ1–3GalNAcβProNHCbz **8**, Galβ1–3GalNAcαProNHCbz **9**, Galβ1–3GlcNAcβProNHCbz **10**, or Galβ1–3GlcNAcαProNHCbz **11**) (0.10–0.20 mmol), 6deoxyMan2,4diN_3_ [9] (**4**, 0.15–0.30 mmol, 1.5 eq.), sodium pyruvate (7.5 eq), and CTP (1.5 eq) were dissolved in water in a 50 mL centrifuge tube containing Tris-HCl buffer (100 mM, pH 8.5) and MgCl_2_ (20 mM). After adding sialic acid aldolase (1–3 mg), NmCSS (0.5–1.0 mg), and PmST1_M144D (1–4 mg), water was added to bring the final concentration of the acceptor to 10 mM. The reaction mixture was incubated at 30 °C for 24–36 h. The reaction progress was monitored using TLC (ethyl acetate:methanol:water = 6:1:1, by volume, was used as the developing solvent) and mass spectrometry. The reaction mixture was diluted with the same volume of ethanol and incubated at 4 °C for 30 min. The mixture was then centrifuged and concentrated, which was purified by automated flash chromatography using a C18 column (CH_3_CN in H_2_O gradient was used as running solvents) to obtain the desired nonulosonosides.

Leg5,7diN_3_α2–3LacβProNHCbz (**19**). A total of 56 mg, 74% yield, white amorphous solid. ^1^H NMR (400 MHz, D_2_O) δ 7.61–7.33 (m, 5H), 5.11 (s, 2H), 4.50 (d, *J* = 7.8 Hz, 1H), 4.42 (d, *J* = 8.1 Hz, 1H), 4.25–4.09 (m, 2H), 4.07–3.83 (m, 5H), 3.70–3.60 (m, 4H), 3.59–3.48 (m, 5H), 3.47–3.36 (m, 2H), 3.32–3.19 (m, 2H), 2.82–2.62 (m, 2H), 1.86–1.70 (m, 3H), 1.46–1.31 (m, 3H). ^13^C{^1^H} NMR (100 MHz, D_2_O) δ 171.8, 158.4, 136.6, 128.8, 128.3, 127.6, 102.6, 102.2, 102.1, 78.4, 75.7, 75.1, 74.7, 74.4, 72.8, 72.2, 72.0, 69.3, 67.9, 67.7, 66.7, 65.8, 65.3, 63.5, 61.7, 61.0, 60.3, 59.3, 39.3, 37.3, 28.9, 18.8. HRMS (ESI) *m*/*z* calculated for C_32_H_46_N_7_O_18_^−^ [M − H]^−^ 816.1905, found 816.1870.

*Leg5,7diN_3_α2–3LacNAcβProNHCbz* (**20**). A total of 27 mg, 71% yield, white amorphous solid. ^1^H NMR (600 MHz, D_2_O) δ 7.50–7.39 (m, 5H), 5.17–5.08 (m, 2H), 4.54 (d, *J* = 7.9 Hz, 1H), 4.47 (d, *J* = 8.3 Hz, 1H), 4.22–4.14 (m, 1H), 4.04 (dd, *J* = 9.9, 3.0 Hz, 1H), 3.99 (dd, *J* = 12.4, 2.3 Hz, 1H), 3.95–3.88 (m, 2H), 3.84 (dd, *J* = 12.5, 5.1 Hz, 1H), 3.78–3.66 (m, 8H), 3.63–3.52 (m, 4H), 3.45 (dd, *J* = 8.6, 2.2 Hz, 1H), 3.23–3.11 (m, 2H), 2.73 (dd, *J* = 12.7, 4.5 Hz, 1H), 2.02 (s, 3H), 1.91 (t, *J* = 12.2 Hz, 1H), 1.84–1.70 (m, 2H), 1.40 (t, *J* = 6.3 Hz, 3H). ^13^C{^1^H} NMR (150 MHz, D_2_O) δ 174.5, 172.8, 158.3, 136.6, 128.8, 128.4, 127.6, 102.6, 101.1, 101.0, 78.5, 75.7, 75.1, 74.7, 72.4, 72.2, 69.4, 69.3, 67.9, 67.6, 66.6, 65.3, 63.4, 61.0, 60.1, 59.3, 55.0, 38.8, 37.2, 28.8, 22.1, 18.8. HRMS (ESI) *m*/*z* calculated for C_34_H_49_N_8_O_18_^−^ [M − H]^−^ 857.3170, found 857.3137.

*Leg5,7diN_3_α2–3Galβ1–3GalNAcβProNHCbz* (**21**). A total of 23 mg, 96% yield, white amorphous solid. ^1^H NMR (400 MHz, D_2_O) δ 7.55–7.31 (m, 5H), 5.23–5.07 (m, 2H), 4.54–4.36 (m, 2H), 4.21–4.11 (m, 1H), 4.04–3.97 (m, 1H), 3.95–3.88 (m, 2H), 3.86–3.50 (m, 12H), 3.47–3.28 (m, 2H), 3.26–3.10 (m, 1H), 2.92 (d, *J* = 14.7 Hz, 2H), 2.74 (d, *J* = 12.6 Hz, 1H), 2.01 (s, 3H), 1.93–1.72 (m, 3H), 1.39 (d, *J* = 6.2 Hz, 3H). ^13^C{^1^H} NMR (200 MHz, D_2_O) δ 174.69, 173.39, 158.34, 136.64, 128.77, 128.31, 127.60, 104.82, 104.59, 101.34, 80.20, 75.83, 74.76, 74.71, 72.09, 69.44, 68.87, 67.84, 67.58, 67.47, 66.46, 65.37, 63.30, 60.94, 60.90, 59.28, 51.10, 48.83, 39.18, 37.20, 29.56, 22.20, 18.84. HRMS (ESI) *m*/*z* calculated for C_34_H_49_N_8_O_18_^−^ [M − H]^−^ 857.3170, found 857.3135.

*Leg5,7diN_3_α2–3Galβ1–3GalNAcαProNHCbz* (**22**). A total of 36 mg, 77% yield, white amorphous solid. ^1^H NMR (400 MHz, D_2_O) δ 7.50–7.37 (m, 5H), 5.19–5.07 (m, 2H), 4.85 (d, *J* = 3.7 Hz, 1H), 4.47 (d, *J* = 7.9 Hz, 1H), 4.30 (dd, *J* = 11.1, 3.6 Hz, 1H), 4.25–4.11 (m, 2H), 4.06–3.86 (m, 4H), 3.83–3.64 (m, 7H), 3.62–3.37 (m, 5H), 3.27 (q, *J* = 6.9 Hz, 2H), 2.74 (dd, *J* = 12.7, 4.7 Hz, 1H), 2.01 (s, 3H), 1.93–1.74 (m, 3H), 1.39 (d, *J* = 6.3 Hz, 3H). ^13^C{^1^H} NMR (100 MHz, D_2_O) δ 174.5, 173.4, 158.3, 136.7, 128.8, 128.3, 127.5, 104.5, 100.4, 97.1, 77.8, 75.9, 74.7, 72.1, 70.6, 69.4, 69.0, 68.5, 67.6, 66.7, 66.4, 65.4, 65.1, 63.4, 61.2, 60.9, 48.6, 39.2, 37.6, 28.5, 22.0, 18.9. HRMS (ESI) *m*/*z* calculated for C_34_H_49_N_8_O_18_^−^ [M − H]^−^ 857.3170, found 857.3141.

*Leg5,7diN_3_α2–3Galβ1–3GlcNAcβProNHCbz* (**23**). A total of 60 mg, 80% yield, white amorphous solid. ^1^H NMR (800 MHz, D_2_O) δ 7.50–7.35 (m, 5H), 5.13–5.06 (m, 2H), 4.49 (d, *J* = 8.5 Hz, 1H), 4.45 (d, *J* = 7.8 Hz, 1H), 4.19–4.08 (m, 1H), 3.99 (dd, *J* = 9.8, 3.2 Hz, 1H), 3.95–3.87 (m, 3H), 3.85–3.57 (m, 9H), 3.56–3.48 (m, 3H), 3.48–3.39 (m, 2H), 3.25–3.07 (m, 2H), 2.73 (dd, *J* = 12.6, 4.7 Hz, 1H), 2.01 (s, 3H), 1.85 (t, *J* = 12.3 Hz, 1H), 1.74 (p, *J* = 6.3 Hz, 2H), 1.37 (d, *J* = 6.4 Hz, 3H). ^13^C{^1^H} NMR (200 MHz, D_2_O) δ 174.5, 173.3, 158.3, 136.6, 128.8, 128.3, 127.6, 103.5, 103.5, 100.8, 82.7, 75.9, 75.3, 75.1, 72.0, 69.4, 68.9, 68.7, 67.6, 67.5, 66.7, 66.5, 65.5, 63.3, 60.9, 60.7, 59.3, 54.4, 39.2, 37.2, 28.7, 22.2, 18.9. HRMS (ESI) *m*/*z* calculated for C_34_H_49_N_8_O_18_^−^ [M − H]^−^ 857.3170, found 857.3142.

*Leg5,7diN_3_α2–3Galβ1–3GlcNAcαProNHCbz* (**24**). A total of 47 mg, 96% yield, white amorphous solid. ^1^H NMR (400 MHz, D_2_O) δ 7.53–7.32 (m, 5H), 5.13 (s, 2H), 4.48–4.39 (m, 1H), 4.21–4.10 (m, 1H), 4.07 (dd, *J* = 10.6, 3.5 Hz, 1H), 3.99 (dd, *J* = 9.7, 3.0 Hz, 1H), 3.93–3.65 (m, 11H), 3.63–3.37 (m, 6H), 3.35–3.14 (m, 2H), 2.74 (dd, *J* = 12.6, 4.6 Hz, 1H), 2.02 (s, 3H), 1.92–1.72 (m, 3H), 1.38 (d, *J* = 6.2 Hz, 3H). ^13^C{^1^H} NMR (100 MHz, D_2_O) δ 174.3, 173.2, 158.4, 136.7, 128.8, 128.3, 127.5, 103.4, 100.6, 96.9, 81.0, 75.9, 75.0, 72.1, 71.5, 69.4, 69.1, 68.7, 67.5, 66.7, 66.5, 65.4, 65.0, 63.3, 60.9, 60.5, 59.3, 52.3, 39.2, 37.4, 28.5, 22.0, 18.9. HRMS (ESI) *m*/*z* calculated for C_34_H_49_N_8_O_18_^−^ [M − H]^−^ 857.3170, found 857.3132.

### 3.4. General Procedures for Synthesizing Leg5,7Ac_2_-Glycosides (25–37) by Converting the Azido Groups in Leg5,7diN_3_-Glycosides (***12***–***24***) to NHAc Groups

To a saturated sodium bicarbonate solution in water in a round bottom flask (100 mL), an azido-containing glycoside selected from **12**–**24** (10–40 mg) was added followed by drop-wise addition of 12–24 equivalents of thioacetic acid under argon at room temperature and this was stirred at 70 °C for 20 h. After completion of the reaction, the solvent was removed under vacuum. The mixture was passed through a Bio-Gel P-2 gel filtration column (water was used as an eluent). Then, the product-containing fractions were concentrated and further purified by silica gel chromatography using a mixed solvent (ethyl acetate: methanol: water = 10:1:0.1, by volume) as an eluent, followed by C18 purification (CH_3_CN in H_2_O gradient was used as running solvents) to obtain pure products.

Leg5,7Ac_2_α2–6GalNAcαProNHCbz (**25**). A total of 30 mg, 65% yield, white amorphous solid. ^1^H NMR (400 MHz, D_2_O) δ 7.54–7.28 (m, 5H), 5.29–5.03 (m, 2H), 4.20–4.08 (m, 1H), 4.05–3.87 (m, 6H), 3.87–3.64 (m, 5H), 3.62–3.39 (m, 3H), 3.35–3.12 (m, 2H), 2.76 (dd, *J* = 12.6, 4.3 Hz, 1H), 2.01 (s, 3H), 1.97 (s, 3H), 1.95 (s, 3H), 1.81 (p, *J* = 6.3 Hz, 2H), 1.66 (t, *J* = 12.0 Hz, 1H), 1.15 (d, *J* = 6.2 Hz, 3H). ^13^C{^1^H} NMR (100 MHz, D_2_O) δ 174.5, 173.9, 173.7, 173.6, 158.3, 136.6, 128.8, 128.3, 127.6, 97.0, 71.5, 69.5, 68.6, 67.6, 67.2, 66.7, 65.5, 64.2, 54.1, 54.0, 52.1, 49.9, 40.4, 37.6, 28.5, 22.1, 22.0, 21.9, 18.1. HRMS (ESI) *m*/*z* calculated for C_32_H_47_N_4_O_15_^−^ [M − H]^−^ 727.3043, found 727.3006.

*Leg5,7Ac_2_α2–6LacβProNHCbz* (**26**). A total of 23 mg, 78% yield, white amorphous solid. ^1^H NMR (400 MHz, D_2_O) δ 7.59–7.27 (m, 5H), 5.12 (s, 2H), 4.55–4.34 (m, 2H), 4.10–3.50 (m, 18H), 3.42–3.19 (m, 3H), 2.74 (dd, *J* = 12.4, 4.7 Hz, 1H), 2.01 (s, 3H), 1.95 (s, 3H), 1.89–1.67 (m, 3H), 1.16 (d, *J* = 6.3 Hz, 3H). ^13^C{^1^H} NMR (100 MHz, D_2_O) δ 173.9, 173.7, 173.4, 158.4, 136.6, 128.8, 128.3, 127.6, 103.2, 102.0, 100.3, 79.5, 74.6, 73.6, 72.8, 72.5, 72.4, 71.6, 70.7, 68.8, 68.4, 67.7, 67.1, 66.8, 63.4, 61.0, 60.2, 54.0, 52.1, 40.1, 37.3, 28.9, 22.1, 22.1, 18.1. HRMS (ESI) *m*/*z* calculated for C_36_H_54_N_3_O_20_^−^ [M − H]^−^ 848.3306, found 848.3253.

*Leg5,7Ac_2_α2–6LacNAcβProNHCbz* (**27**). A total of 9 mg, 68% yield, white amorphous solid. ^1^H NMR (800 MHz, D_2_O) δ 7.69–7.29 (m, 5H), 5.19–5.03 (m, 2H), 4.55–4.41 (m, 2H), 4.06–3.76 (m, 9H), 3.75–3.50 (m, 10H), 3.26–3.08 (m, 2H), 2.76–2.65 (m, 1H), 2.03 (s, 3H), 1.97 (s, 3H), 1.92 (s, 3H), 1.85–1.66 (m, 3H), 1.17–1.11 (m, 3H). ^13^C{^1^H} NMR (200 MHz, D_2_O) δ 174.5, 173.7, 173.6, 173.4, 158.3, 136.6, 128.8, 128.3, 127.6, 103.3, 100.8, 100.3, 80.0, 74.5, 73.5, 72.4, 72.3, 71.7, 70.7, 68.7, 68.3, 67.6, 67.1, 66.7, 63.1, 60.2, 55.1, 54.0, 52.0, 40.1, 37.2, 28.7, 22.2, 22.1, 22.0, 18.0. HRMS (ESI) *m*/*z* calculated for C_38_H_57_N_4_O_20_^−^ [M − H]^−^ 889.3572, found 889.3540.

*Leg5,7Ac_2_α2–6Galβ1–3GalNAcβProNHCbz* (**28**). A total of 25 mg, 90% yield, white amorphous solid. ^1^H NMR (800 MHz, D_2_O) δ 7.45–7.38 (m, 5H), 5.13–5.08 (m, 2H), 4.43 (d, *J* = 8.0 Hz, 1H), 4.42 (d, *J* = 8.0 Hz, 1H), 4.19 (d, *J* = 3.2 Hz, 1H), 4.02–3.54 (m, 16H), 3.52–3.49 (m, 2H), 3.22–3.12 (m, 2H), 2.74 (dd, *J* = 12.8, 4.8 Hz, 1H), 2.00 (s, 6H), 1.94 (s, 3H), 1.76–1.72 (m, 2H), 1.62 (t, *J* = 4.0 Hz, 1H), 1.74 (s, 2H), 1.62 (t, *J* = 12.1 Hz, 1H), 1.13 (d, *J* = 6.4 Hz, 3H). ^13^C{^1^H} NMR (200 MHz, D_2_O) δ 173.91, 173.69, 173.34, 158.34, 136.64, 128.76, 128.31, 127.61, 104.28, 101.43, 100.34, 80.36, 74.68, 73.18, 72.40, 71.63, 70.41, 68.65, 68.50, 67.73, 67.50, 67.01, 66.76, 63.64, 62.83, 61.21, 53.95, 52.10, 51.13, 40.32, 37.19, 28.72, 22.16, 22.12, 22.05, 18.01. HRMS (ESI) *m*/*z* calculated for C_38_H_57_N_4_O_20_^−^ [M − H]^−^ 889.3572, found 889.3580.

*Leg5,7Ac_2_α2–6Galβ1–3GalNAcαProNHCbz* (**29**). A total of 6 mg, 68% yield, white amorphous solid. ^1^H NMR (800 MHz, D_2_O) δ 7.57–7.24 (m, 5H), 5.11 (q, *J* = 12.8 Hz, 2H), 4.42 (d, *J =* 7.7 Hz, 1H), 4.34–4.26 (m, 1H), 4.19–4.12 (m, 1H), 4.06–3.98 (m, 2H), 3.97–3.62 (m, 12H), 3.60–3.43 (m, 4H), 3.32–3.16 (m, 2H), 2.77 (dd, *J* = 12.4, 4.6 Hz, 1H), 1.94 (s, 6H), 1.92 (s, 3H), 1.84–1.75 (m, 2H), 1.71 (t, *J* = 12.1 Hz, 1H), 1.11 (d, *J* = 6.4 Hz, 3H). ^13^C{^1^H} NMR (200 MHz, D_2_O) δ 174.4, 173.9, 173.8, 173.7, 158.4, 136.7, 128.7, 128.3, 127.4, 104.4, 99.4, 97.1, 77.0, 75.6, 74.8, 71.6, 70.6, 68.9, 68.6, 67.3, 66.8, 66.7, 61.2, 60.9, 53.9, 51.9, 48.7, 40.2, 37.4, 28.4, 22.1, 22.0, 21.9, 18.0. HRMS (ESI) *m*/*z* calculated for C_38_H_57_N_4_O_20_^−^ [M − H]^−^ 889.3572, found 889.3516.

*Leg5,7Ac_2_α2–6Galβ1–3GlcNAcβProNHCbz* (**30**). A total of 21 mg, 78% yield, white amorphous solid. ^1^H NMR (800 MHz, D_2_O) δ 7.48–7.35 (m, 5H), 5.16–5.06 (m, 2H), 4.50 (d, *J* = 8.5 Hz, 1H), 4.38 (d, *J* = 7.9 Hz, 1H), 4.06–3.96 (m, 3H), 3.95–3.87 (m, 3H), 3.86–3.77 (m, 4H), 3.75–3.68 (m, 2H), 3.66–3.60 (m, 2H), 3.59–3.52 (m, 4H), 3.51–3.46 (m, 1H), 3.25–3.10 (m, 2H), 2.72 (dd, *J* = 12.5, 4.7 Hz, 1H), 2.01 (s, 3H), 1.99 (s, 3H), 1.95 (s, 3H), 1.80–1.70 (m, 3H), 1.15 (d, *J* = 6.3 Hz, 3H).^13^C{^1^H} NMR (200 MHz, D_2_O) δ 174.6, 173.9, 173.6, 173.4, 158.3, 136.6, 128.8, 128.3, 127.6, 104.0, 100.9, 100.5, 84.4, 75.6, 73.5, 72.5, 71.6, 70.5, 69.0, 68.7, 68.3, 67.6, 67.0, 66.8, 63.2, 60.9, 54.2, 54.0, 52.0, 40.1, 37.2, 28.7, 22.2, 22.1, 18.1. HRMS (ESI) *m*/*z* calculated for C_38_H_57_N_4_O_20_^−^ [M − H]^−^ 889.3572, found 889.3534.

*Leg5,7Ac_2_α2–6Galβ1–3GlcNAcαProNHCbz* (**31**). A total of 17 mg, 71% yield, white amorphous solid. ^1^H NMR (800 MHz, D_2_O) δ 7.52–7.31 (m, 5H), 5.22–4.99 (m, 2H), 4.33 (d, *J* = 7.9 Hz, 1H), 4.13–4.05 (m, 1H), 4.04–3.78 (m, 9H), 3.76–3.67 (m, 4H), 3.62–3.43 (m, 6H), 3.33–3.17 (m, 2H), 2.70 (dd, *J* = 12.4, 4.7 Hz, 1H), 1.98 (s, 6H), 1.93 (s, 3H), 1.87–1.77 (m, 2H), 1.77–1.70 (m, 1H), 1.13 (d, *J* = 6.3 Hz, 3H). ^13^C{^1^H} NMR (200 MHz, D_2_O) δ 174.4, 173.9, 173.6, 173.5, 158.4, 136.6, 128.8, 128.3, 127.4, 103.9, 100.1, 96.8, 82.5, 73.4, 72.5, 71.6, 71.6, 70.5, 68.8, 68.7, 68.2, 67.0, 66.7, 65.0, 63.0, 60.6, 54.0, 52.1, 52.0, 40.1, 37.4, 28.5, 22.1, 21.9, 18.0. HRMS (ESI) *m*/*z* calculated for C_38_H_57_N_4_O_20_^−^ [M − H]^−^ 889.3572, found 889.3536.

*Leg5,7Ac_2_α2–3LacβProNHCbz* (**32**). A total of 7 mg, 68% yield, white amorphous solid. ^1^H NMR (800 MHz, D_2_O) δ 7.49–7.35 (m, 5H), 5.10 (s, 2H), 4.48 (d, *J* = 7.9 Hz, 1H), 4.42 (d, *J* = 8.0 Hz, 1H), 4.11–4.05 (m, 1H), 4.00–3.89 (m, 3H), 3.87–3.64 (m, 10H), 3.64–3.59 (m, 2H), 3.59–3.51 (m, 3H), 3.31–3.17 (m, 2H), 2.77 (dd, *J* = 12.4, 4.6 Hz, 1H), 1.97 (d, *J* = 2.0 Hz, 3H), 1.93 (s, 3H), 1.79 (p, *J* = 6.7 Hz, 2H), 1.73 (t, *J* = 12.1 Hz, 1H), 1.14 (d, *J* = 11.5 Hz, 3H). ^13^C{^1^H} NMR (200 MHz, D_2_O) δ 173.9, 173.7, 169.0, 158.4, 136.6, 128.7, 128.3, 127.6, 102.6, 102.0, 95.1, 78.3, 75.3, 75.1, 74.7, 74.3, 72.7, 71.8, 69.3, 68.7, 67.7, 67.2, 66.8, 66.8, 61.0, 60.1, 58.6, 53.9, 52.0, 40.1, 37.3, 28.8, 22.1, 21.9, 18.0. HRMS (ESI) *m*/*z* calculated for C_36_H_54_N_3_O_20_^−^ [M − H]^−^ 848.3306, found 848.3271.

*Leg5,7Ac_2_α2–3LacNAcβProNHCbz* (**33**). A total of 9 mg, 67% yield, white amorphous solid. ^1^H NMR (800 MHz, D_2_O) δ 7.41 (dq, *J* = 17.8, 9.0, 7.9 Hz, 5H), 5.15–5.05 (m, 2H), 4.53–4.48 (m, 1H), 4.44 (dd, *J* = 10.0, 8.0 Hz, 1H), 4.10 (dd, *J* = 9.9, 3.1 Hz, 1H), 4.00–3.92 (m, 3H), 3.90–3.78 (m, 4H), 3.74–3.52 (m, 11H), 3.26–3.07 (m, 2H), 2.77 (dd, *J* = 12.4, 4.6 Hz, 1H), 2.00 (s, 3H), 1.97 (d, *J* = 4.6 Hz, 3H), 1.92 (d, *J* = 2.9 Hz, 3H), 1.76–1.68 (m, 3H), 1.17–1.11 (m, 3H). ^13^C{^1^H} NMR (200 MHz, D_2_O) δ 174.5, 174.0, 173.7, 173.7, 158.3, 136.6, 128.8, 128.3, 127.6, 103.3, 102.5, 99.5, 78.4, 75.3, 75.1, 74.7, 72.4, 71.8, 69.4, 68.7, 67.6, 67.2, 66.9, 62.4, 61.0, 60.0, 55.0, 53.9, 52.0, 40.0, 37.2, 28.7, 22.1, 22.1, 21.9, 18.0. HRMS (ESI) *m*/*z* calculated for C_38_H_57_N_4_O_20_^−^ [M − H]^−^ 889.3572, found 889.3540.

*Leg5,7Ac_2_α2–3Galβ1–3GalNAcβProNHCbz* (**34**). A total of 5 mg, 67% yield, white amorphous solid. ^1^H NMR (800 MHz, D_2_O) δ 7.46–7.36 (m, 5H), 5.18–5.06 (m, 2H), 4.46–4.36 (m, 2H), 4.12 (d, *J* = 3.2 Hz, 1H), 4.06–3.98 (m, 2H), 3.96–3.86 (m, 3H), 3.82–3.53 (m, 13H), 3.28–3.09 (m, 1H), 2.90 (d, *J* = 46.7 Hz, 1H), 2.77 (dd, *J* = 12.4, 4.6 Hz, 1H), 1.96 (s, 3H), 1.92 (s, 6H), 1.85–1.65 (m, 3H), 1.11 (d, *J* = 6.1 Hz, 3H). ^13^C{^1^H} NMR (200 MHz, D_2_O) δ 173.9, 173.8, 173.7, 172.9, 158.3, 136.7, 128.7, 128.3, 127.5, 104.7, 101.2, 99.3, 75.6, 74.9, 74.8, 72.0, 71.6, 68.8, 68.6, 68.0, 67.4, 67.3, 66.8, 61.0, 60.9, 60.8, 53.9, 51.9, 51.3, 40.2, 37.1, 28.6, 22.2, 22.1, 21.9, 18.0. HRMS (ESI) *m*/*z* calculated for C_38_H_57_N_4_O_20_^−^ [M − H]^−^ 889.3572, found 889.3536.

*Leg5,7Ac_2_α2–3Galβ1–3GalNAcαProNHCbz* (**35**). A total of 13 mg, 69% yield, white amorphous solid. ^1^H NMR (800 MHz, D_2_O) δ 7.42 (dd, *J* = 27.7, 7.4 Hz, 5H), 5.17–5.07 (m, 2H), 4.83–4.82 (m, 1H), 4.47 (d, *J* = 7.9 Hz, 1H), 4.32 (dd, *J* = 11.1, 3.8 Hz, 1H), 4.18 (d, *J* = 3.2 Hz, 1H), 4.06–4.00 (m, 2H), 3.98–3.93 (m, 2H), 3.90 (d, *J* = 3.2 Hz, 1H), 3.86–3.79 (m, 2H), 3.77–3.68 (m, 6H), 3.60–3.52 (m, 3H), 3.51–3.43 (m, 1H), 3.33–3.16 (m, 2H), 2.79 (dd, *J* = 12.4, 4.5 Hz, 1H), 1.98 (s, 3H), 1.98 (s, 3H), 1.94 (s, 3H), 1.80 (h, *J* = 6.6 Hz, 2H), 1.73 (t, *J* = 12.1 Hz, 1H), 1.13 (d, *J* = 6.3 Hz, 3H). ^13^C{^1^H} NMR (200 MHz, D_2_O) δ 174.4, 173.9, 173.7, 158.4, 136.7, 128.8, 128.3, 127.4, 104.4, 99.6, 97.1, 77.0, 75.7, 74.8, 71.7, 71.7, 70.6, 68.9, 68.7, 68.7, 67.3, 66.8, 66.7, 65.0, 61.2, 60.9, 53.9, 51.9, 48.7, 40.2, 37.5, 28.4, 22.1, 22.0, 21.9, 18.1. HRMS (ESI) *m*/*z* calculated for C_38_H_57_N_4_O_20_^−^ [M − H]^−^ 889.3572, found 889.3537.

*Leg5,7Ac_2_α2–3Galβ1–3GlcNAcβProNHCbz* (**36**). A total of 15 mg, 75% yield, white amorphous solid. ^1^H NMR (400 MHz, D_2_O) δ 7.54–7.34 (m, 5H), 5.18–5.05 (m, 2H), 4.47 (dd, *J* = 20.6, 8.1 Hz, 2H), 4.07 (dd, *J* = 9.9, 3.0 Hz, 1H), 3.99–3.42 (m, 17H), 3.21 (q, *J* = 7.4 Hz, 3H), 2.80 (dd, *J* = 12.4, 4.5 Hz, 1H), 2.05–1.96 (m, 6H), 1.95 (s, 3H), 1.85–1.63 (m, 3H), 1.13 (d, *J* = 6.2 Hz, 3H). ^13^C{^1^H} NMR (100 MHz, D_2_O) δ 174.3, 173.9, 173.7, 158.3, 136.6, 128.8, 128.3, 127.5, 103.6, 100.9, 100.8, 82.0, 75.7, 75.4, 75.2, 71.6, 68.8, 68.6, 68.6, 67.7, 67.4, 66.8, 61.1, 60.7, 54.5, 53.9, 51.9, 46.7, 40.2, 37.3, 28.7, 22.1, 22.1, 21.9, 18.1. HRMS (ESI) *m*/*z* calculated for C_38_H_57_N_4_O_20_^−^ [M − H]^−^ 889.3572, found 889.3532.

*Leg5,7Ac_2_α2–3Galβ1–3GlcNAcαProNHCbz* (**37**). A total of 11 mg, 72% yield, white amorphous solid. ^1^H NMR (800 MHz, D_2_O) δ 7.52–7.31 (m, 5H), 5.25–5.01 (m, 2H), 4.76 (d, *J* = 3.6 Hz, 1H), 4.45 (d, *J* = 8.0 Hz, 1H), 4.12–3.99 (m, 2H), 3.97–3.64 (m, 12H), 3.63–3.49 (m, 4H), 3.48–3.40 (m, 1H), 3.33–3.14 (m, 2H), 2.77 (dd, *J* = 12.4, 4.5 Hz, 1H), 1.97 (s, 3H), 1.95 (s, 3H), 1.92 (s, 3H), 1.84–1.74 (m, 2H), 1.71 (t, *J* = 12.1 Hz, 1H), 1.11 (d, *J* = 6.4 Hz, 3H). ^13^C{^1^H} NMR (200 MHz, D_2_O) δ 174.2, 173.9, 173.7, 158.4, 136.7, 128.7, 128.2, 127.4, 103.3, 99.4, 97.0, 79.8, 75.7, 75.0, 71.6, 71.6, 68.9, 68.6, 68.5, 67.3, 66.8, 66.7, 64.9, 61.0, 60.4, 53.9, 52.5, 51.9, 40.2, 37.3, 28.4, 22.1, 21.9, 21.9, 18.0. HRMS (ESI) *m*/*z* calculated for C_38_H_57_N_4_O_20_^−^ [M − H]^−^ 889.3572, found 889.3537.

### 3.5. General Procedures for Converting Cbz-Tagged Glycans to Glycosyl Propylamines

To a stirring solution of a Cbz-tagged glycoside selected from **25**–**37** (5–10 mg) in water-methanol solution (3 mL, 1:2 by volume), a catalytic amount of 10% palladium on charcoal was added to a 50 mL round bottom flask. The mixture was stirred under a hydrogen environment for 2–5 h. The solution was passed through a syringe filter. Methanol was removed from the filtrate by blowing air inside a fume hood. The residue was lyophilized and used directly for microarray assays.

### 3.6. Glycan Microarray Fabrication

Nano-printing of glycans microarray were fabricated as previously described [45,58]. Briefly, arrays (Version VLA1) were fabricated with NanoPrint LM-60 Microarray Printer (Arrayit) on epoxide-derivatized slides (PolyAn 2D) with 16 sub-array blocks on each slide, with each sub-array containing 340 spots arraigned in a 20 × 17 matrix. Glycoconjugates were distributed into 384-well source plates using 4 replicate wells per sample and 8 μL per well. Each glycoconjugate was diluted into 100 μM in an optimized printing buffer (300 mM phosphate buffer, pH 8.4). To monitor printing, ChromPure Human IgG whole molecule (at 20 ng/μL in PBS pH 7.4 + 10% glycerol) and AlexaFlour-555-Hydraside (at 2 ng/μL in 178 mM phosphate buffer, pH 5.5) were used for each sub-array. The arrays were printed with four 946MP3 pins (5 μm tip, 0.25 μL sample channel, ~100 μm spot diameter; Arrayit). The humidity level in the arraying chamber was maintained at about 70% during printing. Printed slides were left on the arrayer deck overnight, allowing humidity to drop to ambient levels (40–45%). Next, slides were packed, vacuum-sealed, and stored at room temperature (RT) until used.

### 3.7. Glycan Microarray Binding Assays

Slides were developed and analyzed as previously described [58] with some modifications. Slides were rehydrated with dH_2_O and incubated for 30 min in a staining dish with 50 °C pre-warmed ethanolamine (0.05 M) in Tris-HCl (0.1 M, pH 9.0), then washed with 50 °C pre-warmed dH_2_O. Slides were centrifuged at 200× *g* for five min then fitted with a ProPlate™ Multi-Array 16-well slide module (Grace Bio-lab) to divide into the sub-arrays (blocks). Slides were washed with PBST (0.1% Tween 20), aspirated, and blocked with 200 μL/sub-array of blocking buffer (PBS pH 7.3 + 1% *w*/*v* ovalbumin) for 1 h at RT with gentle shaking. Next, the blocking solution was aspirated and a 100 μL/sub-array of detection lectins/antibodies diluted in blocking buffer (lectins binding buffer also included divalent cations, as detailed in the ESI glycan microarray data file in Supplementary Materials) were incubated with gentle shaking for 2 h at RT.

For sialidase pretreatment, before blocking, sub-arrays were incubated with either PBS (without sialidase), 10 mU/100 μL *Arthrobacter ureafaciens* sialidase (AUS, EY Laboratories), or 10 mU/100 μL *Clostridium perfringens* neuraminidase (NCP, Rosch, Sigma, St. Louis, MO, USA), for 2 h at 37 °C with gentle shaking. Then, slides were washed twice with PBST (0.1% Tween 20), aspirated, and blocked with 200 μL/sub-array of blocking buffer (PBS pH 7.3 + 1% *w*/*v* ovalbumin) at RT for 1 h with gentle shaking.

For inhibition assays, each IVIG was pre-incubated for 2 h on ice with inhibition solution containing blocking buffer with glycans (2 mM ID#43, ID#51; or 1 mM ID#502, ID#508), glycopeptides (GP at 0.45 mM; produced from mice sera digested with Pronase) [59], 4 mM 2-*O*-methyl-α-Neu5Ac (Ac2Me), or 4 mM 2-*O*-methyl-α-Neu5Gc (Gc2Me) and only then applied to slides for 2 h. Slides were washed 5 times with PBST.

Binding was detected with Cy3-labeled secondary detection reagent diluted in PBS at 200 μL/sub-array at RT for 1 h with gentle shaking (0.4 ng/μL Cy3 AffiniPure goat-anti-human IgG (H + L), 0.375 ng/μL Cy3-anti-chicken IgY, 1.2 ng/μL Cy3-sterptavidin). Slides were washed 4 times with PBST, then with PBS (without Tween-20) for 10 min followed by removal from ProPlate™ Multi-Array slide module and immediately dipping in a staining dish with dH_2_O for 10 min with shaking. Slides were then centrifuged at 200× *g* for 5 min and the dry slides were immediately scanned.

### 3.8. Array Slide Processing

Processed slides were scanned and analyzed as described [45,58] at 10 μm resolution with a Genepix 4000B microarray scanner (Molecular Devices, San Jose, CA, USA) using 350 gain. Image analysis was carried out with Genepix Pro 7.0 analysis software (Molecular Devices). Spots were defined as circular features with a variable radius as determined by the Genepix scanning software. Local background subtraction was performed. RFU from each spot was calculated.

### 3.9. Statistics

Statistical analyses were performed using GraphPad Prism 10.2.0 and described in context in the Figure legends.

## 4. Conclusions

We demonstrate here that the chemoenzymatic synthon strategy using 6deoxyMan2,4diN_3_ (**4**) as the starting material in OP3E sialylation systems is highly efficient in constructing a comprehensive library of α2–3- and α2–6-linked Leg5,7Ac_2_-glycosides via the enzymatic formation of Leg5,7diN_3_, CMP-Leg5,7diN_3_, and Leg5,7diN_3_-glycosides intermediates, followed by chemical conversion of the azido to the acetamido groups. The hydrophobic UV-detectable Cbz group in the sialyltransferase acceptors facilitated product purification from the OP3E systems. Removal of the Cbz group in the ProNHCbz-tagged Leg5,7Ac_2_-glycosides produced ProNH_2_-tagged Leg5,7Ac_2_-glycosides ready for immobilization on the activated glass slide surface. Glycan microarray studies using the Leg5,7Ac_2_-glycosides and their structurally related sialoside pairs revealed diverse human IgG antibodies that selectively recognize the α-linked bacterial nonulosonic acid Leg5,7Ac_2_. Such broad-spectrum recognition by human IgG antibodies pooled from thousands of donors (IVIG) supports previous encounters of Leg5,7Ac_2_-expressing pathogens by the general human population. Overall, these resources and analytical tools are important tools for further in-depth investigation of Leg5,7Ac_2_-involved bacterial physiology, virulence, pathogenicity, and host–pathogen interactions.

## Figures and Tables

**Table 1:** Structures of the OP3E sialyltransferase acceptors and yields of Leg5,7diN_3_-glycosides (**12**–**24**) and Leg5,7Ac_2_-glycosides (**25**–**37**) products.

Acceptors (**5**–**11**) 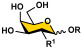 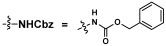	OP3E Products Leg5,7diN_3_-glycosides (**12**–**24**)		Leg5,7Ac_2_-glycosides (**25**–**37**)	
	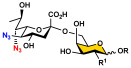 **12**–**18** (70–99%)	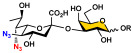 **19**–**24** (71–96%)	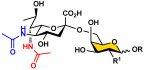 **25**–**31** (65–90%)	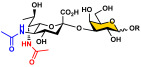 **32**–**37** (67–75%)
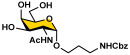 GalNAcαProNHCbz (**5**) R = αProNHCbz, R^1^ = NHAc	**12** (99%)	-	**25** (65%)	-
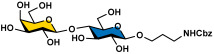 LacβProNHCbz (**6**) R = β4GlcβProNHCbz, R^1^ = OH	**13** (75%)	**19** (74%)	**26** (78%)	**32** (68%)
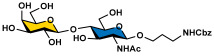 LacNAcβProNHCbz (**7**) R = β4GlcNAcβProNHCbz, R^1^ = OH	**14** (70%)	**20** (71%)	**27** (68%)	**33** (67%)
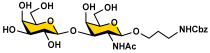 Galβ3GalNAcβProNHCbz (**8**) R = β3GalNAcβProNHCbz, R^1^ = OH	**15** (93%)	**21** (96%)	**28** (90%)	**34** (67%)
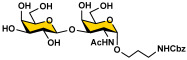 Galβ3GalNAcαProNHCbz (**9**) R = β3GalNAcαProNHCbz, R^1^ = OH	**16** (77%)	**22** (77%)	**29** (68%)	**35** (69%)
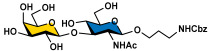 Galβ3GlcNAcβProNHCbz (**10**) R = β3GlcNAcβProNHCbz, R^1^ = OH	**17** (81%)	**23** (80%)	**30** (78%)	**36** (75%)
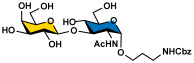 Galβ3GlcNAcαProNHCbz (**11**) R = β3GlcNAcαProNHCbz, R^1^ = OH	**18** (99%)	**24** (96%)	**31** (71%)	**37** (72%)

## Data Availability

The original contributions presented in the study are included in the article/Supplementary Materials, further inquiries can be directed to the corresponding authors.

## Supplementary Materials

The following supporting information can be downloaded at: https://www.mdpi.com/article/10.3390/molecules29163980/s1: the ^1^H and ^13^C NMR spectra of products **12**–**37** (PDF) and the glycan microarray data file (EXCEL).
