# Supplementary material for: Broad-Spectrum Legionaminic Acid-Specific Antibodies in Pooled Human IgGs Revealed by Glycan Microarrays with Chemoenzymatically Synthesized Nonulosonosides
Source: Molecules. 2024 Aug 22;29(16):3980. doi: 10.3390/molecules29163980 (PMC11356810; doi:10.3390/molecules29163980)

Supporting Information

Broad-Spectrum Legionaminic Acid-Specific Antibodies in Pooled Human IgGs Revealed by Glycan Microarrays with Chemoenzymatically Synthesized Nonulosonosides

Anoopjit Singh Kooner [1], Hai Yu [1], Shani Leviatan Ben-Arye [2], Vered Padler-Karavani [2,*] and Xi Chen [1,*]

[1] Department of Chemistry, University of California, Davis, CA 95616, USA; akooner@ucdavis.edu (A.S.K.); hyu@ucdavis.edu (H.Y.)
[2] Department of Cell Research and Immunology, The Shmunis School of Biomedicine and Cancer Research, The George S. Wise Faculty of Life Sciences, Tel Aviv University, Tel Aviv 69978, Israel; leviata@tauex.tau.ac.il

* Correspondence: vkaravani@tauex.tau.ac.il (V.P.-K.); xiichen@ucdavis.edu (X.C.); Tel.: +972-3-640-6737 (V.P.-K.); +1-530-754-6037 (X.C.)

Table of content

^{1}H and ^{13}C NMR spectra of **12–37**……………………………………………………………S2–S27

800 MHz ^{1}H and 200 MHz $^{13}C\{^{1}H\}$ NMR spectra of Leg5,7diN$_3$α2–6GalNAcαProNHCbz (**12**) in D_2O.

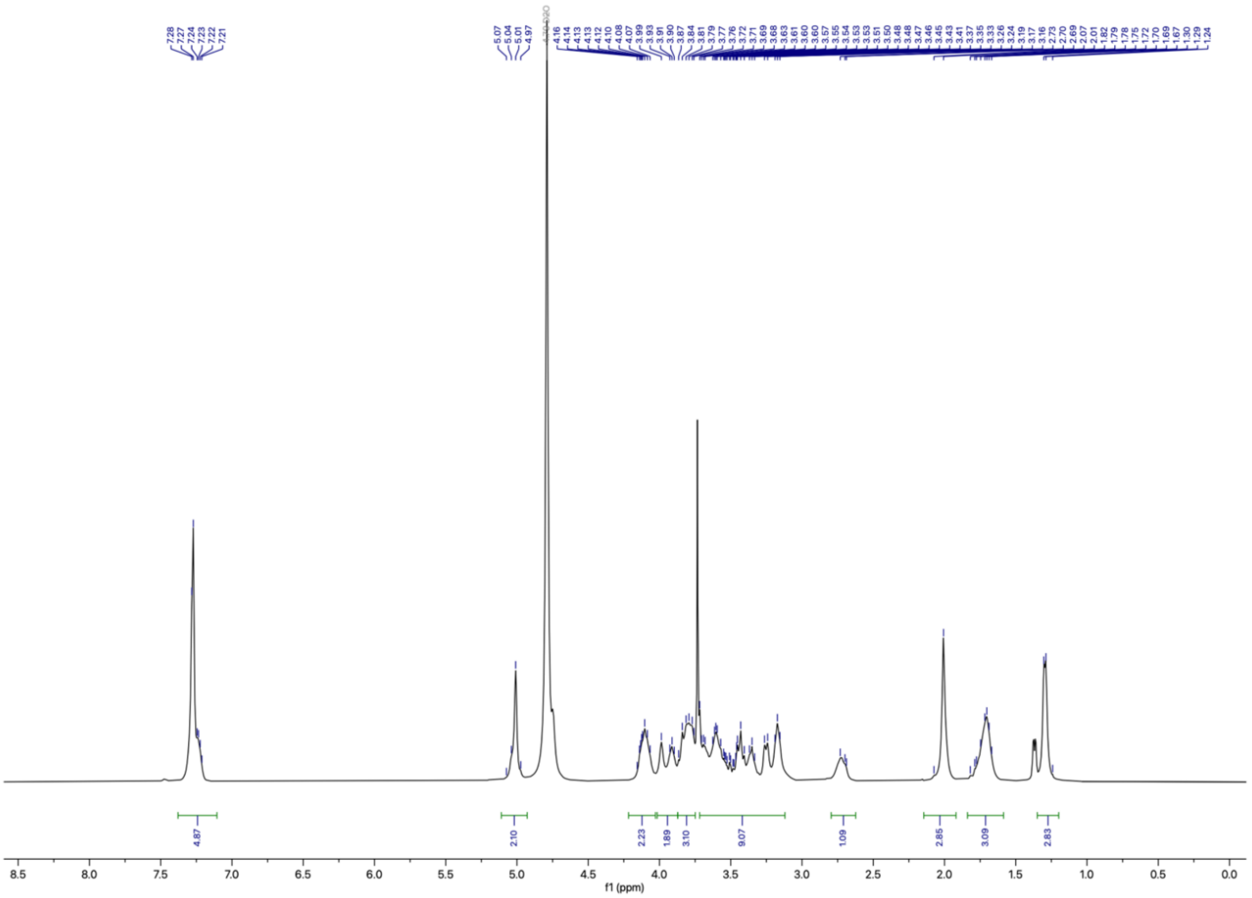

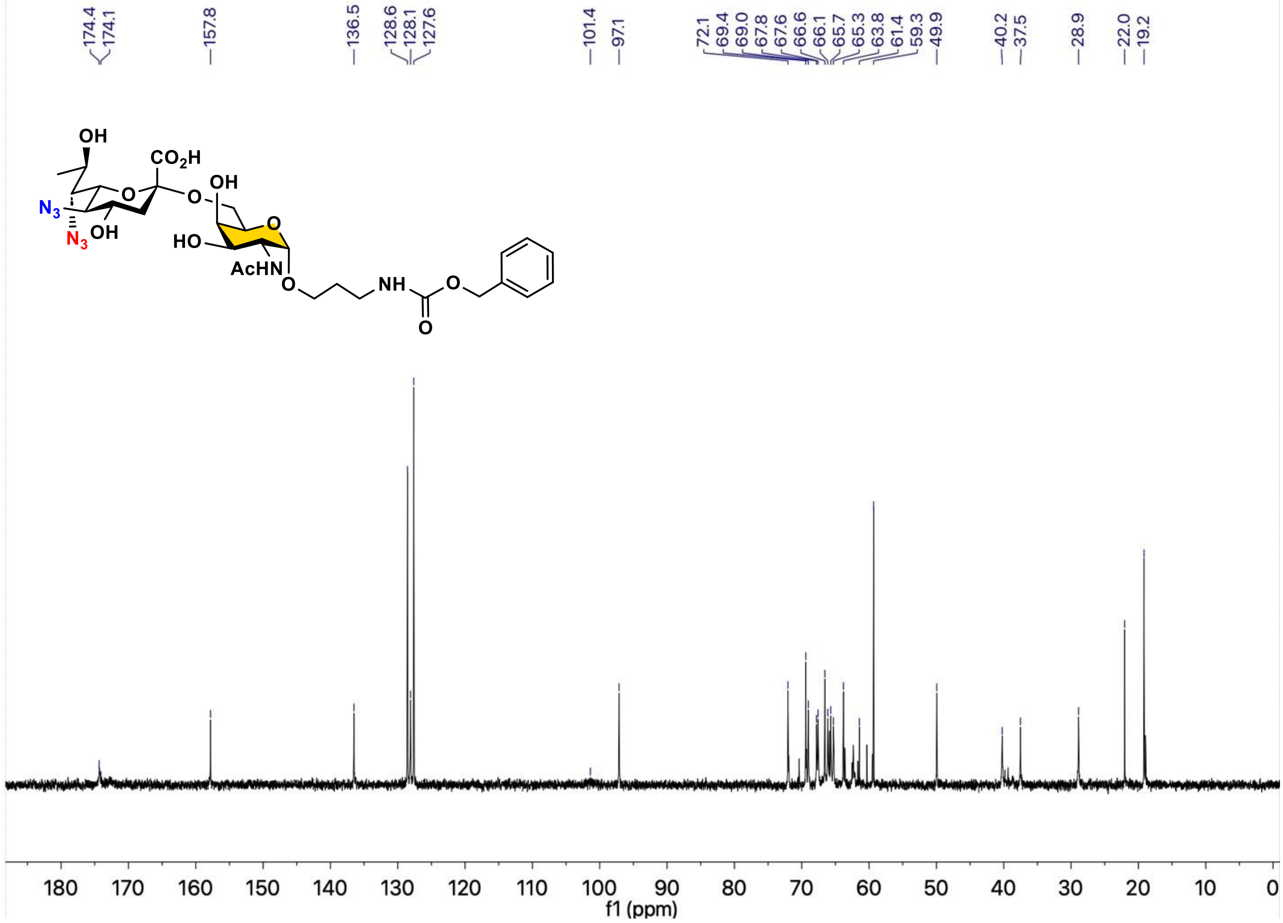

800 MHz ^{1}H and 200 MHz $^{13}C\{^{1}H\}$ NMR spectra of Leg5,7diN$_3$α2–6LacβProNHCbz (**13**) in D_2O.

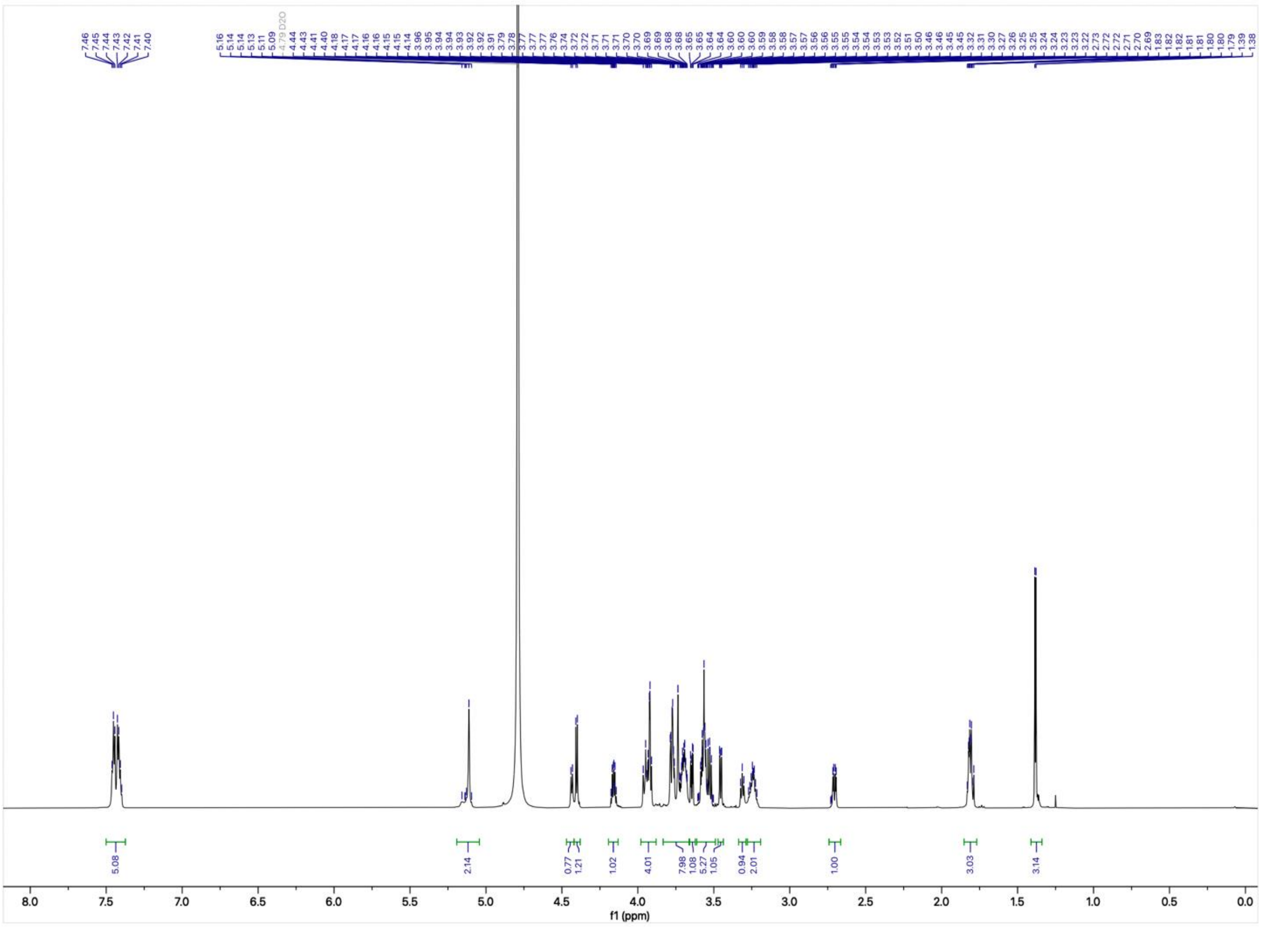

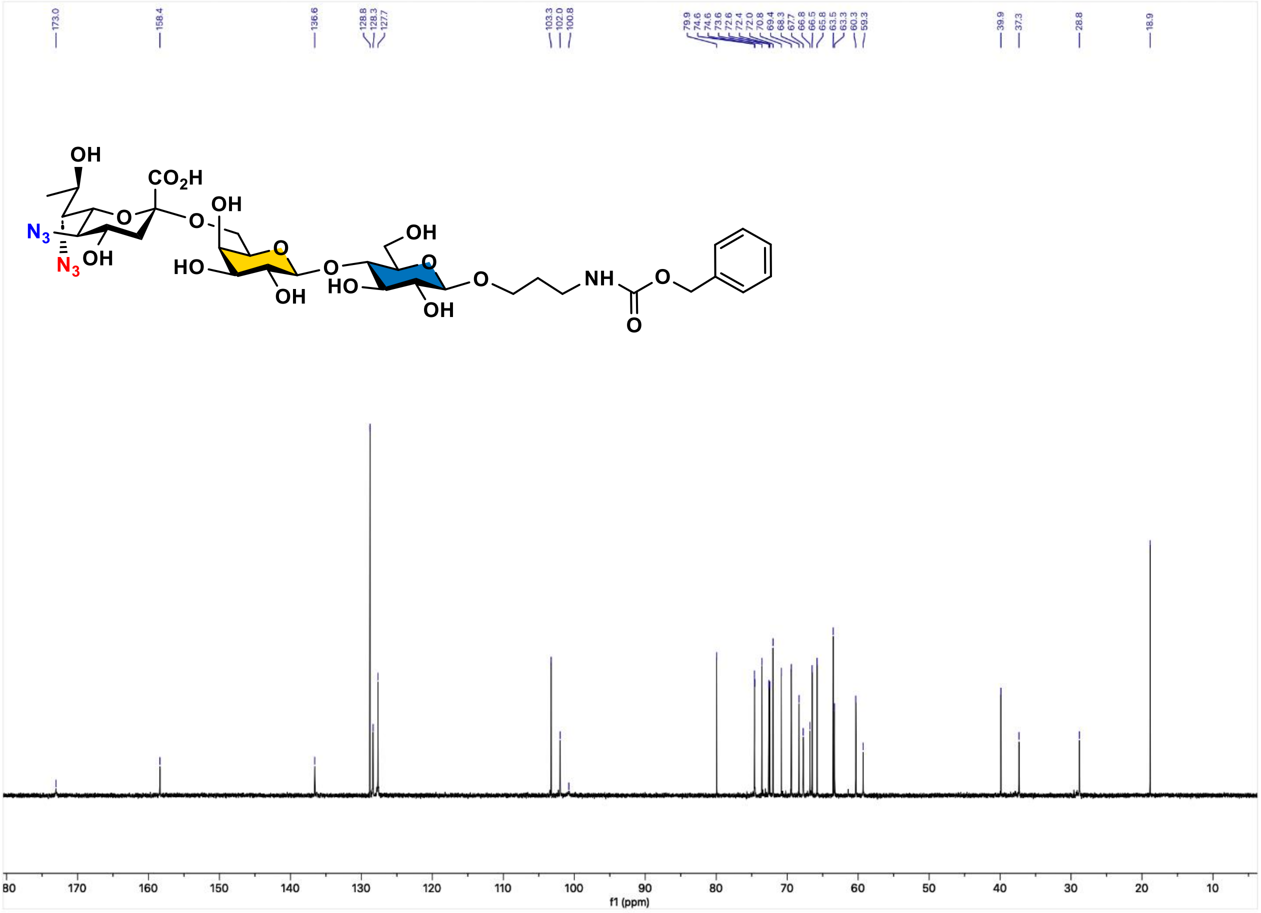

600 MHz ^{1}H and 150 MHz $^{13}C\{^{1}H\}$ NMR spectra of Leg5,7diN$_3$α2−6LacNAcβProNHCbz (**14**) in D_2O.

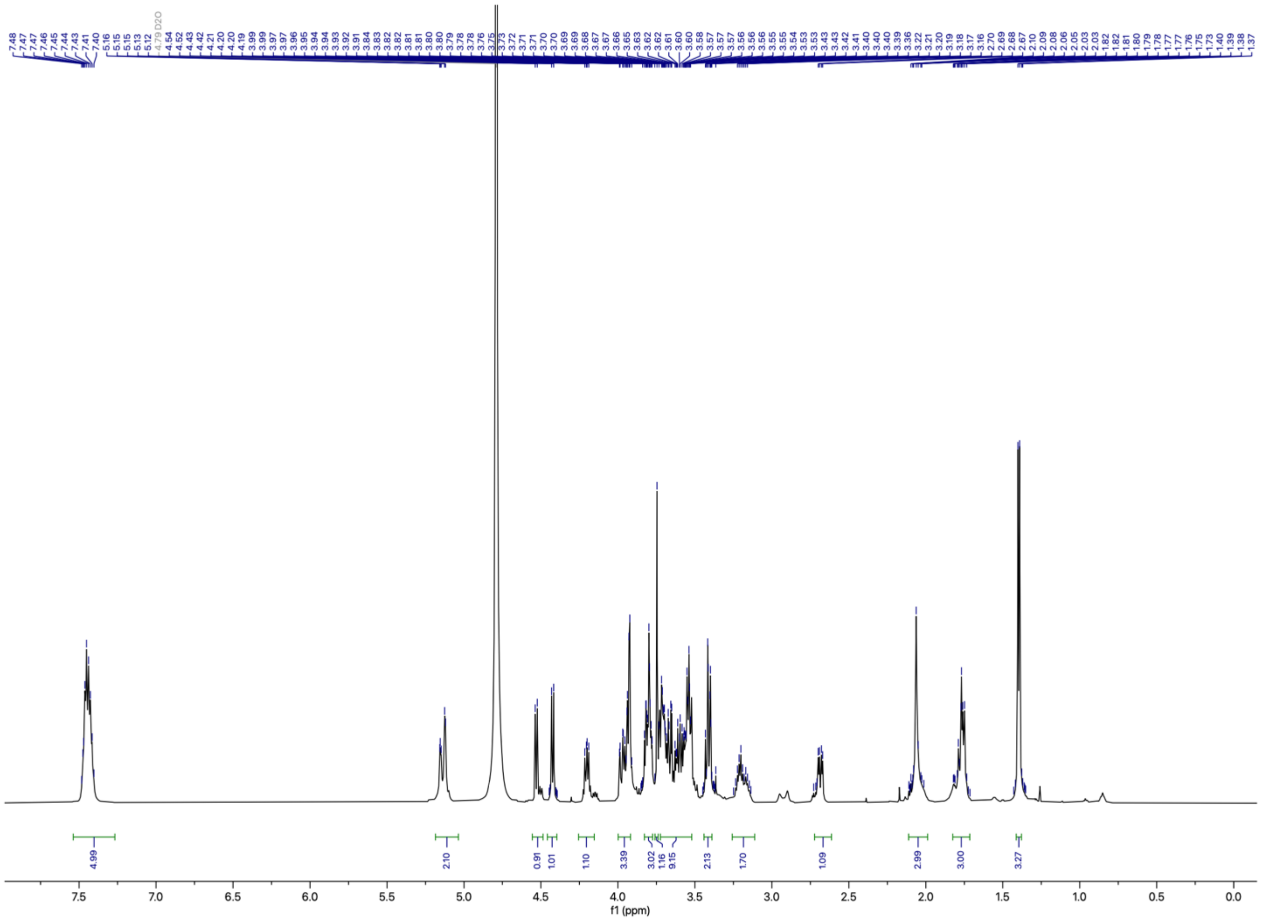

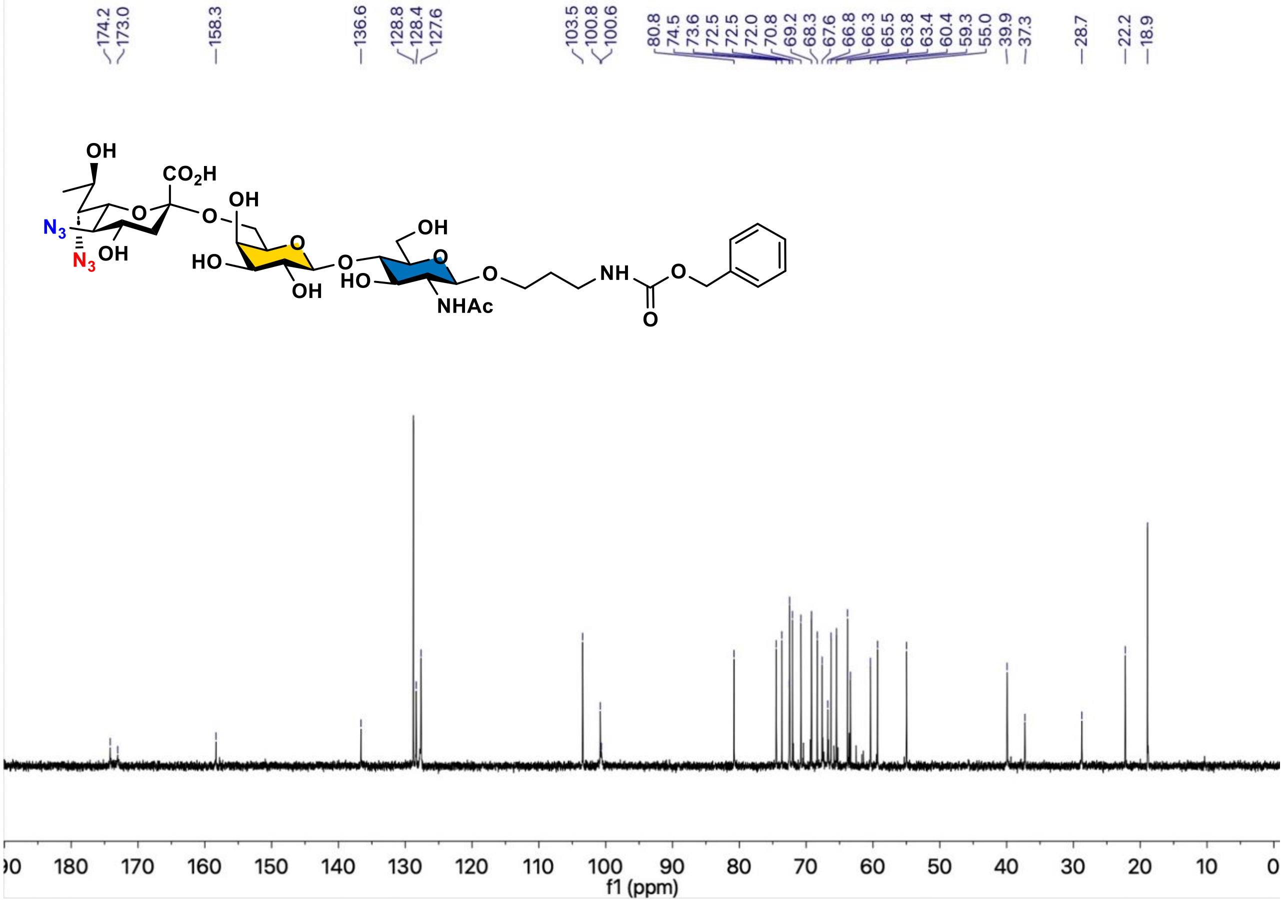

600 MHz ¹H and 150 MHz ¹³C{¹H} NMR spectra of Leg5,7diN$_3$α2–6Galβ1−3GalNAcβProNHCbz (**15**) in D_2O.

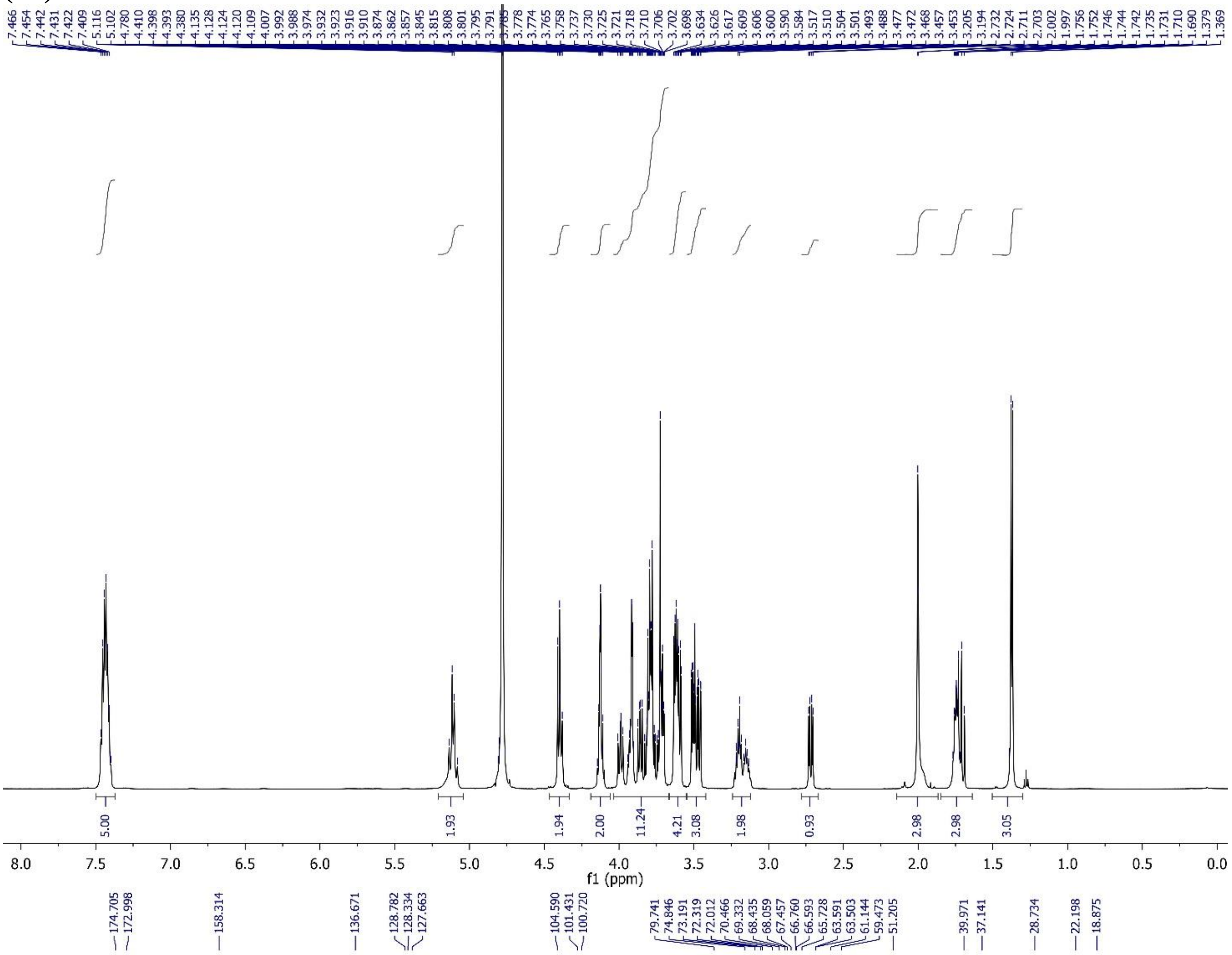

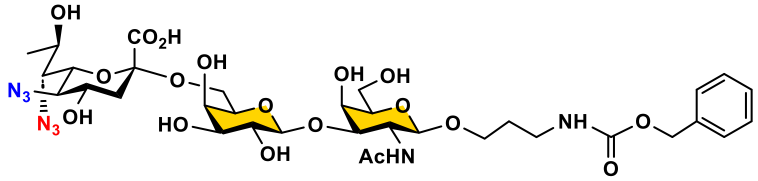

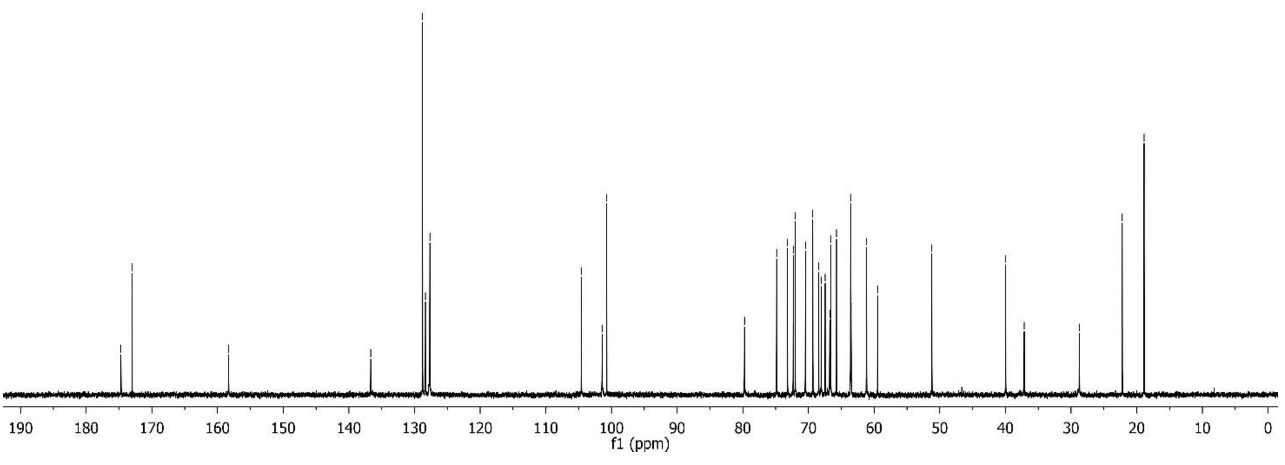

400 MHz ^{1}H and 100 MHz $^{13}C\{^{1}H\}$ NMR spectra of Leg5,7diN$_3$α2–6Galβ1–3GalNAcαProNHCbz (**16**) in D_2O.

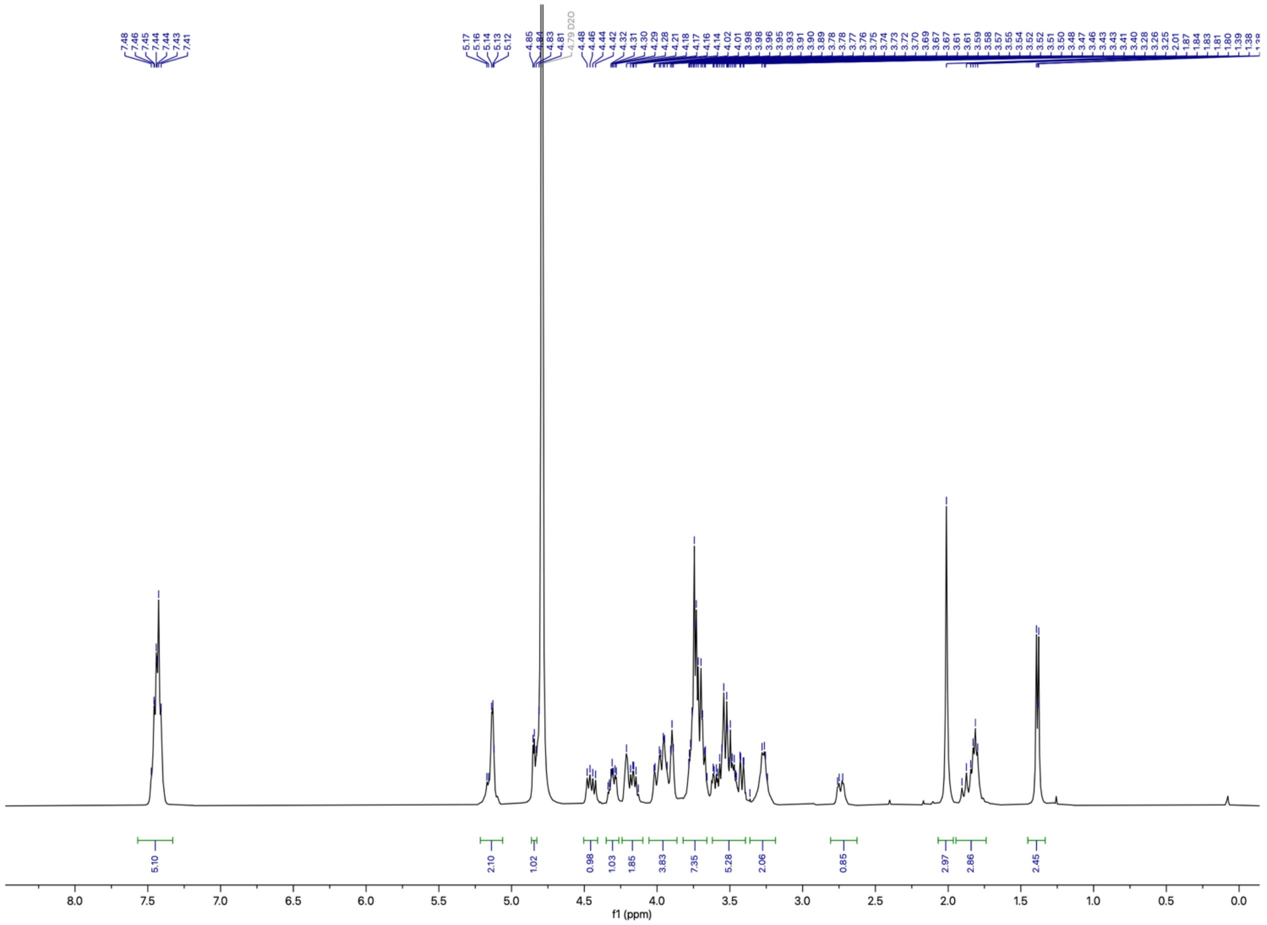

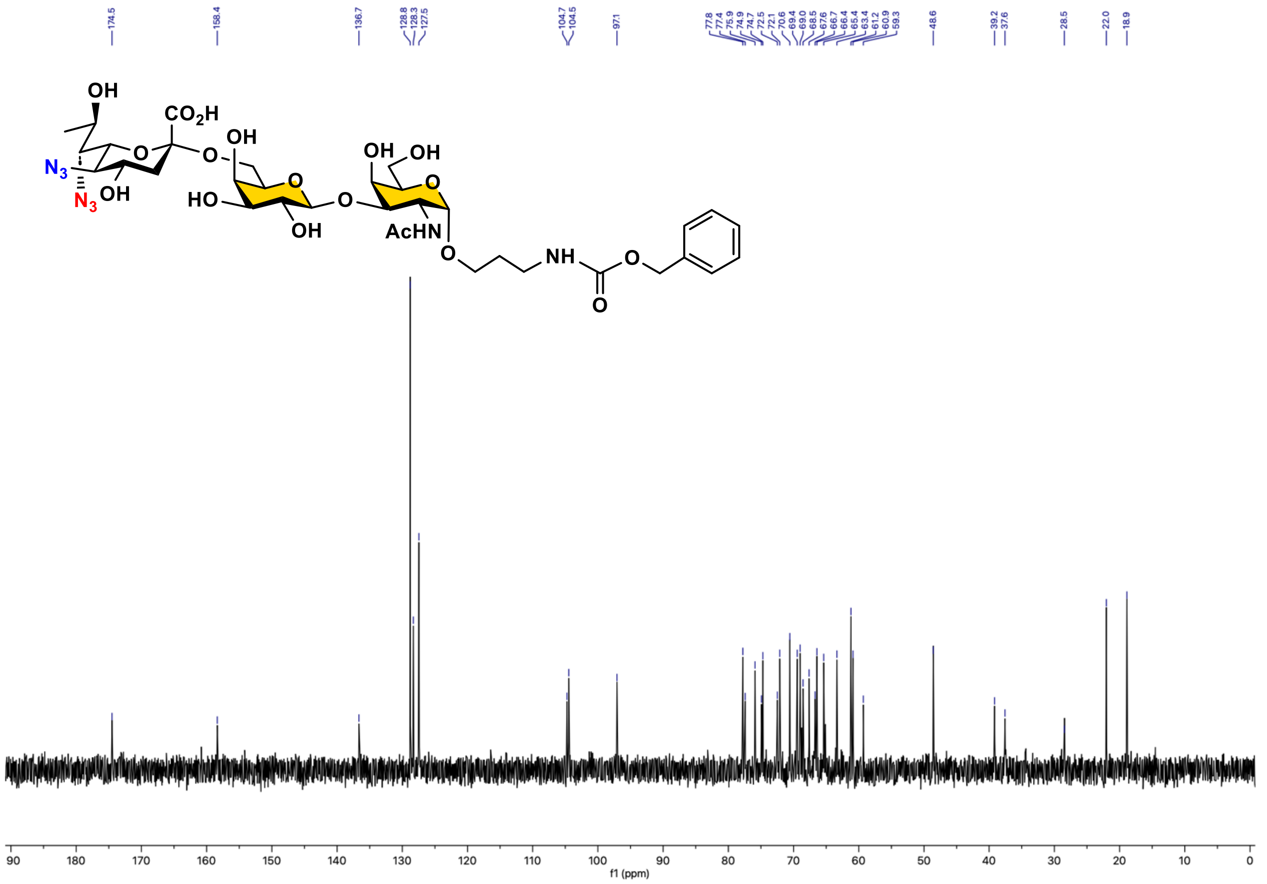

800 MHz ^{1}H and 200 MHz $^{13}C\{^{1}H\}$ NMR spectra of Leg5,7diN$_3$α2–6Galβ1–3GlcNAcβProNHCbz (**17**) in D_2O.

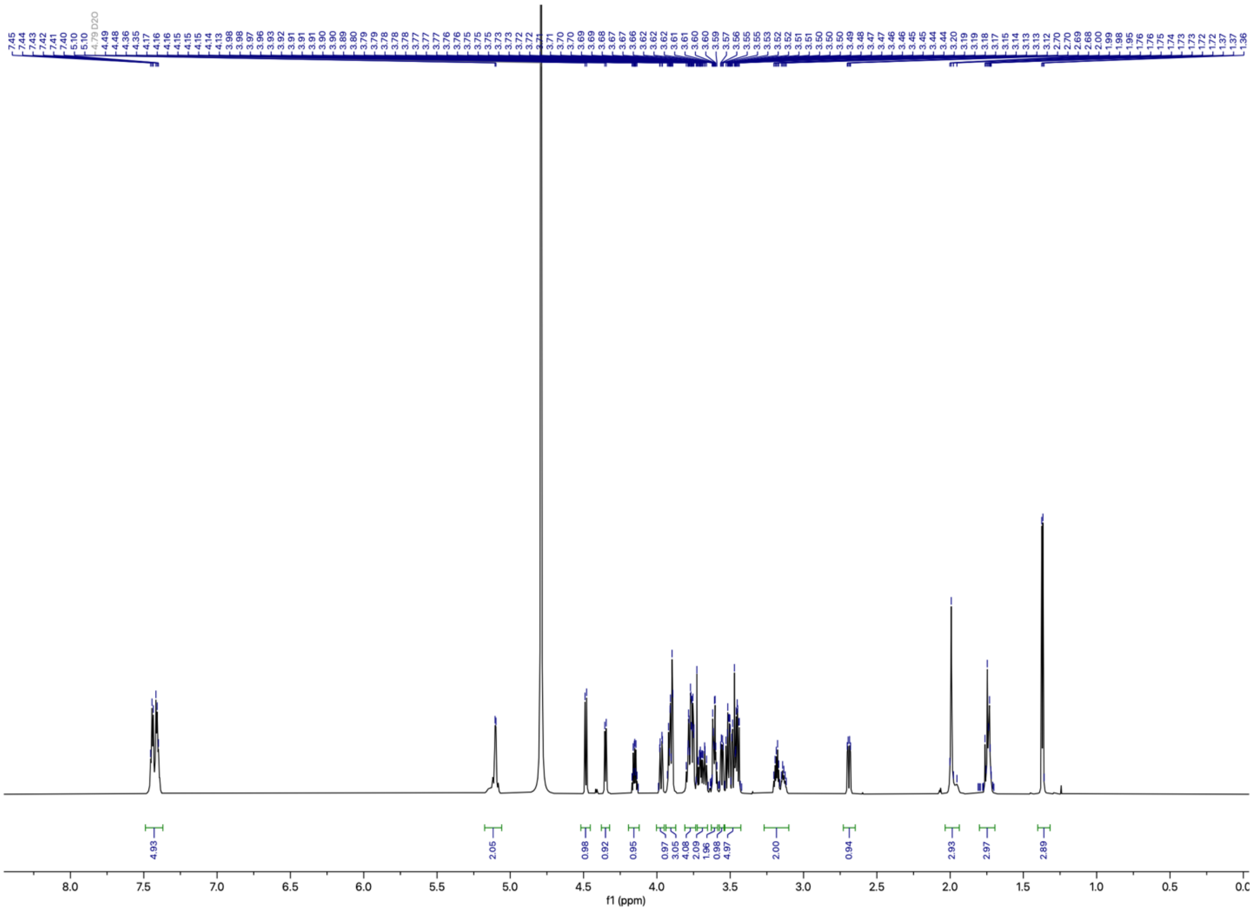

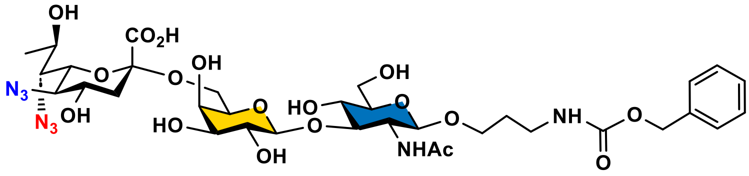

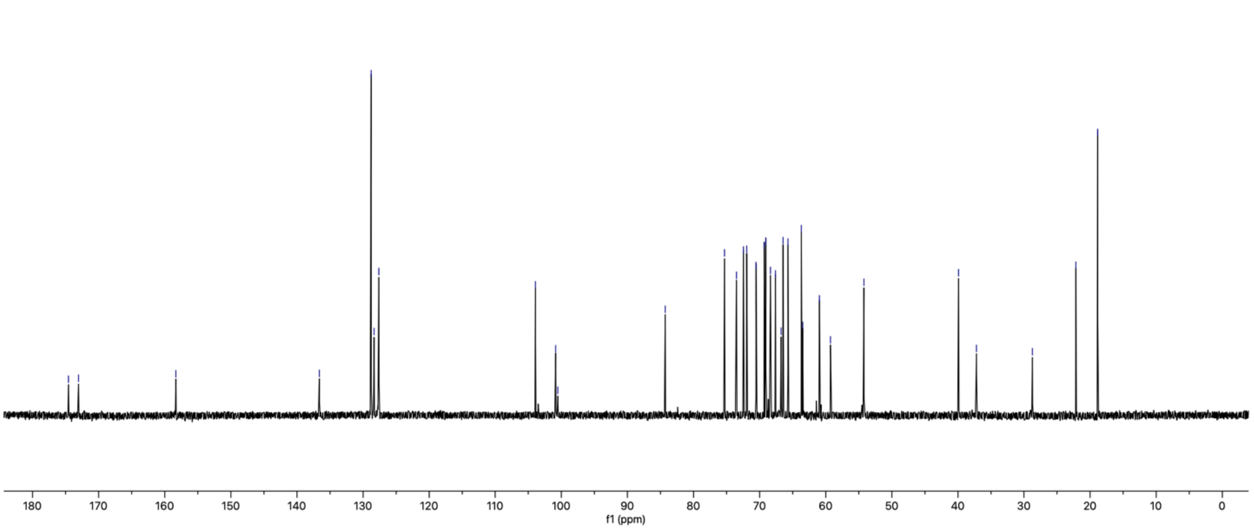

400 MHz ^{1}H and 100 MHz ^{13}C{^{1}H} NMR spectra of Leg5,7diN$_3$α2–6Galβ1–3GlcNAcαProNHCbz (**18**) in D_2O.

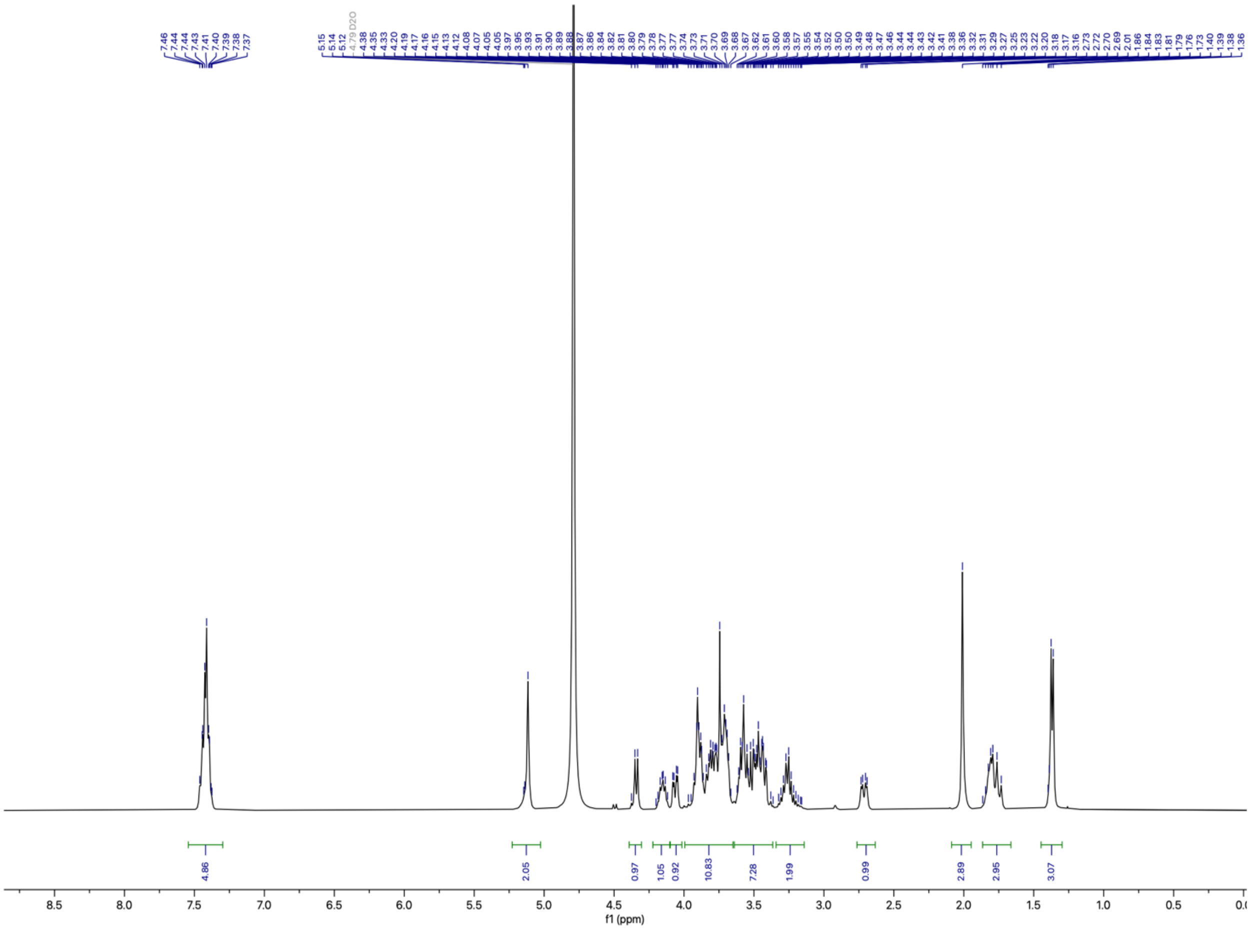

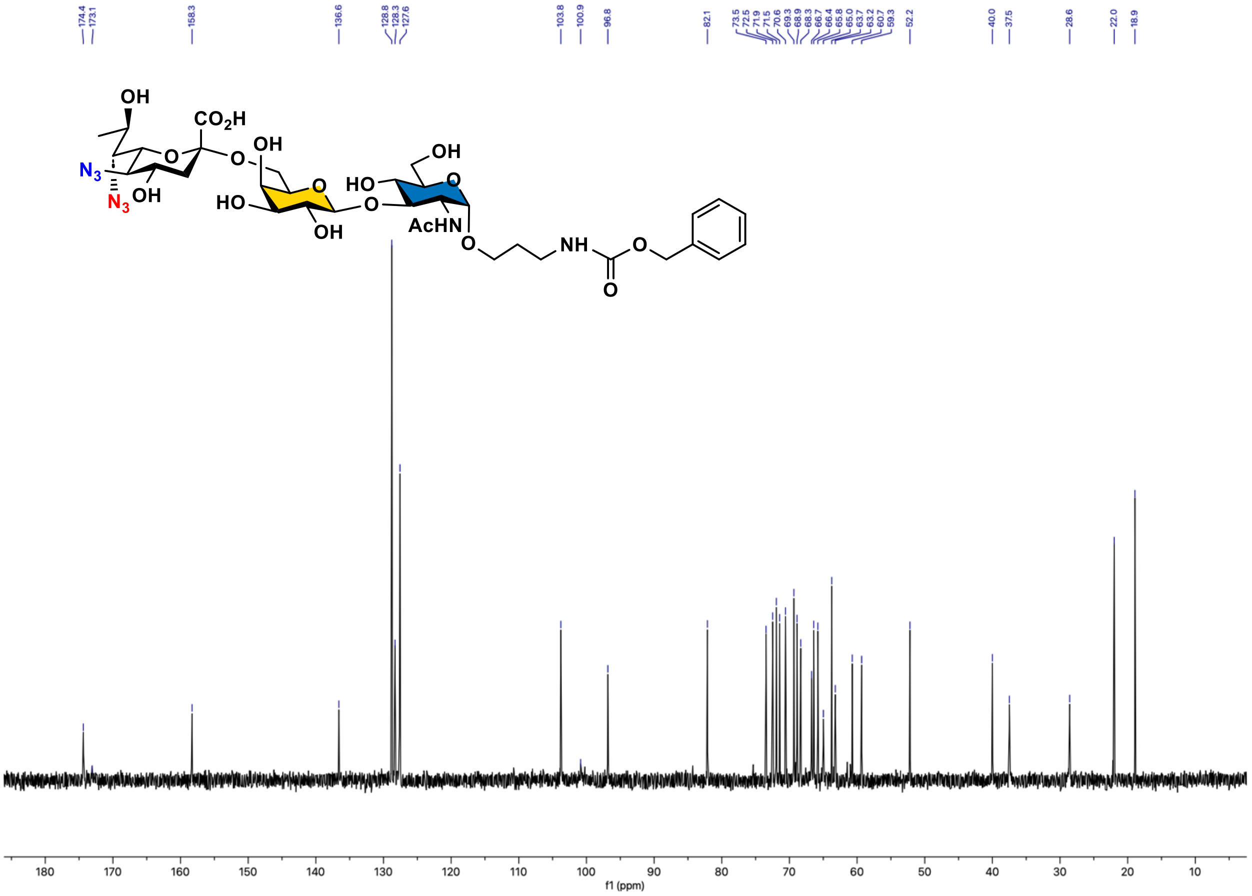

400 MHz ^{1}H and 100 MHz $^{13}C\{^{1}H\}$ NMR spectra of Leg5,7diN$_3$α2−3LacβProNHCbz (**19**) in D_2O.

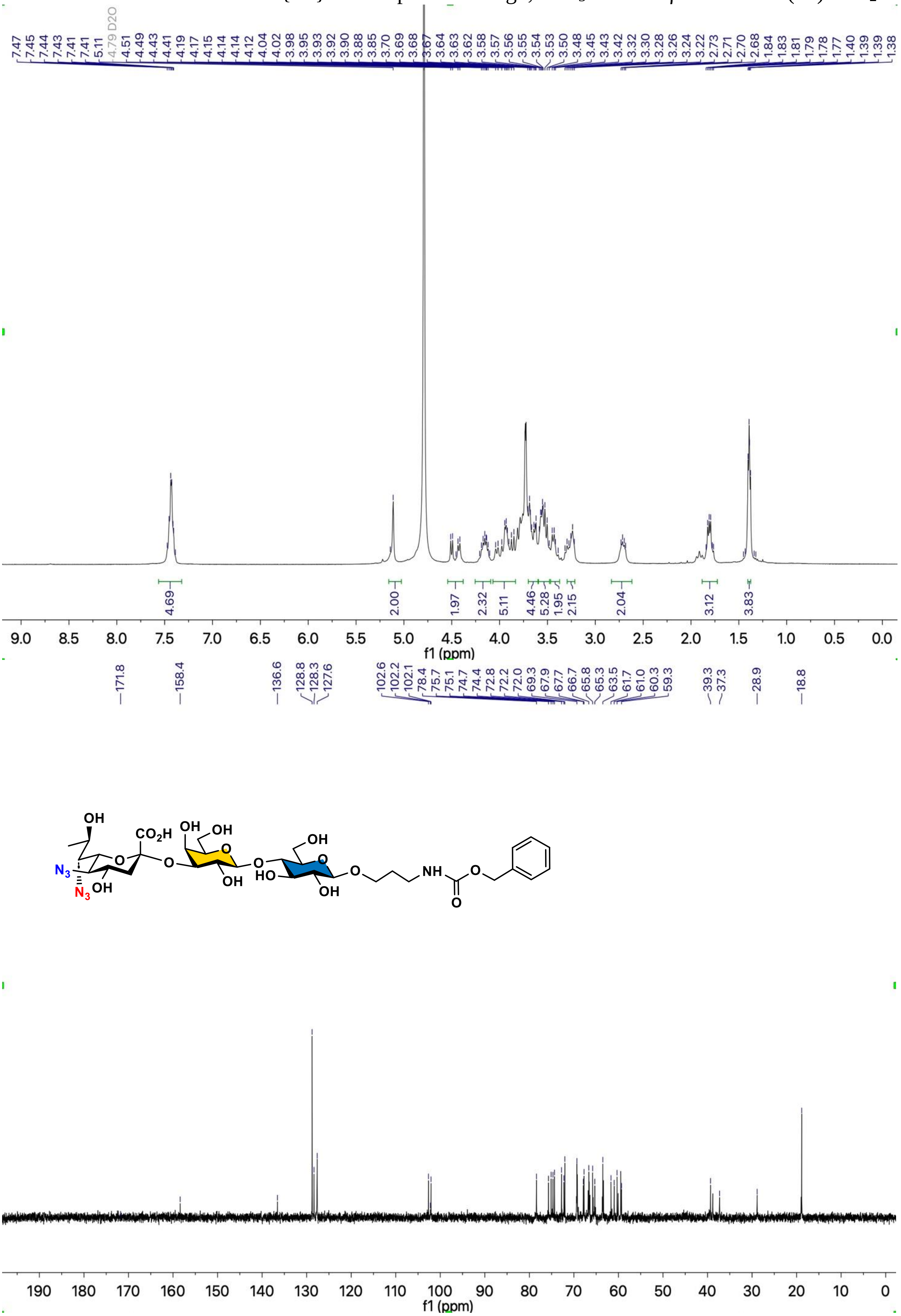

600 MHz ^{1}H and 150 MHz $^{13}C\{^{1}H\}$ NMR spectra of Leg5,7diN$_3$α2–3LacNAcβProNHCbz (**20**) in D_2O.

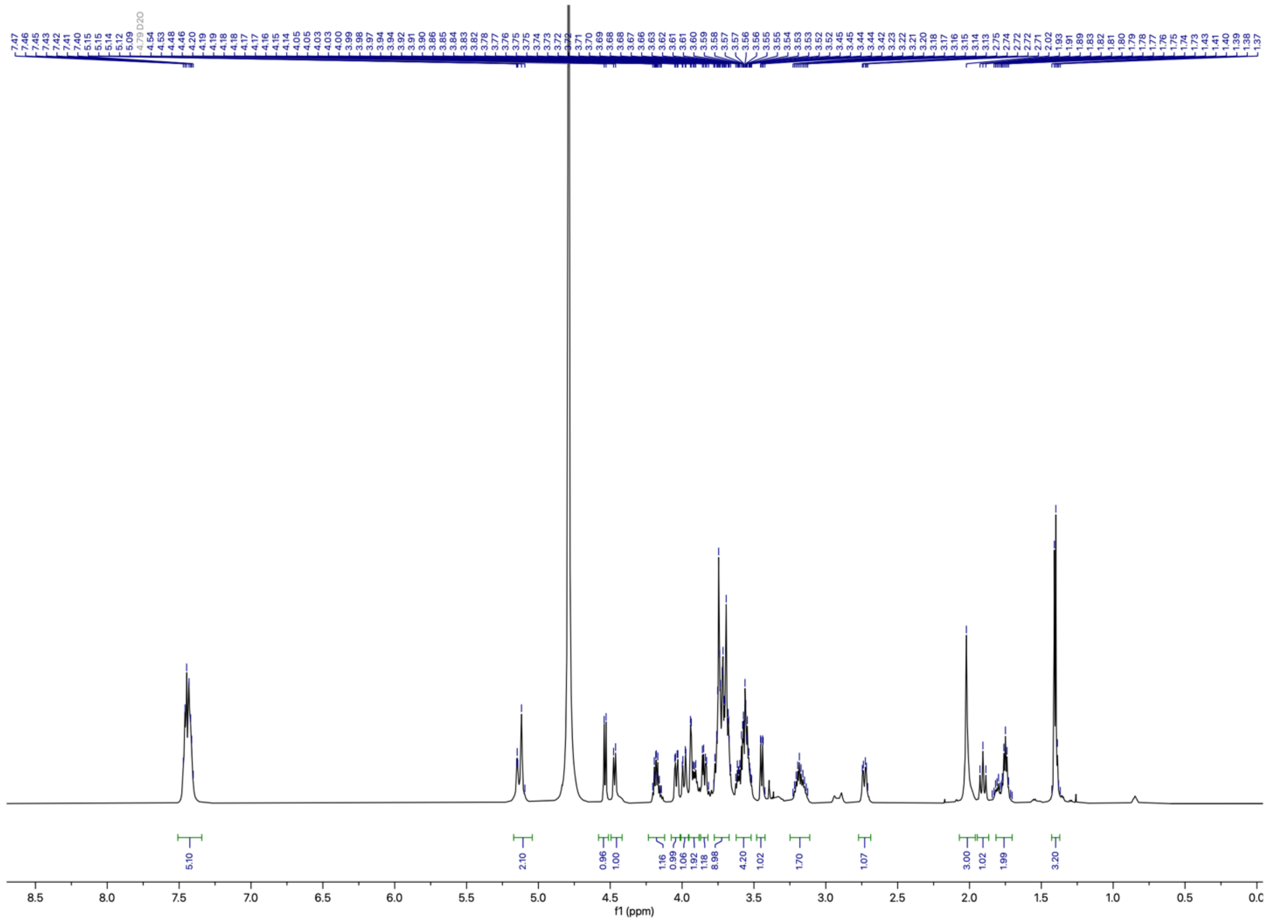

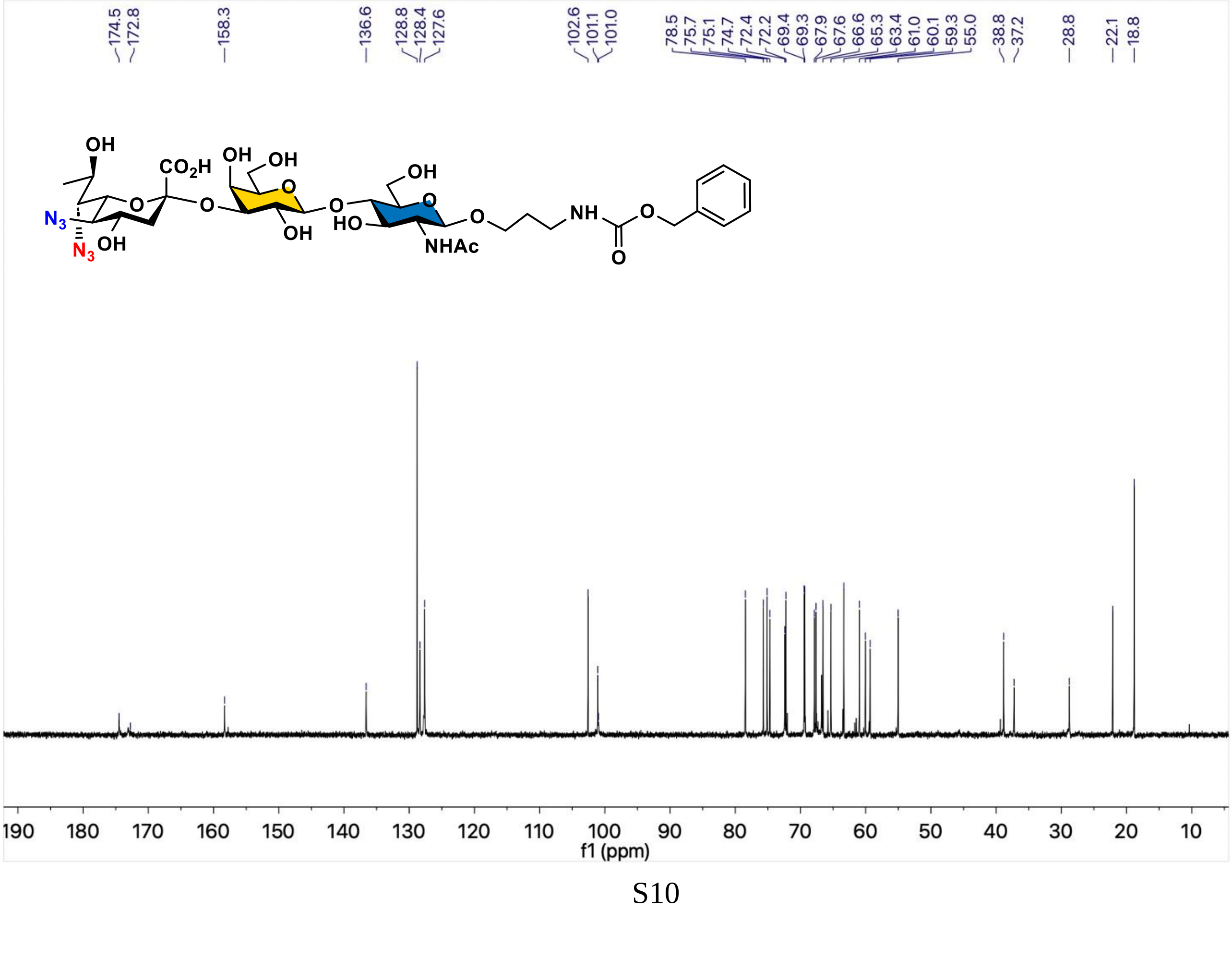

400 MHz ^{1}H and 100 MHz ^{13}C{^{1}H} NMR spectra of Leg5,7diN$_3$α2–3Galβ1–3GalNAcβProNHCbz (**21**) in D_2O.

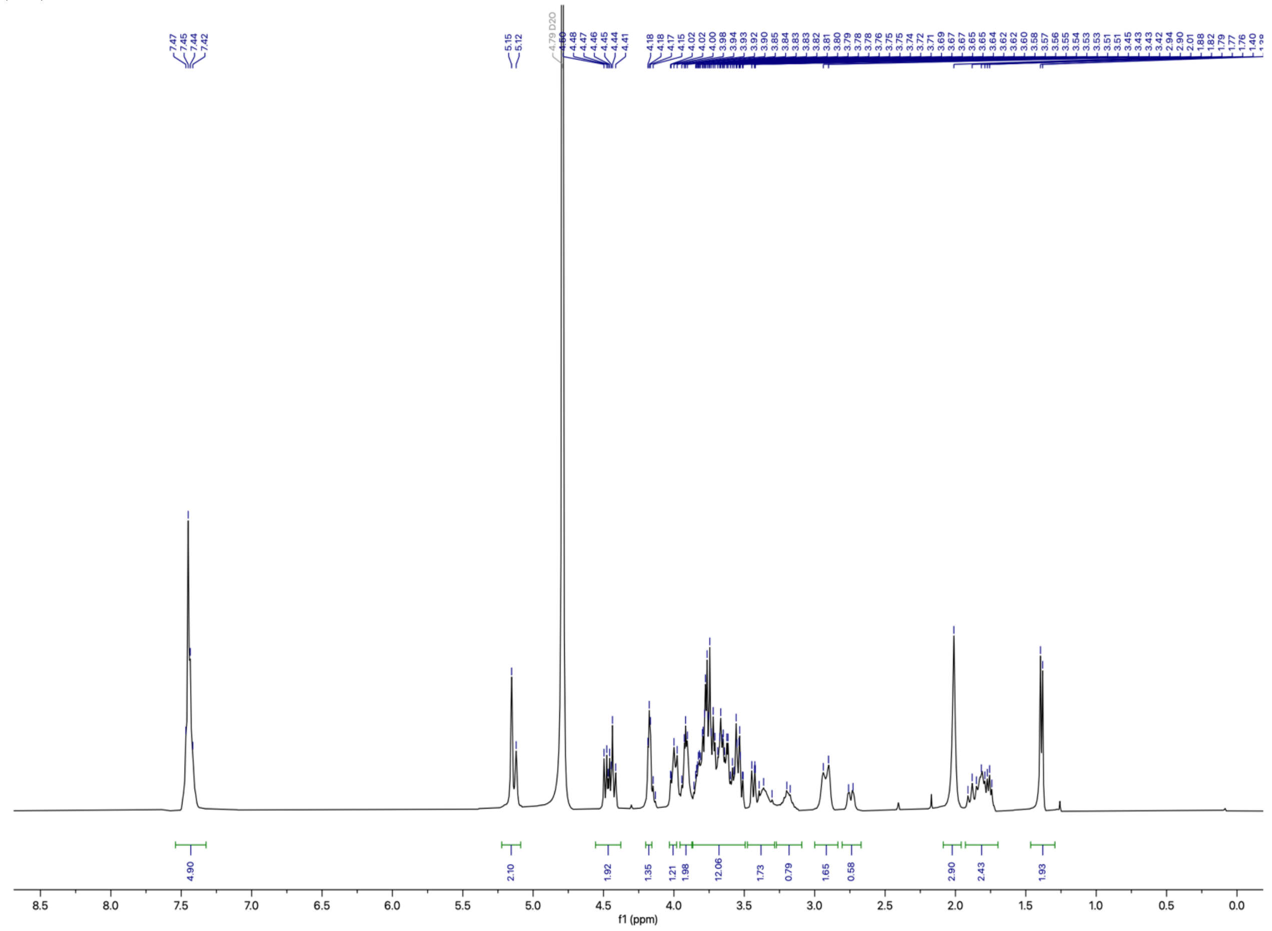

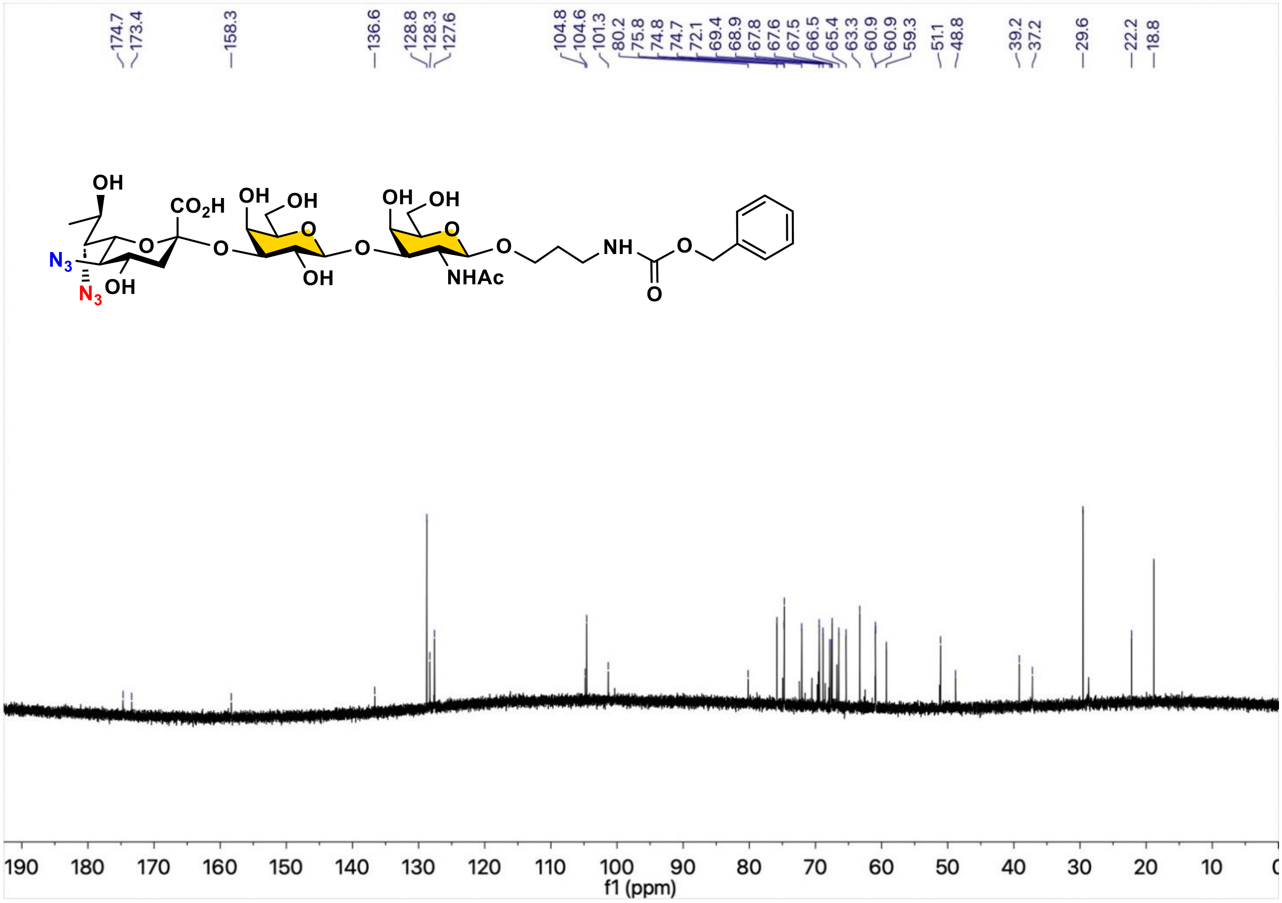

400 MHz ^{1}H and 100 MHz $^{13}C\{^{1}H\}$ NMR spectra of Leg5,7diN$_3$α2–3Galβ1–3GalNAcαProNHCbz (**22**) in D_2O.

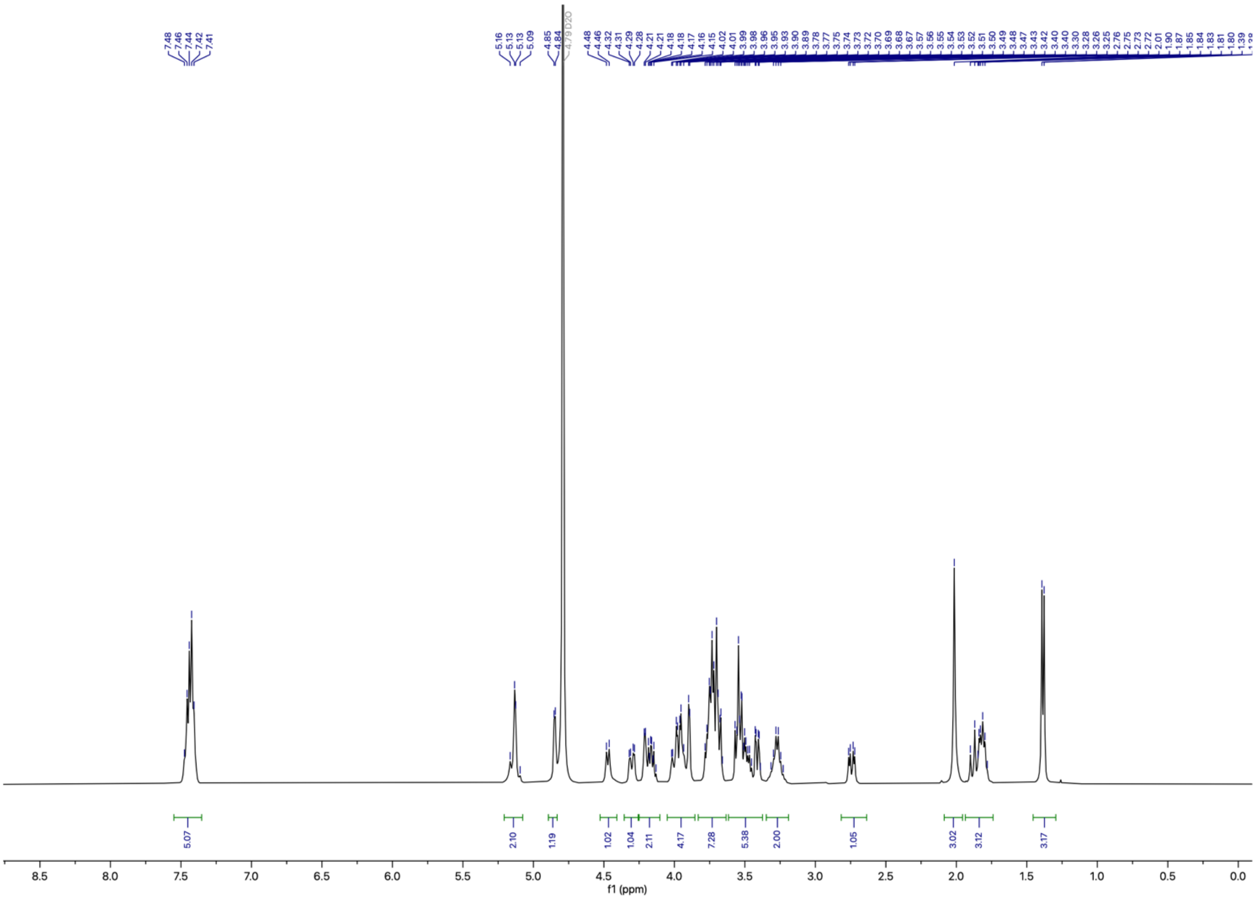

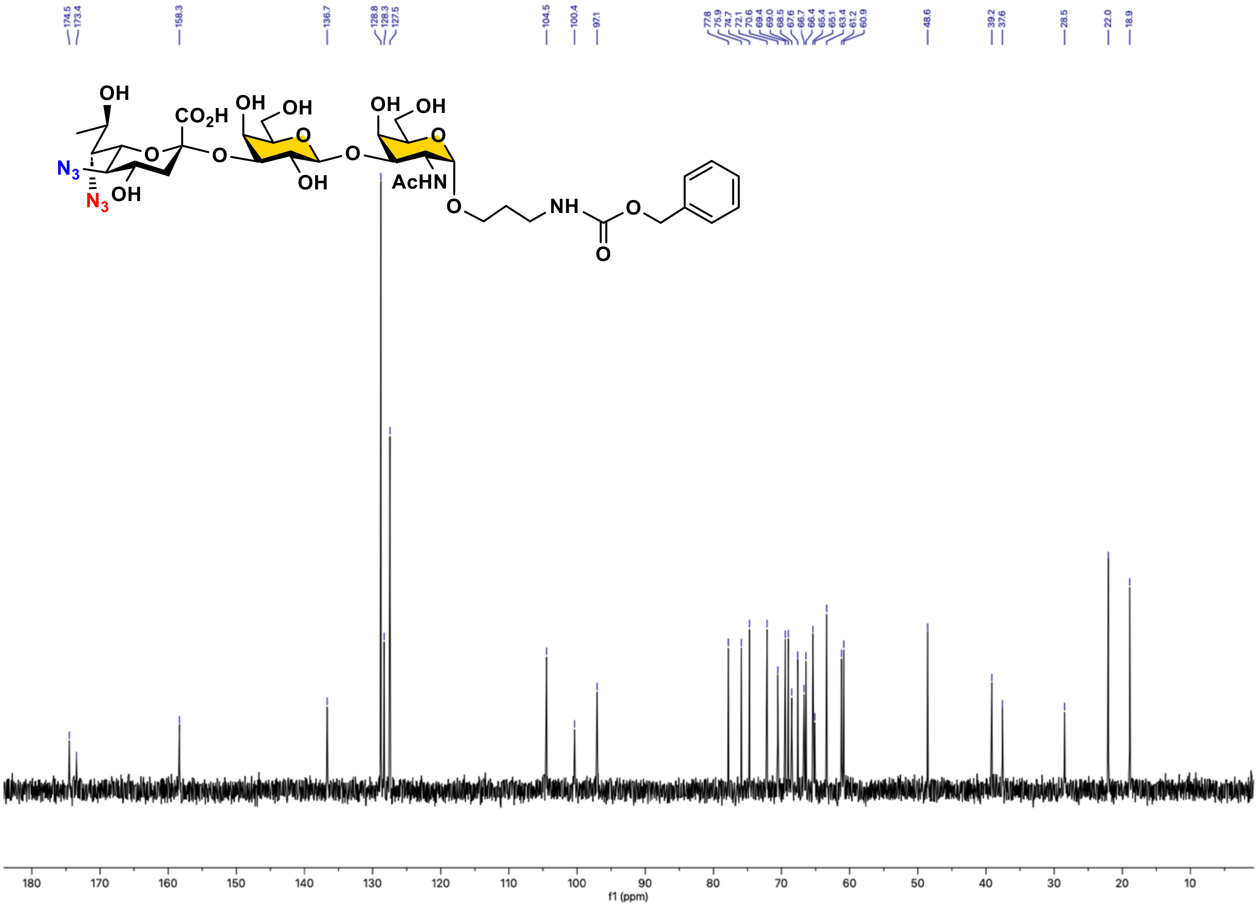

800 MHz ^{1}H and 200 MHz $^{13}C\{^{1}H\}$ NMR spectra of Leg5,7diN$_3$α2–3Galβ1–3GlcNAcβProNHCbz (**23**) in D_2O.

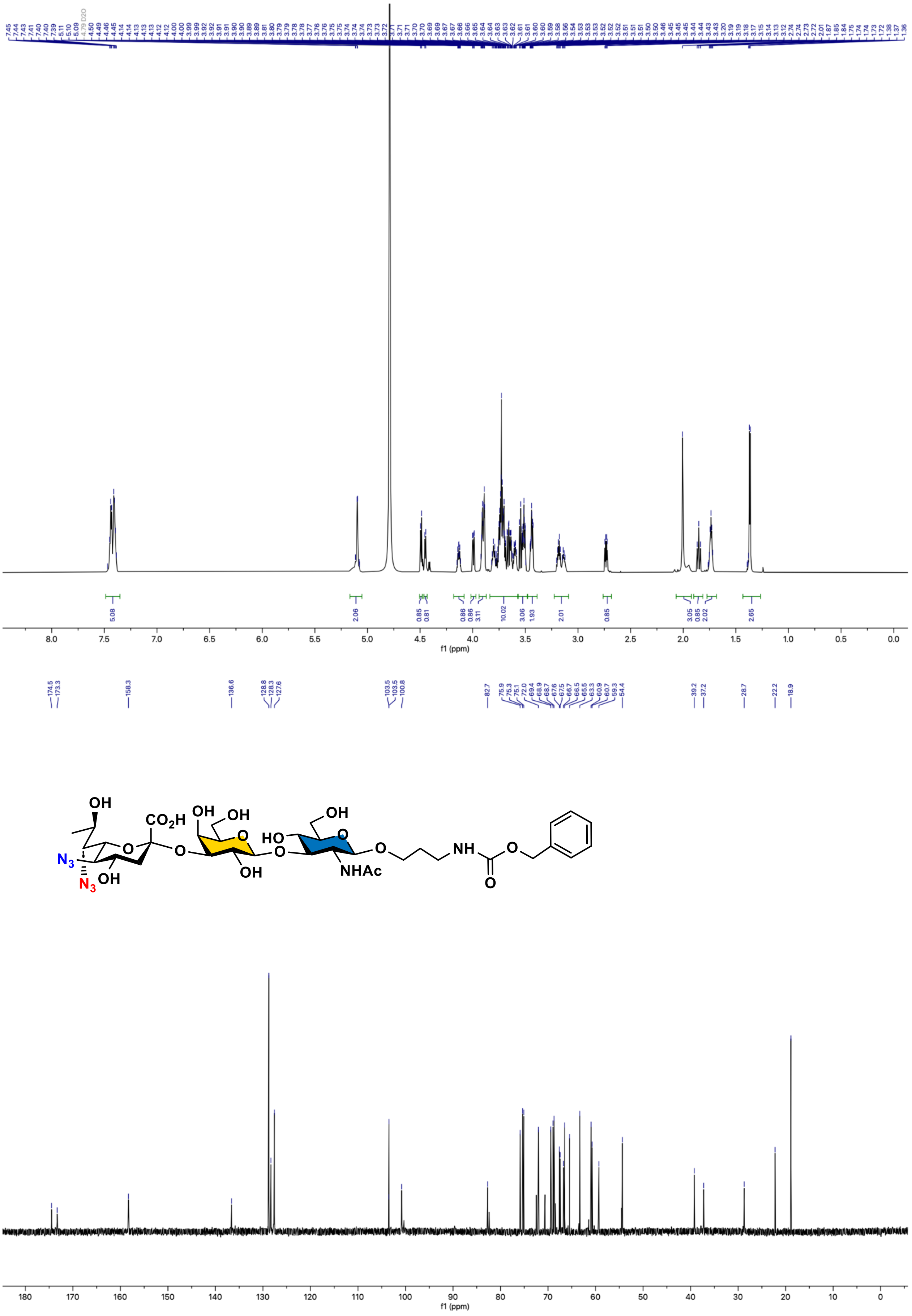

400 MHz ^{1}H and 100 MHz $^{13}C\{^{1}H\}$ NMR spectra of Leg5,7diN$_3$α2–3Galβ1–3GlcNAcαProNHCbz (**24**) in D_2O.

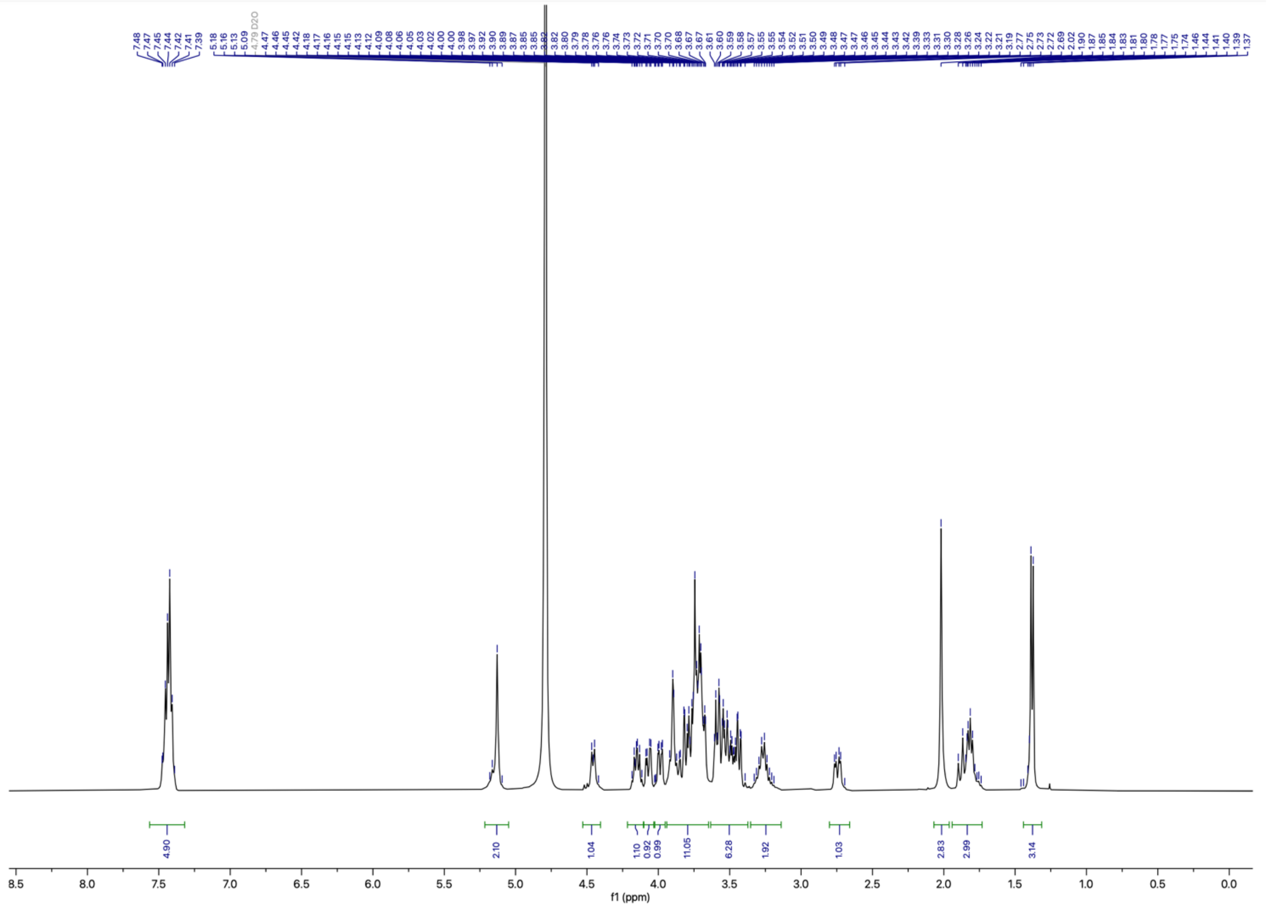

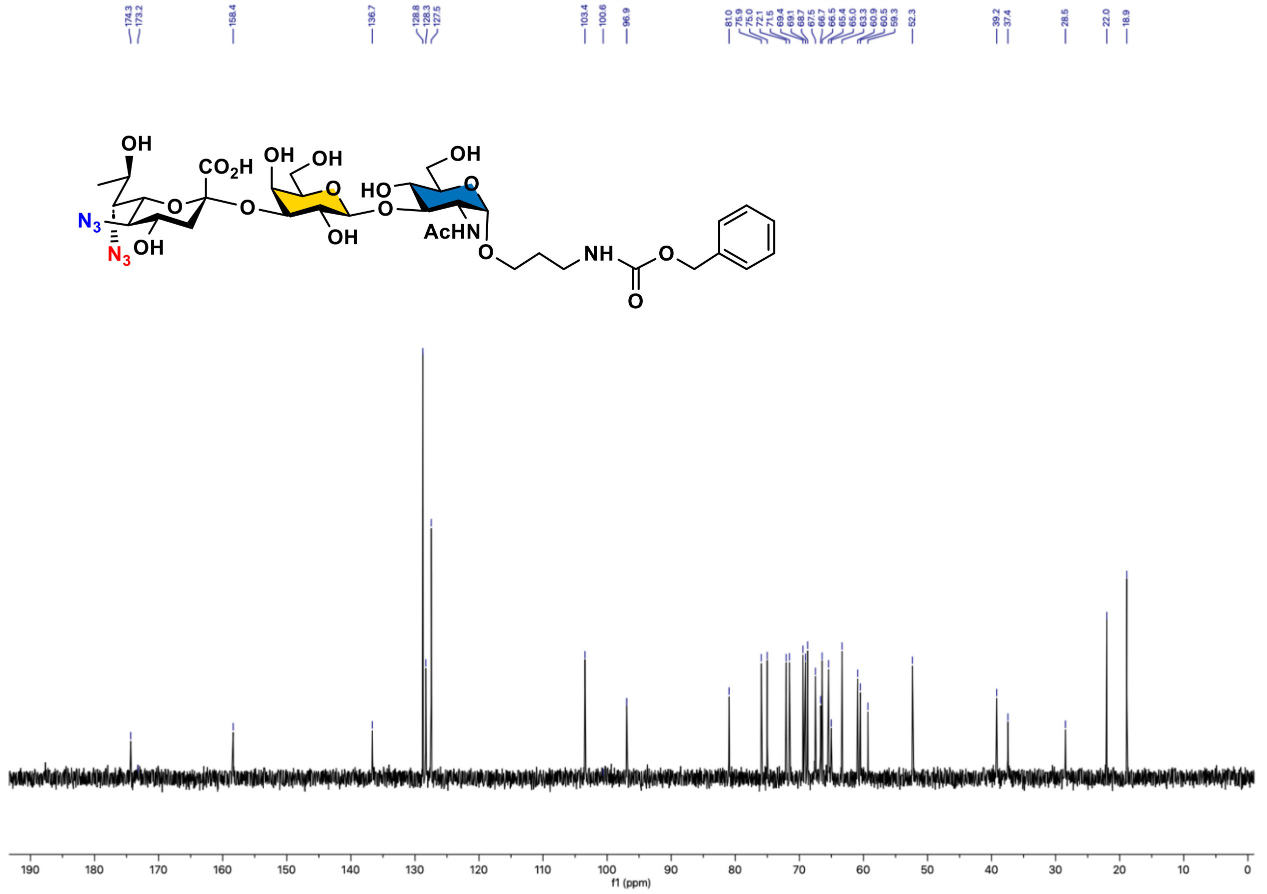

400 MHz ^{1}H and 100 MHz $^{13}C\{^{1}H\}$ NMR spectra of Leg5,7Ac$_2$α2–6GalNAcαProNHCbz (**25**) in D_2O.

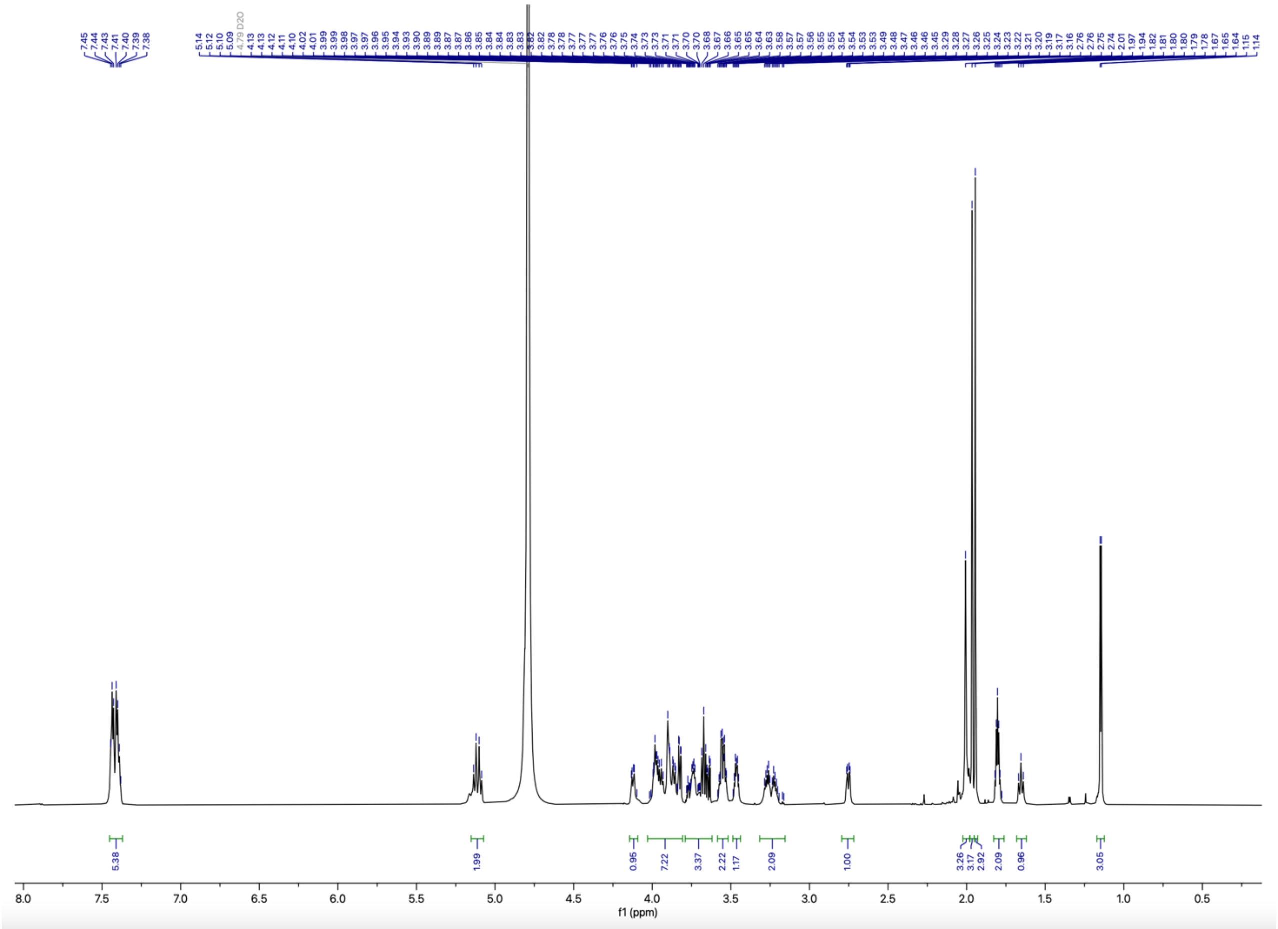

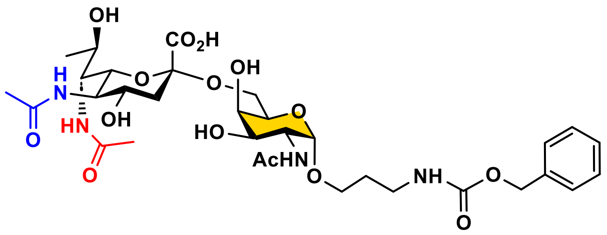

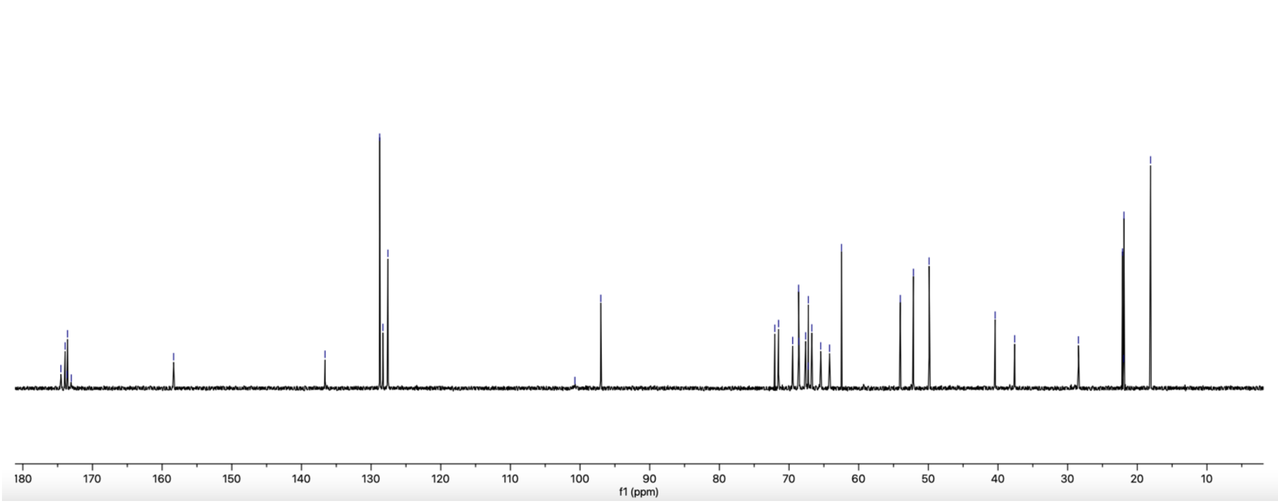

400 MHz ^{1}H and 100 MHz $^{13}C\{^{1}H\}$ NMR spectra of Leg5,7Ac$_2$α2–6LacβProNHCbz (**26**) in D_2O.

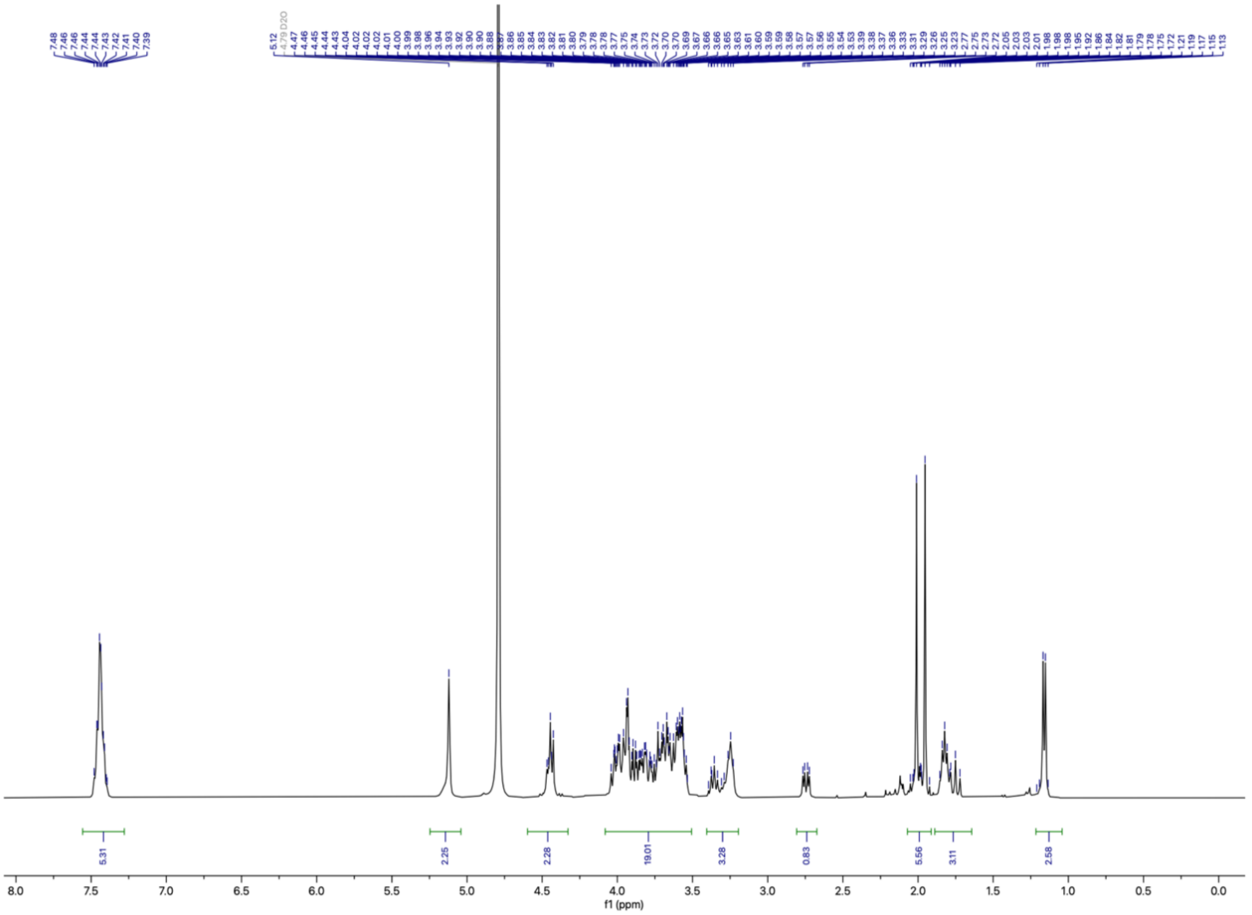

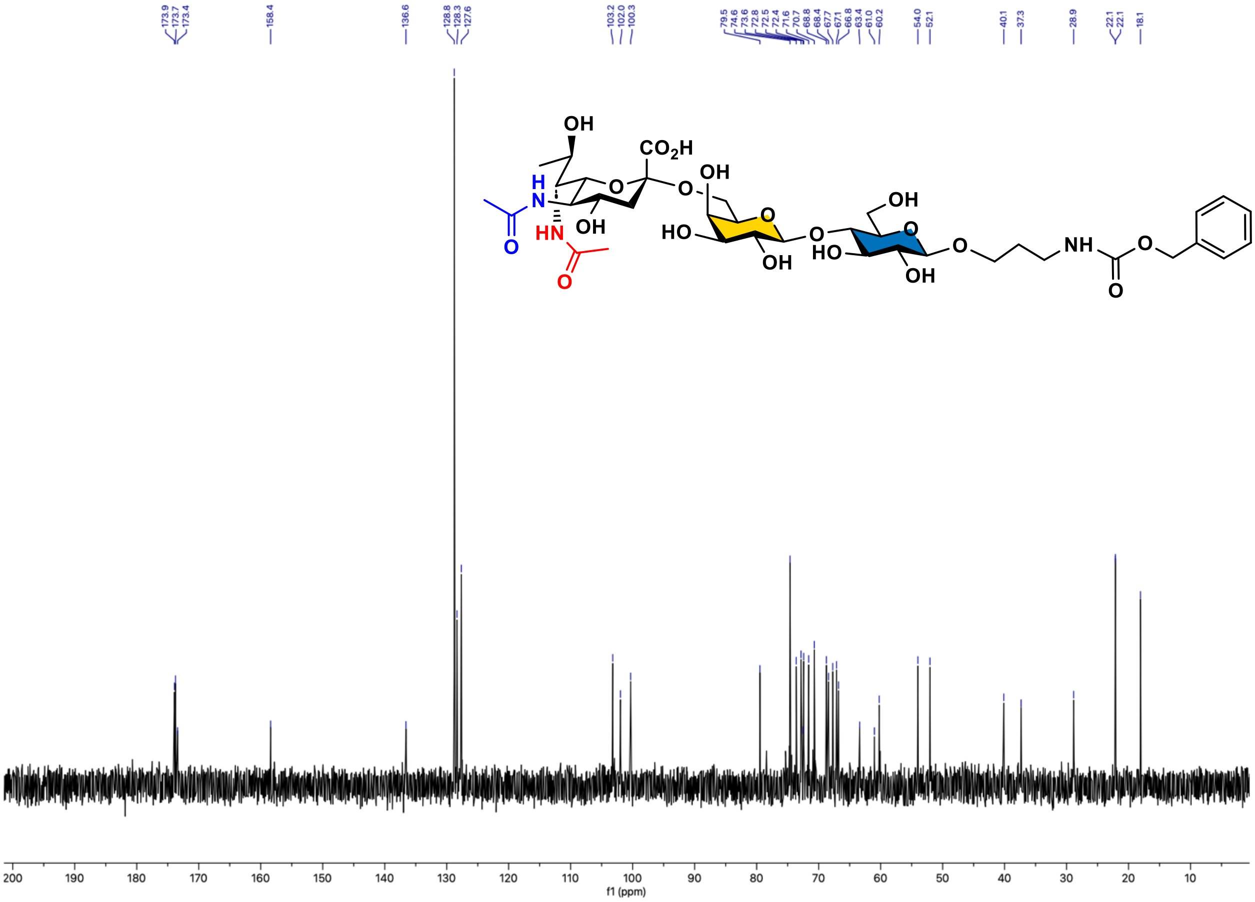

800 MHz ^{1}H and 200 MHz $^{13}C\{^{1}H\}$ NMR spectra of Leg5,7Ac$_2$α2–6LacNAcβProNHCbz (**27**) in D_2O.

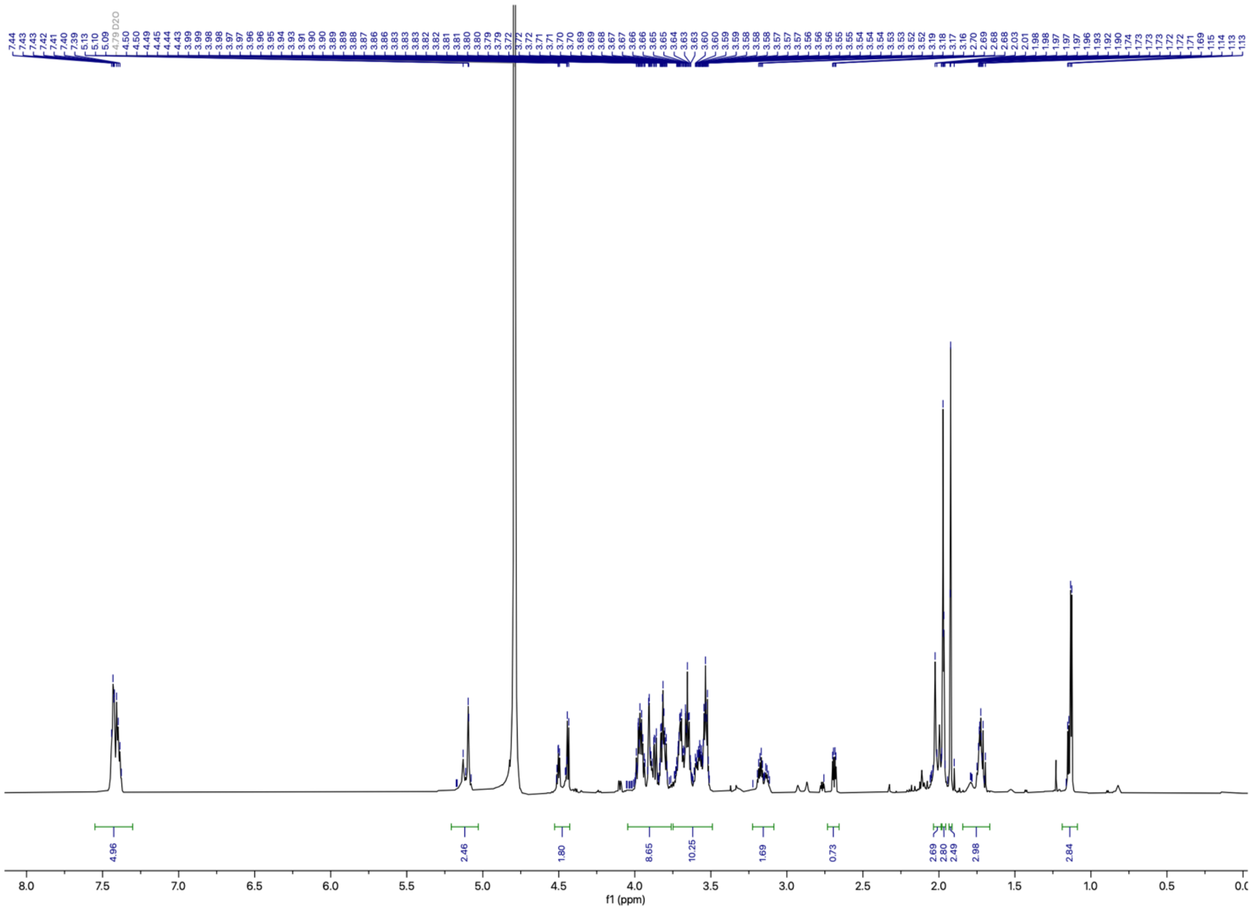

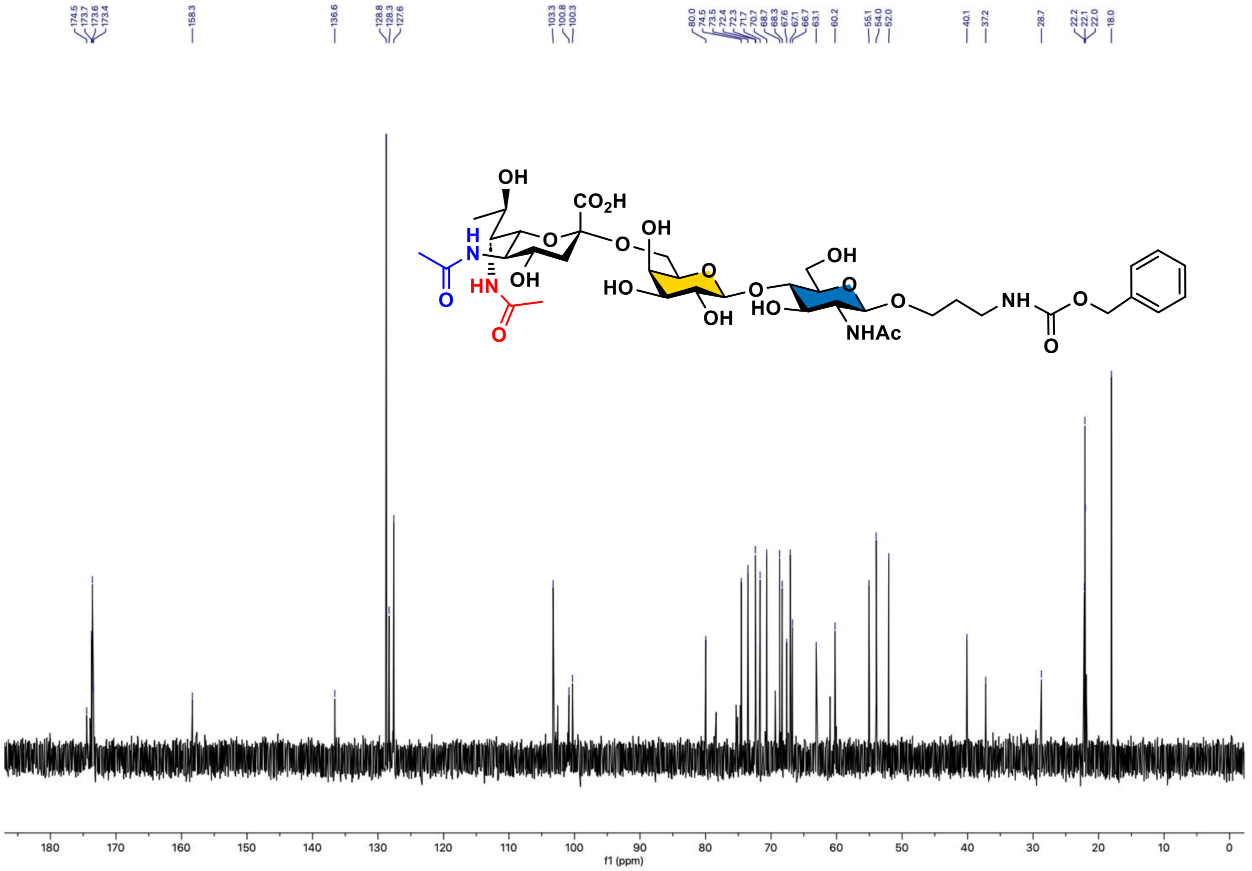

800 MHz ^{1}H and 200 MHz ^{13}C{^{1}H} NMR spectra of Leg5,7Ac$_2$α2–6Galβ1–3GalNAcβProNHCbz (**28**) in D_2O.

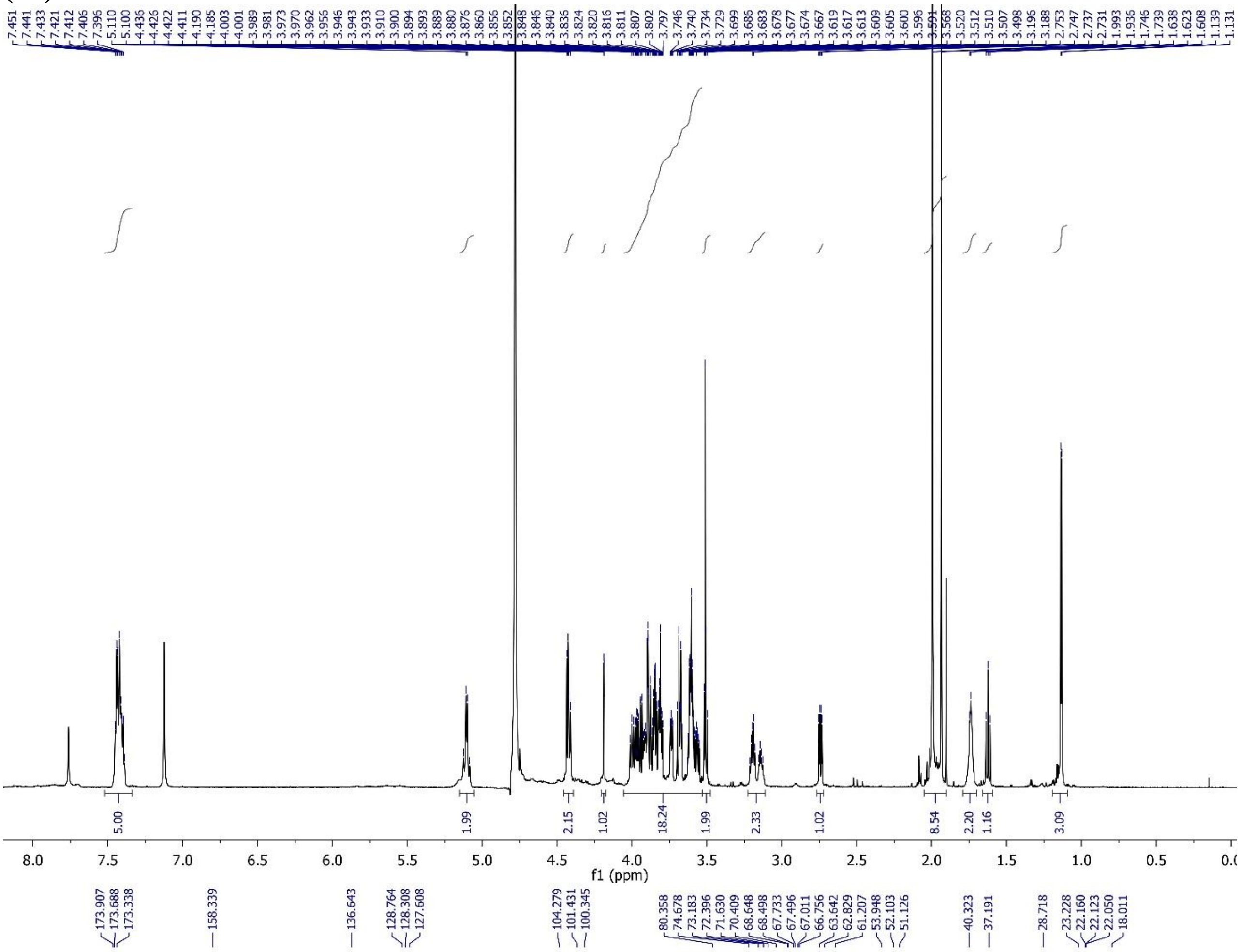

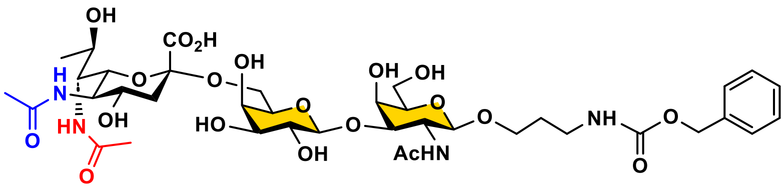

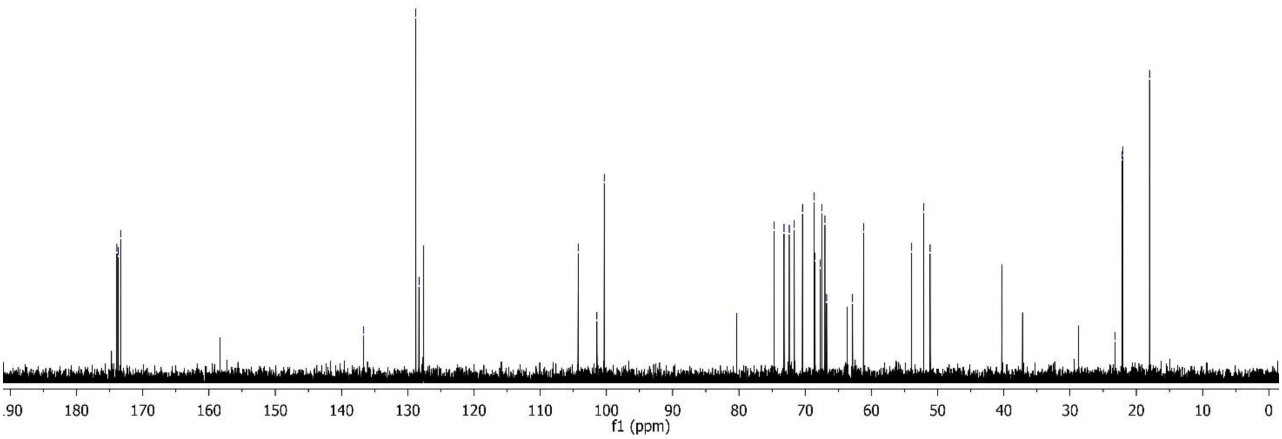

800 MHz ^{1}H and 200 MHz ^{13}C{^{1}H} NMR spectra of Leg5,7Ac$_2$α2–6Galβ1–3GalNAcαProNHCbz (**29**) in D_2O.

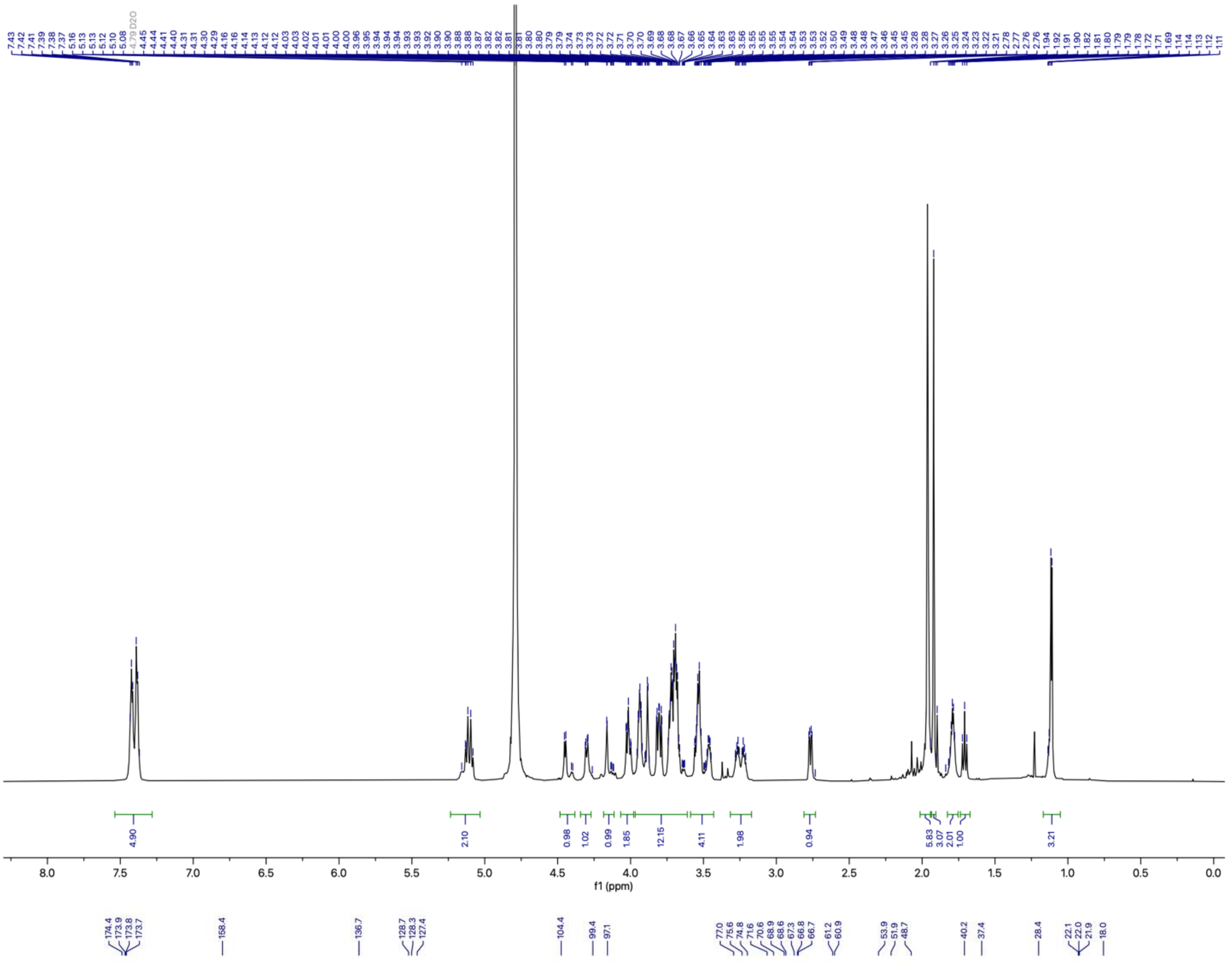

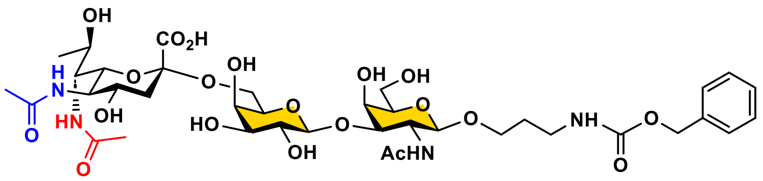

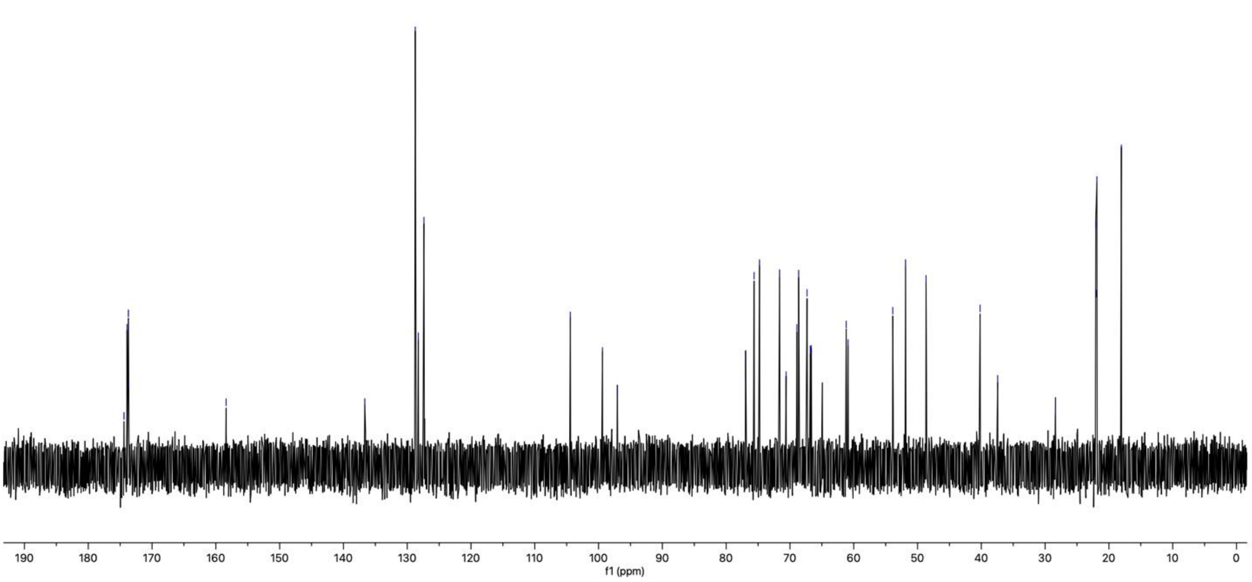

800 MHz ^{1}H and 200 MHz $^{13}C\{^{1}H\}$ NMR spectra of Leg5,7Ac$_2$α2–6Galβ1–3GlcNAcβProNHCbz (**30**) in D_2O.

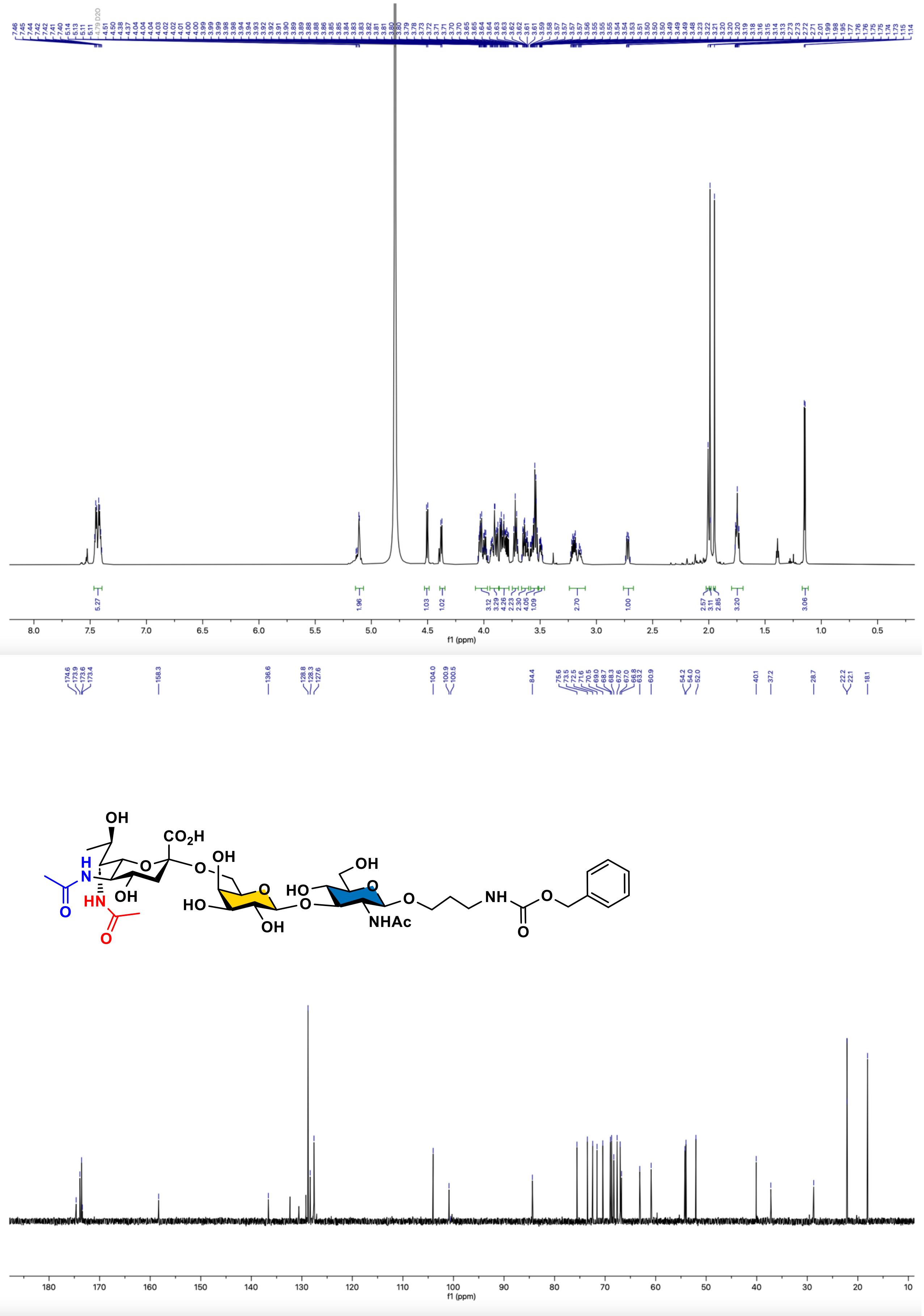

800 MHz ^{1}H and 200 MHz $^{13}C\{^{1}H\}$ NMR spectra of Leg5,7Ac$_2$α2–6Galβ1–3GlcNAcαProNHCbz (**31**) in D_2O.

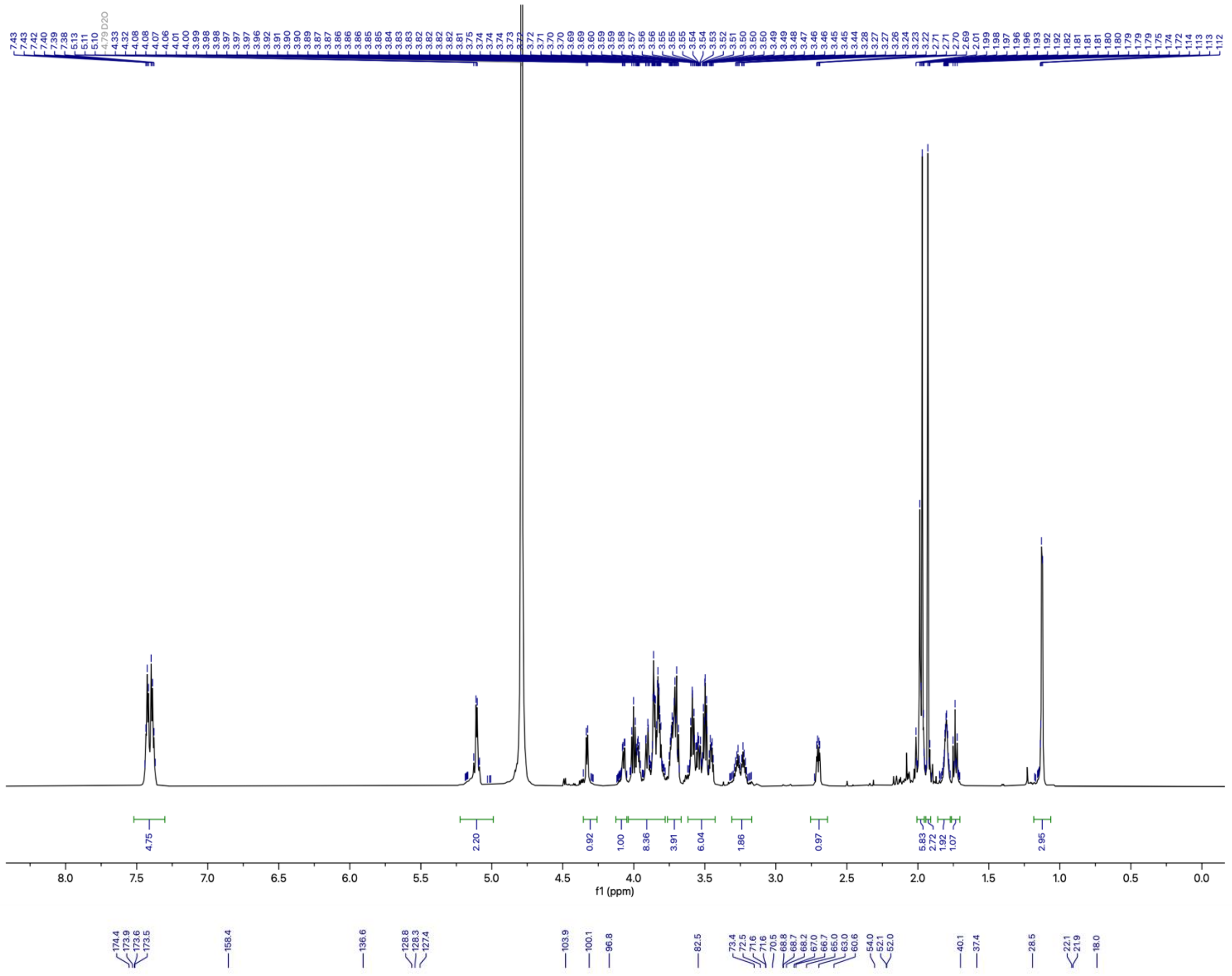

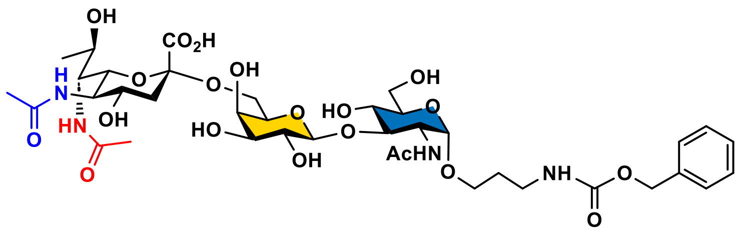

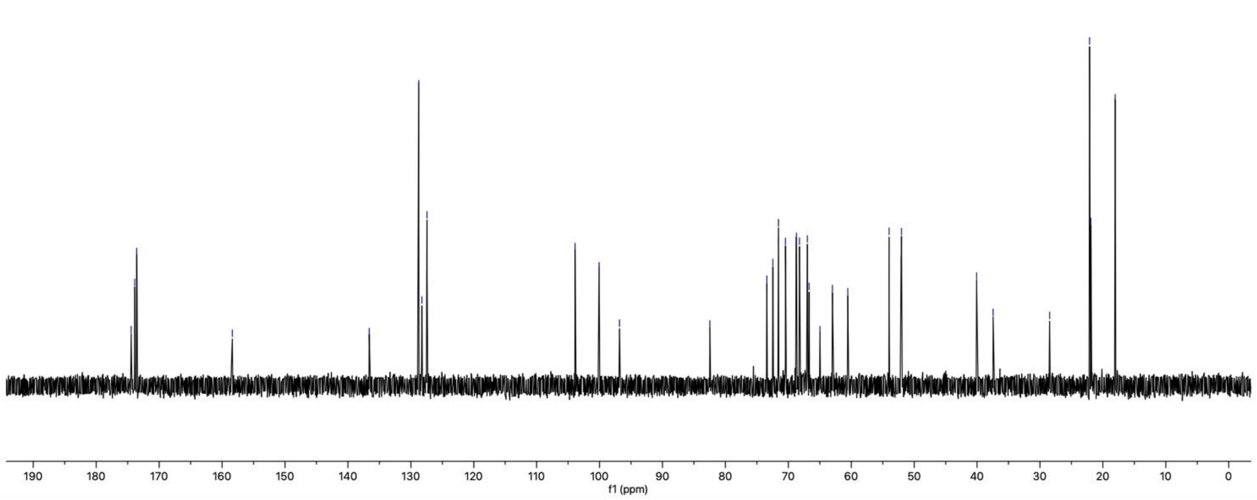

800 MHz ^{1}H and 200 MHz $^{13}C\{^{1}H\}$ NMR spectra of Leg5,7Ac$_2$α2–3LacβProNHCbz (**32**) in D_2O.

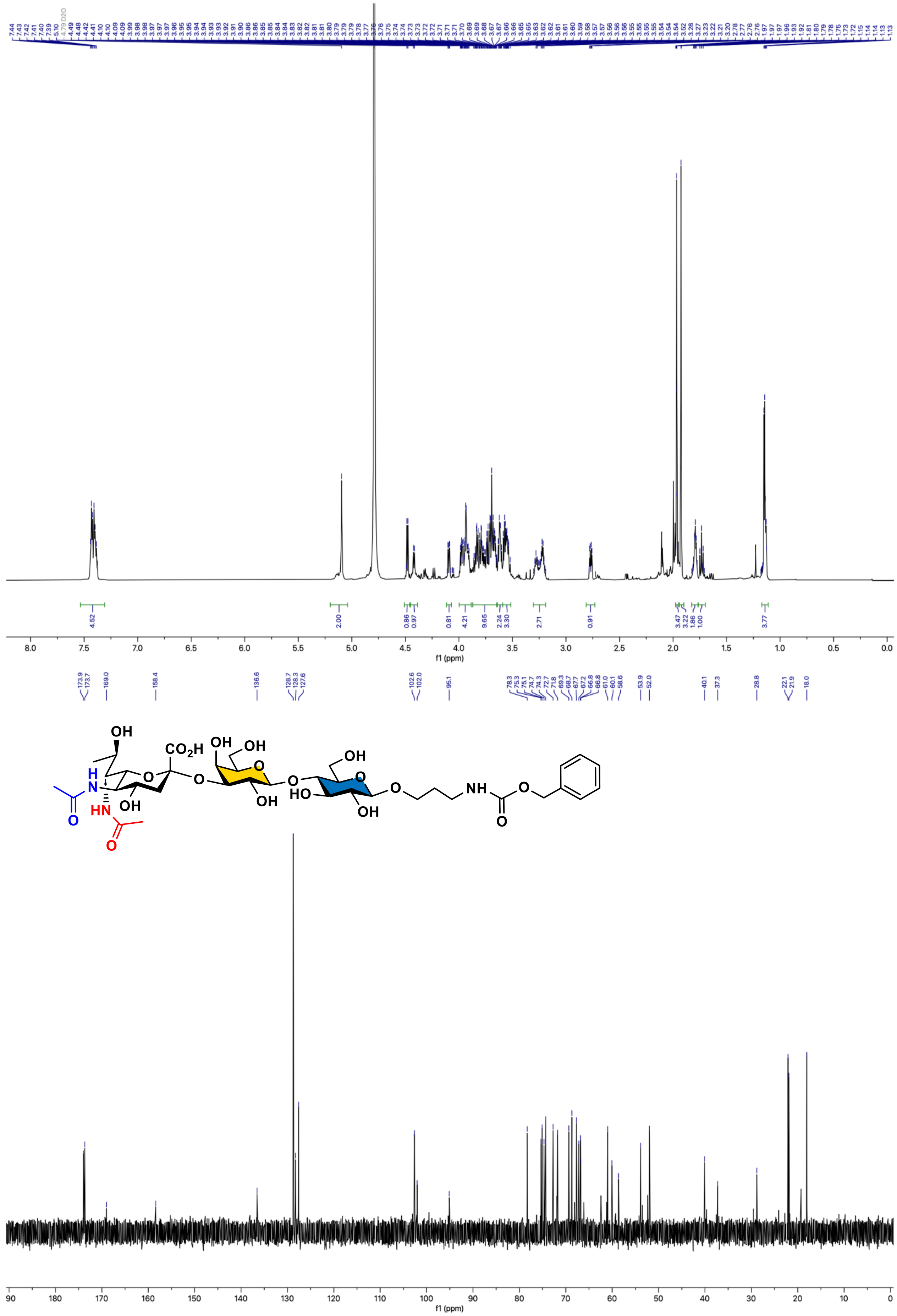

800 MHz ^{1}H and 200 MHz $^{13}C\{^{1}H\}$ NMR spectra of Leg5,7Ac$_2$α2–3LacNAcβProNHCbz (**33**) in D_2O.

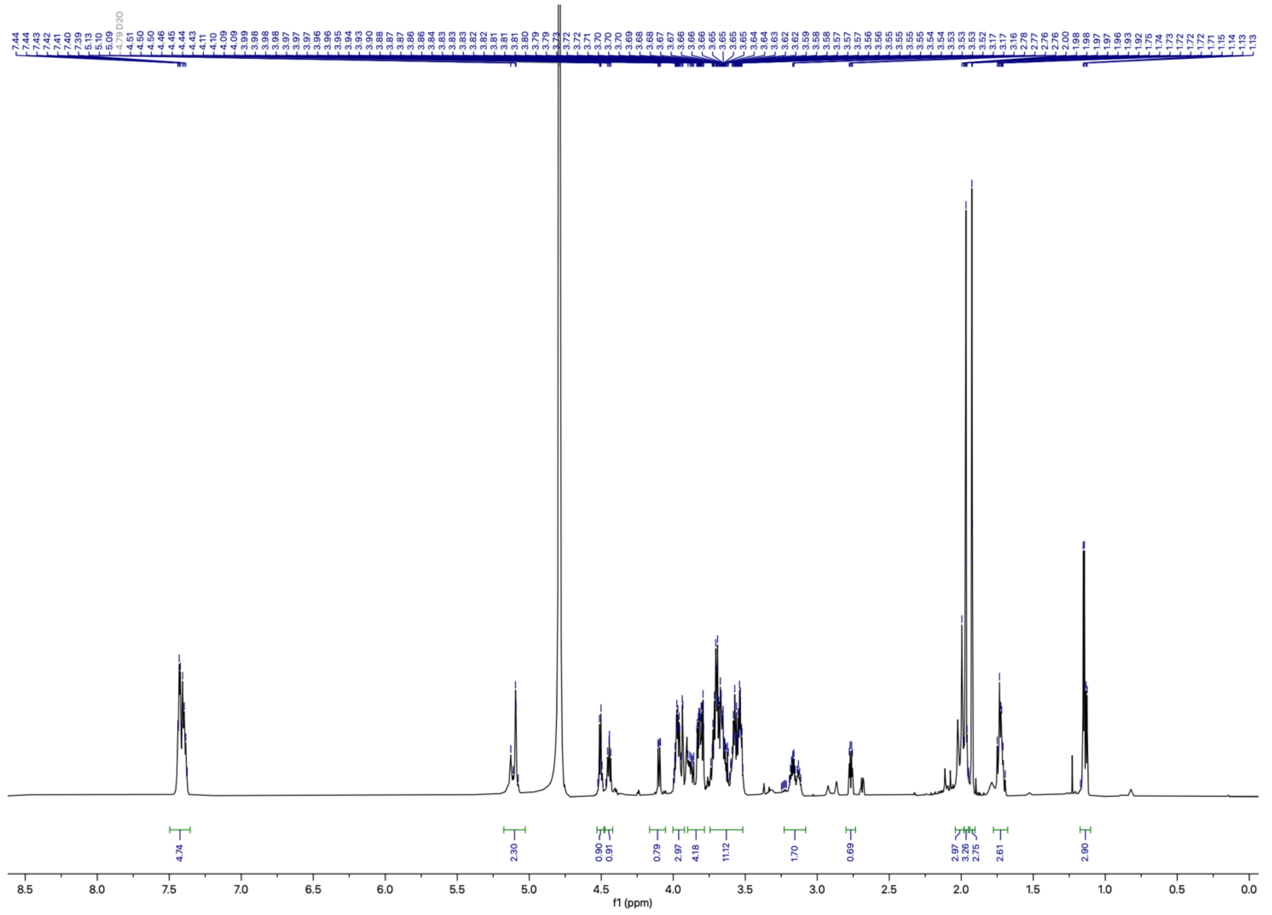

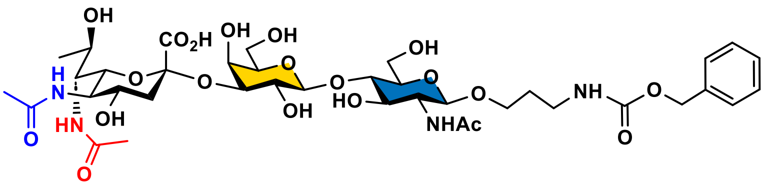

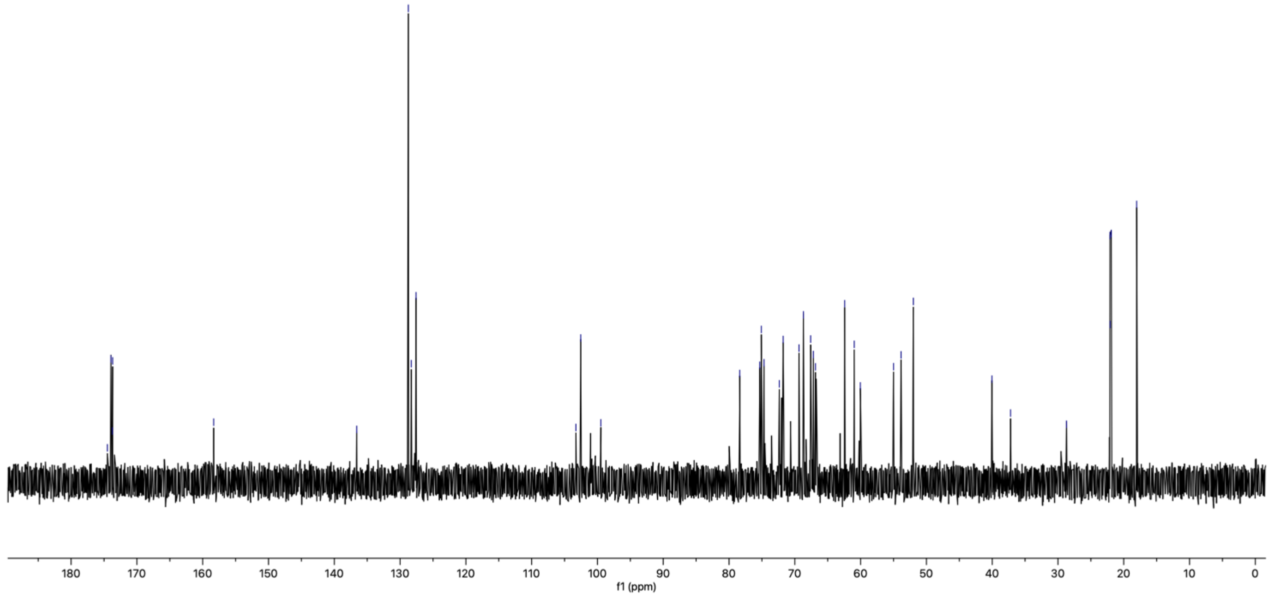

800 MHz ^{1}H and 200 MHz $^{13}C\{^{1}H\}$ NMR spectra of Leg5,7Ac$_2$α2–3Galβ1–3GalNAcβProNHCbz (**34**) in D_2O.

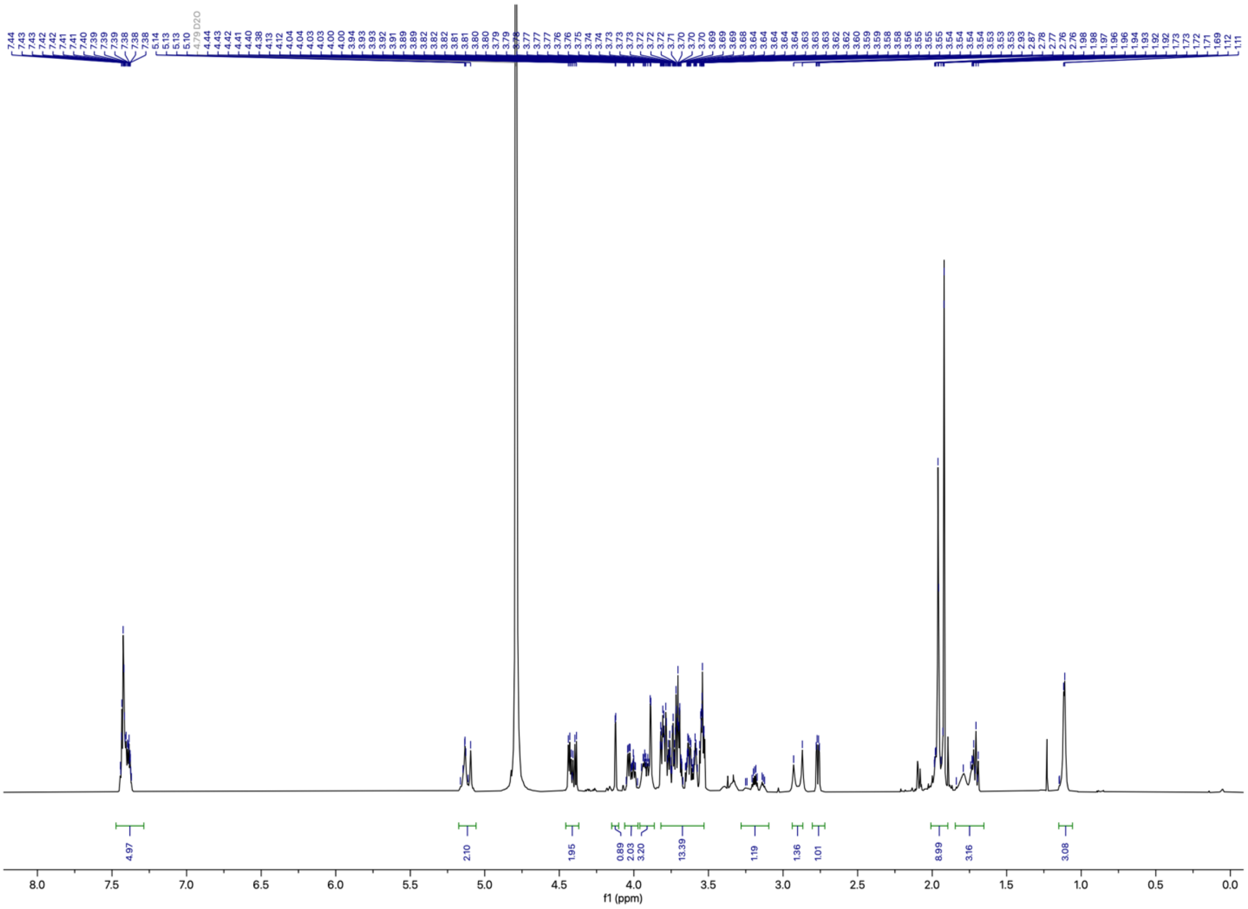

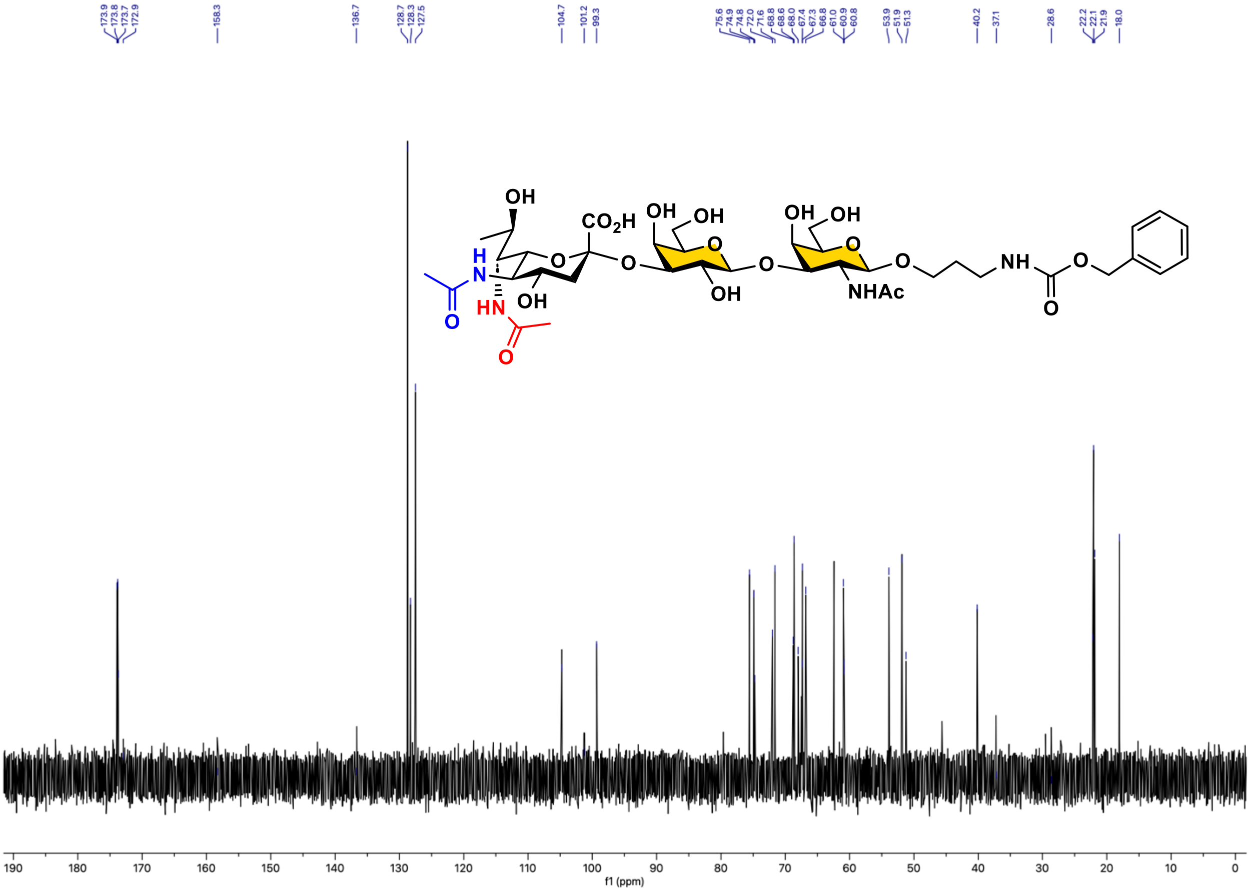

800 MHz ^{1}H and 200 MHz $^{13}C\{^{1}H\}$ NMR spectra of Leg5,7Ac$_2$α2–3Galβ1–3GalNAcαProNHCbz (**35**) in D_2O.

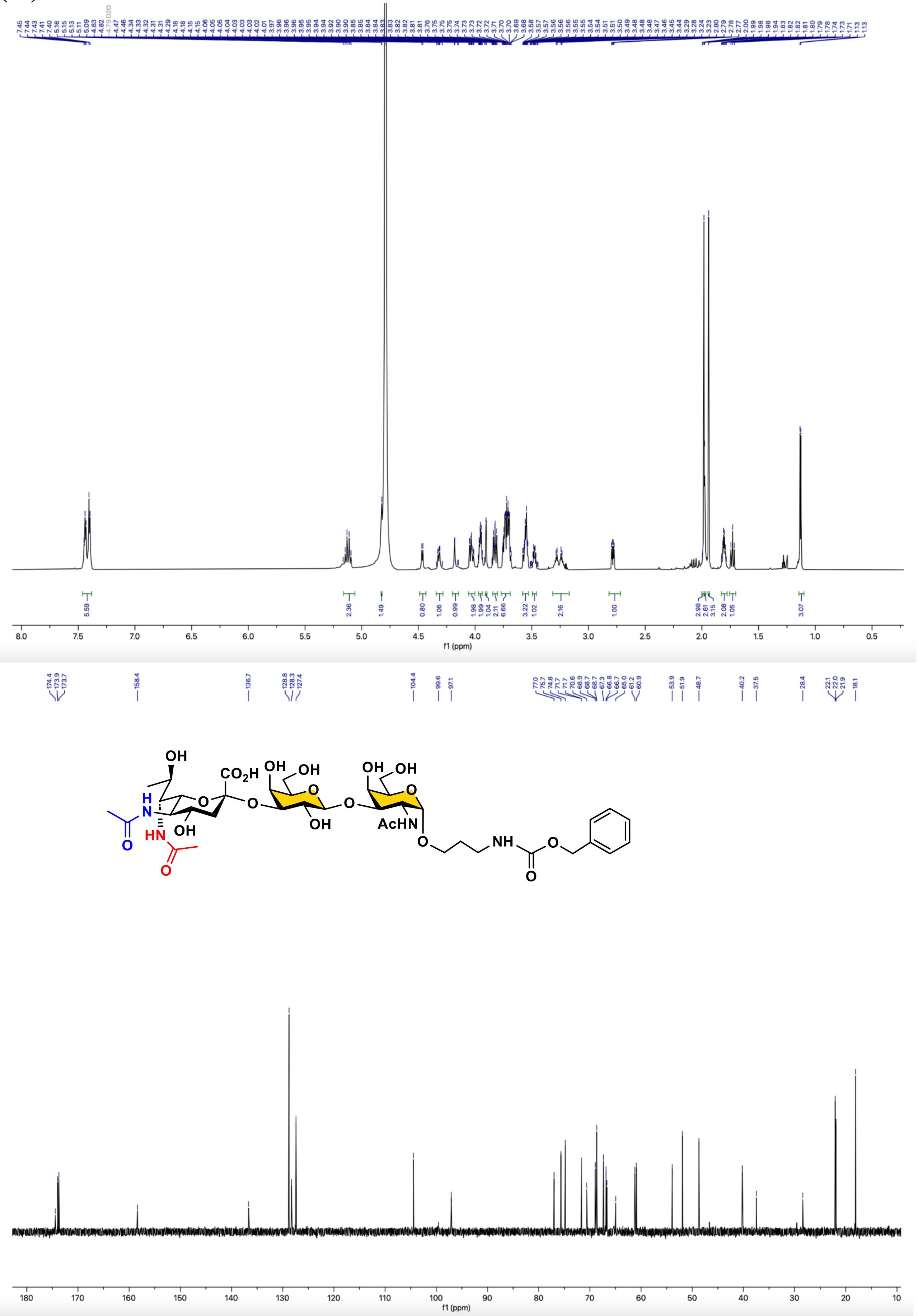

600 MHz ^{1}H and 150 MHz ^{13}C{^{1}H} NMR spectra of Leg5,7Ac$_2$α2–3Galβ1–3GlcNAcβProNHCbz (**36**) in D_2O.

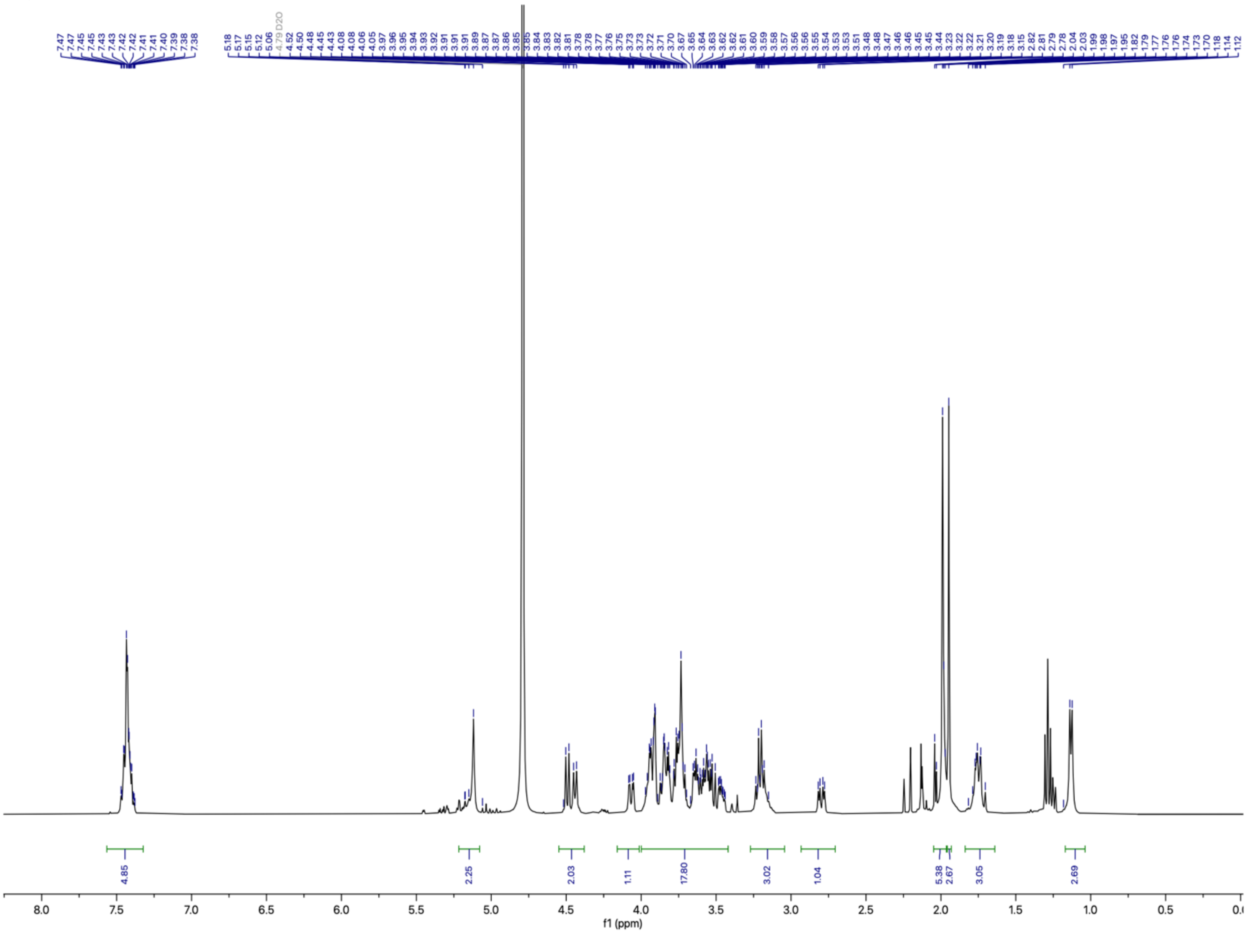

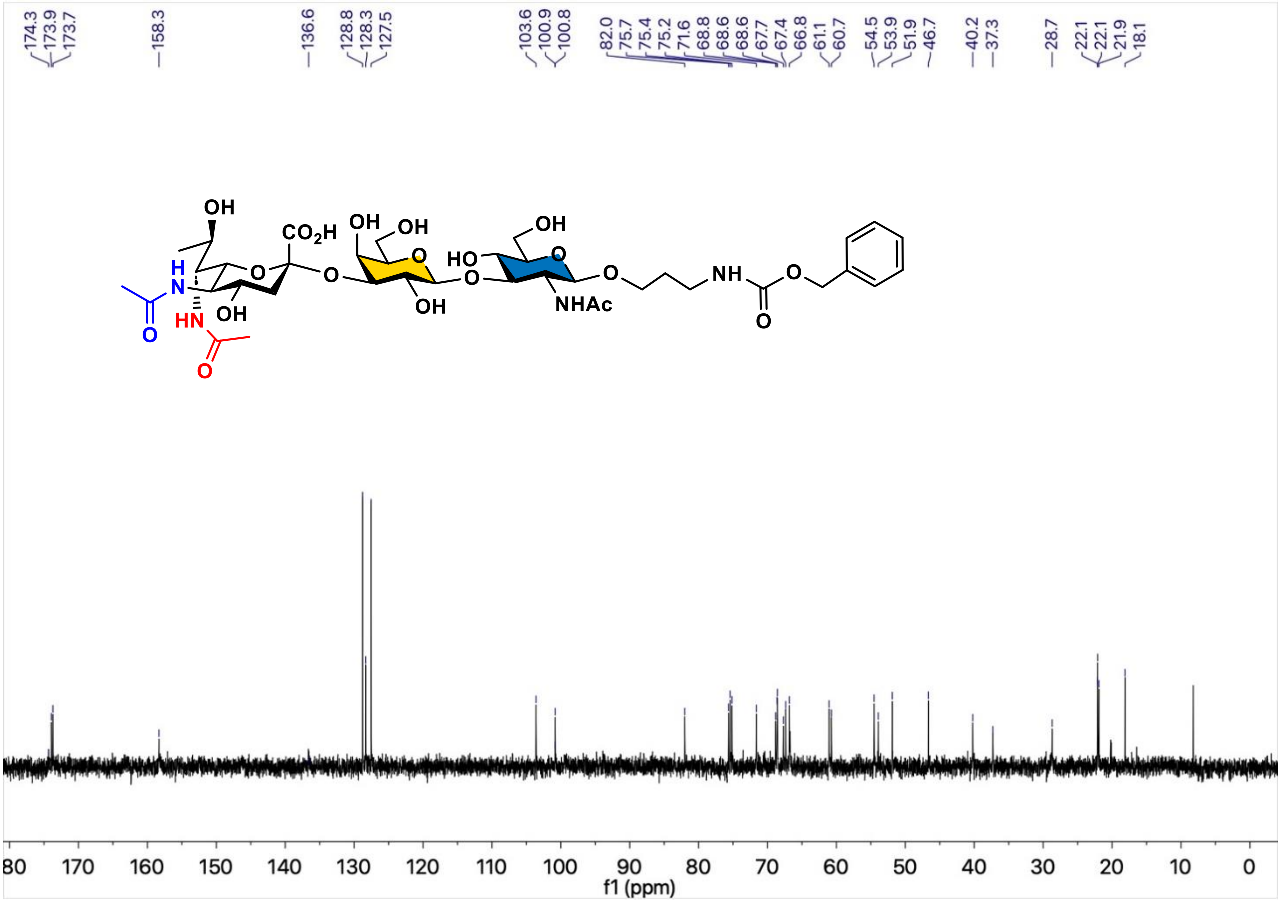

800 MHz ^{1}H and 200 MHz $^{13}C\{^{1}H\}$ NMR spectra of Leg5,7Ac$_2$α2–3Galβ1–3GlcNAcαProNHCbz (**37**) in D_2O.

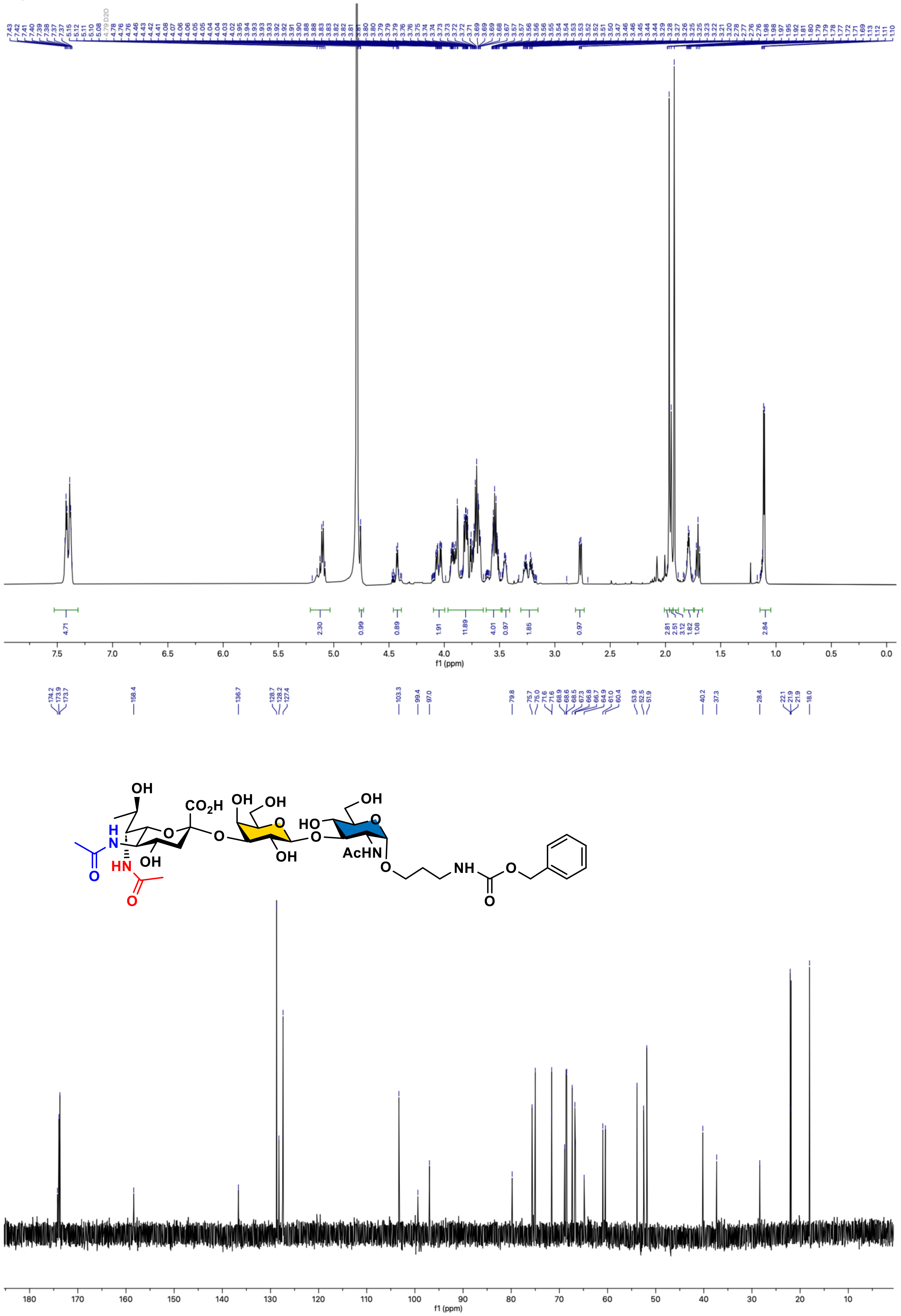

Supplement: Supplementary file 1 [file molecules-29-03980-s001.zip › molecules-3098390-supplementary.pdf]
